# RASSF1A Suppresses Estrogen-Dependent Breast Cancer Cell Growth through Inhibition of the Yes-Associated Protein 1 (YAP1), Inhibition of the Forkhead Box Protein M1 (FOXM1), and Activation of Forkhead Box Transcription Factor 3A (FOXO3A)

**DOI:** 10.3390/cancers12092689

**Published:** 2020-09-21

**Authors:** Sven Roßwag, Gitta Thiede, Jonathan P. Sleeman, Sonja Thaler

**Affiliations:** 1 European Center for Angioscience, Medical Faculty Mannheim, University of Heidelberg, 68167 Mannheim, Germany; sven.rosswag@medma.uni-heidelberg.de (S.R.); gitta.thiede@web.de (G.T.); Jonathan.Sleeman@medma.uni-heidelberg.de (J.P.S.); 2 KIT Campus Nord, Institute for Toxicology and Genetics, 76344 Karlsruhe, Germany

**Keywords:** RASSF1A, ER+ breast cancer development, inhibition of YAP1, regulation of ER-alpha

## Abstract

**Simple Summary:**

The majority of breast cancers express the estrogen receptor alpha (ERα). This receptor is of central importance for breast cancer development and tumor outgrowth, and represents an important target for therapeutic intervention. However, the molecular mechanisms that are responsible for the control of ERα expression and function in the context of breast cancer initiation and progression are complex and yet not fully understood. Here we demonstrate that RASSF1A acts as an inhibitor of ERα-driven breast cancer cell growth through a complex, hierarchically organized network that initially involves suppression of the Hippo effector Yes-associated protein 1 (YAP1), which is followed by inhibition of AKT1 activity, increased FOXO3A activity as well as a blockade of FOXM1 and ERα expression. Together our findings provide important new mechanistic insights into how the loss of RASSF1A contributes to ERα+ breast cancer initiation and progression.

**Abstract:**

The estrogen receptor alpha (ERα) is expressed by the majority of breast cancers and plays an important role in breast cancer development and tumor outgrowth. Although ERα is well known to be a specific and efficient therapeutic target, the molecular mechanisms that are responsible for the control of ERα expression and function in the context of breast cancer initiation and progression are complex and not completely elucidated. In previous work, we have demonstrated that the tumor suppressor RASSF1A inhibits ERα expression and function in ERα-positive breast cancer cells through an AKT-dependent mechanism. Transcriptional activators such as forkhead box protein M1 (FOXM1) and forkhead transcription factor 3A (FOXO3A) and signaling pathways such as the Hippo pathway are also known to modulate ERα expression and activity. Here we report that RASSF1A acts as an inhibitor of ERα-driven breast cancer cell growth through a complex, hierarchically organized network that initially involves suppression of the Hippo effector Yes-associated protein 1 (YAP1), which is followed by inhibition of AKT1 activity, increased FOXO3A activity as well as a blockade of FOXM1 and ERα expression. Together our findings provide important new mechanistic insights into how the loss of RASSF1A contributes to ERα+ breast cancer initiation and progression.

## 1. Introduction

Breast cancer is the most common tumor diagnosed among women, and it is the second leading cause of cancer death worldwide [1]. Estrogen receptors (ER) are expressed in approximately 70% of human breast cancers and play key roles in tumor development and treatment outcome in breast cancer patients [2]. Identification of molecular mechanisms that regulate ER activity and function may facilitate the development of improved breast cancer treatment regimes. However, the cellular and molecular events that regulate ERα and ERβ protein expression and function are complex and not fully elucidated.

The Ras associated domain family 1 isoform A (RASSF1A) is frequently inactivated in breast carcinomas and is associated with estrogen receptor status because aberrant DNA methylation is thought to inactivate RASSF1A more frequently in ER+ breast carcinomas [3,4]. These findings prompted us to investigate a potential functional relationship between RASSF1A and ERα. In previous studies we reported that reconstitution of RASSF1A in MCF7 cells led to decreased ERα levels and reduced sensitivity to estrogen (E2), which was accompanied by induction of cell cycle arrest and senescence [3]. Based on these findings we suggested that RASSF1A acts as a tumor suppressor in ERα+ breast epithelial cells, in part through inhibition of ERα expression and activity, as well as through repression of signaling pathways that are important for E2 independence [3]. However, the molecular mechanisms through which RASSF1A affects ERα expression and function, as well as other proteins that might mediate the tumor-suppressive functions of RASSF1A in breast epithelial cells and during breast cancer initiation remain to be fully elucidated. 

The forkhead transcription factor (FOX) proteins are transcriptional regulators that play a central role during development and adult tissue homeostasis [5]. FOXM1 and FOXO3A are two members of this family that are essential components of oncogenic and tumor-suppressive pathways, respectively [5]. Accurate chronological and steric control of FOXO3A and FOXM1 expression regulates gene expression networks that determine cell fate through the modulation of cell cycle progression, differentiation, and survival [5]. Dysfunction in the regulation of their activity can cause tumor initiation, progression, and resistance to therapy [5].

Phosphorylation of FOXO3A protein by AKT or the serine/threonine-protein kinase serum/glucocorticoid-regulated kinase 1 (SGK1) sequesters the transcription factor in the cytoplasm, leading to its destruction by the ubiquitylation-mediated proteasome pathway [6,7,8,9]. This impairs the activation of FOXO3A-dependent tumor suppressive gene transcriptional programs. ERK and IKKβ also phosphorylate FOXO3A, targeting the protein for degradation by mechanisms involving ubiquitin E3 ligases, such as SKP2 and MDM2 [10,11,12]. In contrast, phosphorylation by AMP-activated protein kinase (AMPK), mammalian Ste20-like kinase (Mst1), the Jun N-terminal (JNK), or p38 MAPKs promotes the nuclear localization of the FOXO3A protein [13,14,15], thus enhancing its ability to transcriptionally regulate proliferative arrest, differentiation, and cell death. Consequently, the deregulation of FOXO3A and other FOX proteins through post-translational modification can directly contribute to tumor initiation, progression, and therapy resistance [16,17,18].

FOXM1 and FOXO3A are decisive for ERα regulation in normal tissues, as well as during ER+ breast cancer initiation, progression, and the development of drug resistance [5]. FOXM1 expression is activated by ERα in the presence of estrogens through direct binding of ERα to an estrogen-response element site within the FOXM1 promoter [19], consistent with the observation that elevated expression of FOXM1 in breast cancer strongly correlates with ERα expression [19,20]. Silencing of FOXM1 abolishes the E2-mediated proliferation of MCF7 cells [20], while ectopic expression of constitutively active FOXM1 abrogates the growth arrest caused by tamoxifen [19]. These findings collectively suggest that FOXM1 is an important regulator of the mitogenic functions of ERα and estrogen in breast tumor cells and that deregulation of FOXM1 subsequently may contribute to anti-estrogen insensitivity [19]. The FOXO3A protein, on the other hand, interacts directly with ERα and ERβ, which inhibits their transcriptional activities and thereby suppresses the expression of ER target genes [21]. Thus, FOXO3A can act as a tumor suppressor [5] and is a downstream target of several oncogenic pathways [9], including the ERK [10], nuclear factor-kB (NF-kB)-inhibitor of NF-kB kinase-β (IKKβ) [11], and PI3K-Akt signaling cascades [6,7,8,9]. Consistently, FOXO3A inhibits estrogen-dependent breast cancer proliferation and suppresses tumorigenesis of MCF7 cells in an animal orthotopic breast tumor model, providing compelling evidence that FOXO3A plays an important role in the suppression of estrogen-dependent breast cancer [21]. Interestingly, FOXO3A is a functional antagonist of FOXM1 [5,22], and thus FOXO3A could conceivably also be an inhibitor of ERα by counteracting FOXM1 activity.

As the silencing of FOXM1 abolishes the E2-mediated proliferation of MCF7 cells [20], and FOXO3A can functionally antagonize FOXM1 [22], we hypothesized that RASSF1A may inhibit FOXM1. Furthermore, RASSF1A inhibits AKT1 [3,23]. As AKT1 is an inhibitor of FOXO3A [6] and FOXO3A is an inhibitor of ERα activity [21], we also hypothesized that RASSF1A may inhibit ERα through mechanisms that involve activation of FOXO3A. Moreover, RASSF1A can activate the Hippo pathway [24,25] and modify the function of the Hippo-kinase effector YAP1 [26]. YAP1 can activate AKT1 and Skp2 leading to increased degradation of FOXO1/3 [27]. We therefore additionally hypothesized that RASSF1A may inhibit AKT1 activity through a mechanism that involves changes in the YAP1 function.

Consistent with these hypotheses, we report here that RASSF1A reduces both FOXM1 and ERα expression and increases FOXO3A activity. Knockdown of FOXM1 in ERα+ MCF7 breast cancer cells led to reduced expression of ERα, cell cycle arrest, and senescence, partially phenocopying the effects of ectopic RASSF1A expression in these cells. Partial knockdown of FOXO3A caused increased outgrowth of colonies and reduced induction of senescence in the presence of RASSF1A in comparison to controls. Knockdown of YAP1 in ERα+ MCF7 breast cancer cells led to reduced expression of ERα and FOXM1, cell cycle arrest, and senescence and inhibition of AKT1 and increased activity of FOXO3A. Based on these findings, we conclude that RASSF1A acts as an inhibitor of ERα expression and function through a hierarchical mechanism that involves suppression of the Hippo kinase effector YAP1 and subsequent inhibition of AKT1 activity, increased FOXO3A activity, as well as the blockade of FOXM1 expression and activity. 

## 2. Results

### 2.1. RASSF1A Causes Decreased Expression of FOXM1 and ERα, and Induces Senescence

**Figure 1:**
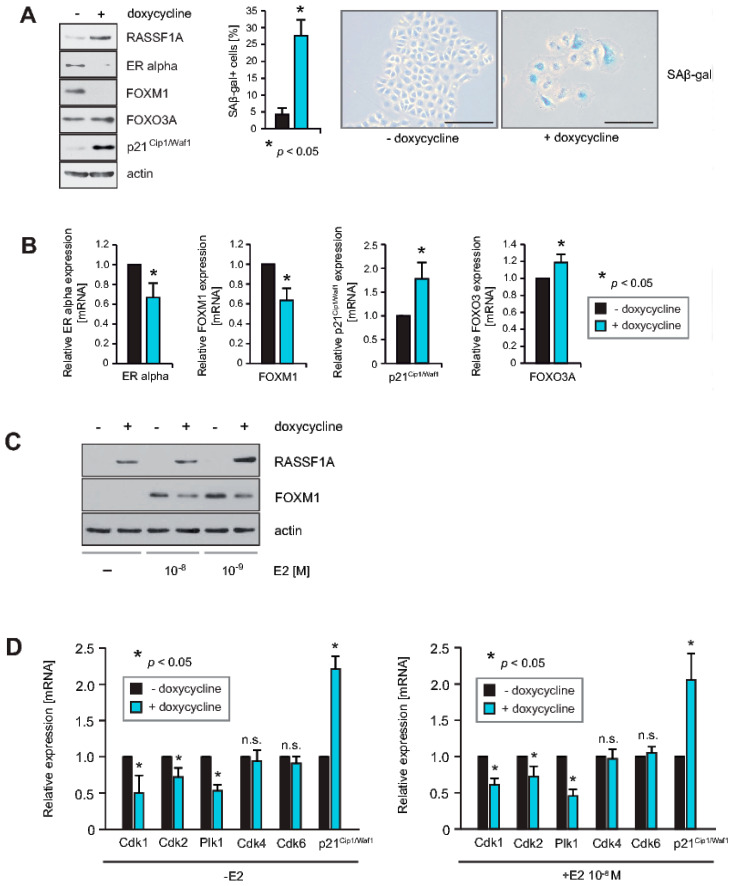
RASSF1A causes decreased expression of FOXM1 and ERα, and induces senescence. (**A**) Induction of RASSF1A expression in conditional RASSF1A MCF7 cells was achieved by culturing cells in the presence of 1 μg/mL doxycycline. Cell extracts from induced and non-induced conditional RASSF1A cells were prepared 48 h after doxycycline administration and were analyzed by immunoblotting using the indicated antibodies. Actin serves as a loading control (left panel). Densitometric quantification of the bands FOXO3A—doxycycline: 100 versus FOXO3A +doxycycline: 173; detailed information about the western blots are given at Figure S1. Conditional RASSF1A cells were plated at equal densities and grown for 6 days in the presence or absence of doxycycline as indicated. RASSF1A-induced senescence was subsequently monitored by SAβ-gal staining (right panel). Bars = 100 μm. The quantification of senescent cells was achieved by counting (middle panel). Mean values ± s.d. of six independent experiments are shown. *p*-values < 0.05 are indicated by asterisks. (**B**) RASSF1A downregulates transcription of ERα and FOXM1 but increases the expression of p21^Cip1/Waf1^ and FOXO3A. Conditional MCF7 cells were grown in the presence or absence of 1 μg/mL doxycycline as indicated. mRNA was harvested 48 h after doxycycline administration. ERα, FOXM1, p21^Cip1/Waf1,^ and FOXO3A transcript levels were analyzed by quantitative PCR. Mean values ± s.d. of four independent experiments are shown. *p*-values < 0.05 are indicated by asterisks. (**C**) FOXM1 expression in MCF7 cells is dependent on E2. Conditional MCF7 cells were grown in the presence or absence of 1μg/mL doxycycline, and in the presence or absence of different E2 concentrations as indicated. Cell extracts prepared 48 h after doxycycline administration and 24 h after E2 exposure were analyzed by immunoblotting with the indicated antibodies. (**D**) Conditional RASSF1A MCF7 cells were grown in the presence or absence of 1 μg/mL doxycycline and in the presence or absence of E2 as indicated. mRNA was harvested 48 h after doxycycline administration and 24 h after E2 exposure. Transcript levels of the indicated genes were analyzed by quantitative PCR. RASSF1A caused reduced expression of Cdk1, 2, and Plk1, whereas no significant reduction of Cdk4 and 6 and increased expression of p21^Cip1/Waf1^ was observed. Similar results were obtained in the absence (left panel) and the presence of E2 (right panel). Mean values ± s.d. of four independent experiments are shown. *p*-values < 0.05 are indicated by asterisks.

As FOXM1 is an important regulator of the mitogenic functions of ERα in breast tumor cells, we hypothesized that RASSF1A may exert some of its suppressive effects on ERα through regulating FOXM1 expression and activity. In agreement with this hypothesis, induction of RASSF1A expression in MCF7 cells that conditionally express RASSF1A upon addition of doxycycline (RASSF1A conditional MCF7 cells) caused decreased expression of ERα and FOXM1 proteins, increased expression of the cell cycle inhibitor p21^Cip1/Waf1,^ and slightly increased expression of FOXO3A and correlated with the induction of cellular senescence (Figure 1A). Further analysis using qPCR confirmed that following RASSF1A induction, transcription of ERα and FOXM1 is reduced, whereas transcription of p21^Cip1/Waf1^ and FOXO3A is increased (Figure 1B). In addition, we also found that FOXM1 expression in MCF7 cells is dependent on E2 (Figure 1C), in accordance with previous reports [19]. Thus, inhibition of endogenous FOXM1 by RASSF1A could be secondary at least in part to the downregulation of ERα by RASSF1A.

FOXM1 activity is strongly dependent on posttranscriptional modifications such as phosphorylation and acetylation [5]. FOXM1 includes phosphorylation sites that are targeted by cell cycle-dependent kinases (Cdks) and Plk1 [5,28,29,30,31,32]. Phosphorylation of FOXM1 is important for inhibition of autorepression [30], activation of mitotic progression [31], and inhibition of senescence [29]. Using qPCR, we found that RASSF1A induction caused decreased expression of Cdk1, 2, and Plk1 in RASSF1A conditional MCF7 cells whereas no inhibition of Cdk4 and 6 was observed (Figure 1D). However, RASSF1A caused increased expression of p21^Cip1/Waf1^ (Figure 1D), as we have reported previously [3]. Since p21^Cip1/Waf1^ binds to Cdk4 and 6 and thereby blocks their activity [33], we conclude that RASSF1A might inhibit Cdk4 and 6 in an indirect mechanism by increasing the expression of p21^Cip1/Waf1^. In turn, this may regulate FOXM1 activity through regulatory phosphorylation. Collectively, these observations suggest that RASSF1A has the potential to inhibit FOXM1 expression and activity both transcriptionally and post-translationally.

### 2.2. Knockdown of FOXM1 Inhibits Expression of ERα and Induces Cell Cycle Arrest and Senescence, Phenocopying the Effects of RASSF1A

**Figure 2:**
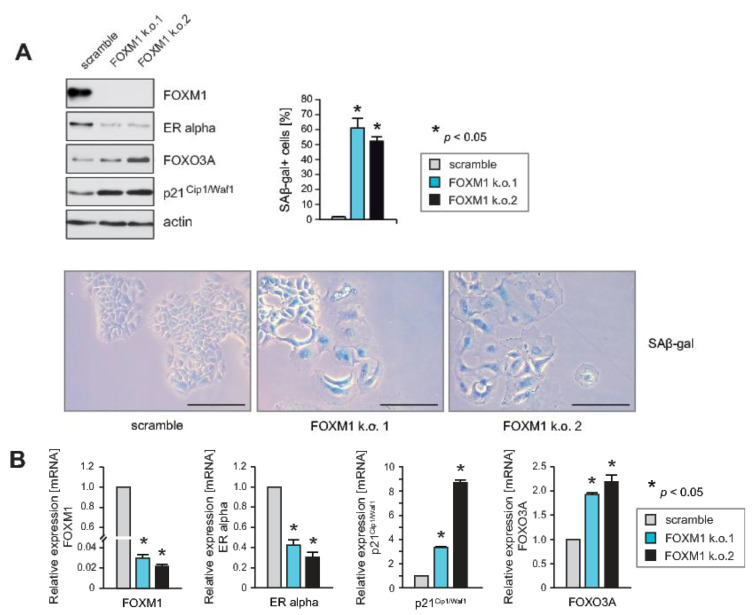
Knockdown of FOXM1 inhibits expression of ERα and induces cell cycle arrest and senescence, phenocopying the effects of RASSF1A. (**A**) Stable knockdown of FOXM1 was performed in parental MCF7 cells using shRNAs (FOXM1k.o.1 and FOXM1k.o.2). Cell extracts were prepared 48 h after lentiviral transduction. Western blots of lysates from the two independent FOXM1 shRNAs (FOXM1k.o.1 and FOXM1k.o.2) in MCF7 cells were probed with the indicated antibodies. Actin served as a loading control (upper left panel). Knockdown of FOXM1 using FOXM1k.o.1 and FOXM1k.o.2 in MCF7 cells induced growth inhibition and senescence as evidenced by SAβ-gal staining 48 h after lentiviral transduction (lower panel). Bars = 100 μm. Quantification of the percentage of SAβ-gal positive cells in two independent knockdowns of FOXM1 expression in MCF7 cells (FOXM1k.o.1 and FOXM1k.o.2) is presented as mean values ± s.d. of three independent experiments. *p*-values < 0.05 are indicated by asterisks (upper right panel). (**B**) Knockdown of FOXM1 in MCF7 cells using the shRNAs FOXM1k.o.1 and FOXM1k.o.2 was performed and mRNA was prepared 48 h after lentiviral transduction. Transcript levels of the indicated genes were analyzed by quantitative PCR. Mean values ± s.d. of three independent experiments are presented. *p*-values < 0.05 are indicated by asterisks.

The above findings suggest that RASSF1A-mediated inhibition of ERα+ breast cancer cell growth may involve inhibition of FOXM1 expression. To determine whether loss of FOXM1 expression functionally phenocopies RASSF1A expression, we used stable knockdown of FOXM1 in parental MCF7 cells. Reduced expression of FOXM1 was verified by immunoblotting (Figure 2A) and qPCR (Figure 2B). Similar to induction of RASSF1A expression, knockdown of FOXM1 caused growth arrest and cellular senescence (Figure 2A), and reduced expression of ERα while increasing expression of p21^Cip1/Waf1^ and FOXO3A (Figure 2A,B). Taken together these observations are consistent with the notion that suppression of FOXM1 expression by RASSF1A plays an important role in RASSF1A-induced cell cycle arrest and senescence. 

### 2.3. Ectopic Expression of FOXM1 Does Not Rescue Cells from RASSF1A-Mediated ERα Suppression and Cell Cycle Arrest

**Figure 3:**
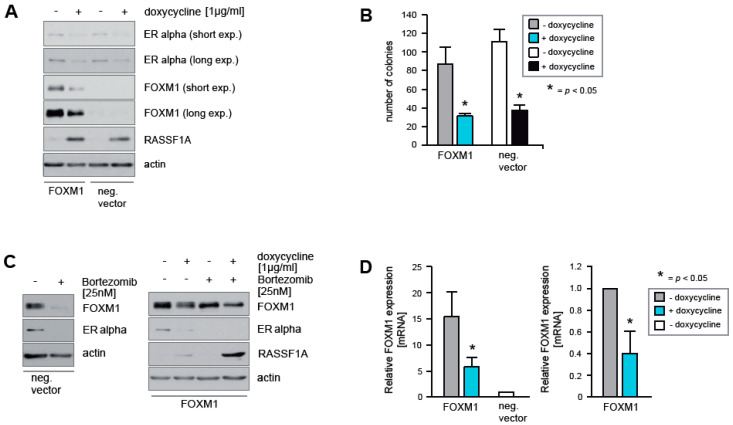
Ectopic expression of FOXM1 cannot rescue RASSF1A-mediated ERα suppression and cell cycle arrest. (**A**) RASSF1A-inducible MCF7 cells were retrovirally transduced with a FOXM1 expression vector or with an empty vector. Both cell lines were cultured in the presence or absence of 1 μg/mL doxycycline as indicated. After 48 h cells were harvested and subsequently lysates were analyzed by immunoblotting using the indicated antibodies. (**B**) Equal numbers of knockout and negative control cells were grown in the presence or absence of 1 μg/mL doxycycline as indicated. After 14 days, colonies were fixed at equal time points and counted. Mean values ± s.d. of four independent experiments are presented. *p*-values < 0.05 are indicated by asterisks. (**C**) MCF7 cells were cultured in the presence or absence of 25 nM Bortezomib. After 30 h cells were harvested and lysates were analyzed by immunoblotting using the indicated antibodies (left panel). Conditional RASSF1A MCF7 cells were grown in the presence or absence of 1 μg/mL doxycycline and in the presence or absence of Bortezomib as indicated. Cells were harvested 48 h after doxycycline administration and 30 h after Bortezomib exposure. Lysates were analyzed by immunoblotting using the indicated antibodies (right panel). (**D**) Conditional RASSF1A MCF7 cells with ectopic FOXM1 expression were cultured in the presence or absence of 1 μg/mL doxycycline as indicated and mRNA was prepared after 48 h. Transcript levels of FOXM1 were analyzed by quantitative PCR. Transcript levels of endogenous FOXM1 from non-induced empty vector-carrying conditional MCF7 cells were used to quantify ectopic FOXM1 transcripts in the presence and absence of RASSF1A (left panel). Transcript levels of FOXM1 in induced conditional RASSF1A cells ectopically expressing FOXM1 relative to FOXM1 transcript levels in non-induced conditional cells (right panel). Mean values ± s.d. of four independent experiments are presented. *p*-values < 0.05 are indicated by asterisks.

As RASSF1A causes decreased expression of FOXM1 (Figure 1A), we next investigated whether ectopic expression of FOXM1 is sufficient to rescue MCF7 cells from RASSF1A-mediated suppression of ERα and growth arrest. Ectopic expression of FOXM1 in RASSF1A conditional MCF7 cells neither abolished RASSF1A-mediated down-regulation of ERα expression (Figure 3A), nor inhibited RASSF1A-induced growth arrest (Figure 3B). Unexpectedly, RASSF1A inhibited the ectopic expression of the FOXM1 protein (Figure 3A). Under physiological conditions, inhibition of the proteasome by Bortezomib causes reduced transcription of FOXM1 and ERα [34], leading to lower endogenous levels of these proteins (Figure 3C left panel). In MCF7 cells ectopically expressing FOXM1 in the absence of RASSF1A, no decrease in FOXM1 protein levels was observed in the presence of Bortezomib as expected due to ectopic expression being under the control of the CMV promoter. However, RASSF1A expression reduced FOXM1 protein levels in both the presence and absence of Bortezomib (Figure 3C right panel). Thus, RASSF1A decreases levels of ectopic FOXM1 protein independently of proteasome-mediated destruction.

In further experiments, we found that RASSF1A suppressed ectopic expression of FOXM1 at the transcriptional level, as assessed by qPCR (Figure 3D). To rule out unspecific inhibitory effects of RASSF1A on the promoter driving ectopic FOXM1 expression, we used two different promoters to enforce FOXM1 expression. However, RASSF1A suppressed ectopic expression of FOXM1 independently of the promoter sequence used. This phenomenon means that it is not possible to assess whether ectopic FOXM1 expression can rescue cells from RASSF1A-mediated suppression of ERα and growth arrest. Importantly, however, it indicates that RASSF1A likely affects FOXM1 transcript stability, for example by up-regulation of inhibitory RNAs that target FOXM1 transcripts.

### 2.4. FOXO3A Is Required for RASSF1A-Mediated Growth Arrest

**Figure 4:**
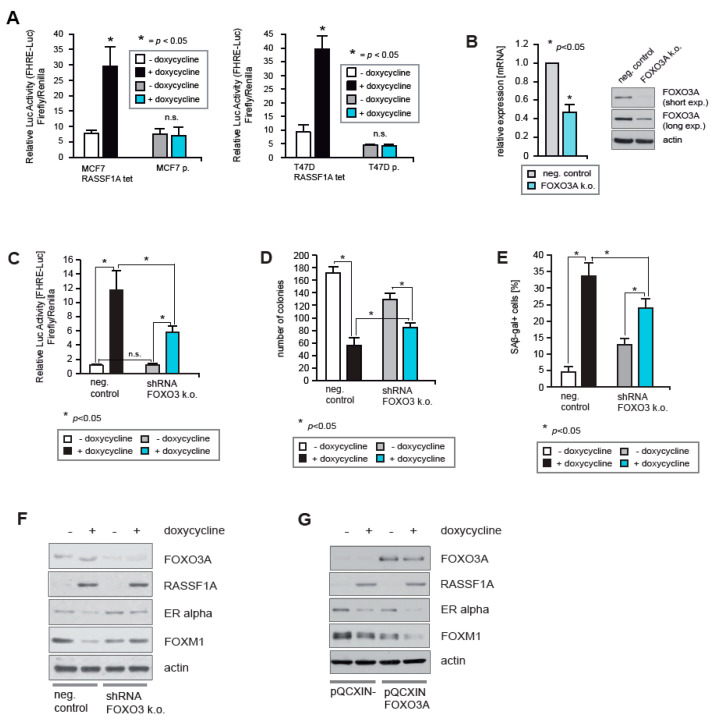
FOXO3A is required for RASSF1A-mediated growth arrest and senescence. (**A**) Conditional MCF7 (MCF7 RASSF1A tet) and T47D (T47D RASSF1A tet) cells were transfected with the FOXO luciferase reporter plasmid FHRE luc [6]. After 24 h RASSF1A expression was induced by administration of 1 μg/mL doxycycline. Subsequent luciferase expression was measured 48 h later. To exclude any unspecific activation of FOXO by doxycycline the same experimental procedure was performed using parental MCF7 (MCF7p.) and T47D (T47Dp.) cells. Mean values ± s.d. of three independent experiments are presented. *p*-values < 0.05 are indicated by asterisks. (**B**) Stable knockdown of FOXO3A in conditional RASSF1A MCF7 cells using shRNA (FOXO3A k.o.1). mRNA and protein lysates from the cell extracts were prepared 16 days after lentiviral transduction and selection with neomycin. Reduced expression of FOXO3A in conditional RASSF1A FOXO3A k.o. cells were evidenced by quantitative PCR (left panel) and immunoblotting using the indicated antibodies (right panel). Mean values ± s.d. of four independent experiments are presented. *p*-values < 0.05 are indicated by asterisks. (**C**) Conditional RASSF1A FOXO3A k.o. and negative control cells were transfected with the FHRE-Luc reporter plasmid [6]. After 24 h RASSF1A expression was induced by administration of 1 μg/mL doxycycline. Subsequent luciferase expression was measured 48 h later. (**D**) Equal numbers of conditional RASSF1A FOXO3A k.o. and negative control cells were cultured in the presence or absence of 1 μg/mL doxycycline. After 15 days, colonies were fixed and counted at equivalent time points. Mean values ± s.d. of four independent experiments are presented. *p*-values < 0.05 are indicated by asterisks. (**E**) Equal numbers of conditional RASSF1A FOXO3A k.o. and control cells were cultured in the presence or absence of 1 μg/mL doxycycline. After 6 days, colonies were fixed and counted at equivalent time points. Mean values ± s.d. of four independent experiments are presented. *p*-values < 0.05 are indicated by asterisks. (**F**) FOXO3A knockdown and negative control conditional RASSF1A cells were either cultured with or without 1 μg/mL doxycycline for 48 h. Afterward cells were harvested and lysates were analyzed by immunoblotting using the indicated antibodies. (**G**) Ectopic FOXO3A expressing or empty vector-carrying conditional RASSF1A cells were established by retroviral transduction. Both cell lines were cultured in the presence or absence of 1 μg/mL doxycycline, and lysates were analyzed by immunoblotting using the indicated antibodies.

To determine whether RASSF1A also affects ERα expression and activity in breast cancer cells through activation of FOXO3A transcriptional activity, MCF7 and T47D cells that conditionally express RASSF1A [3] were transiently transfected with the FHRE-Luc reporter plasmid [6] that is transcriptionally activated by the O subgroup of FOX proteins (FOXO proteins). The cells were cultured in the presence of doxycycline to induce RASSF1A expression, or without doxycycline as a control. After 48 h the cells were harvested, and the activity of the reporter was measured using luciferase assays. This revealed that FOXO transcriptional activity is increased as a consequence of RASSF1A induction (Figure 4A). No non-specific activation of the reporter construct was detected in parental MCF7 and T47D cells upon doxycycline treatment (Figure 4A). 

To investigate whether FOXO3A is responsible for RASSF1A-induced FOXO transcriptional activity, we employed a stable knockdown of FOXO3A in the RASSF1A conditional MCF7 cells. All shRNAs tested gave an only partial reduction of FOXO3A expression. The shRNA that generated the strongest reduction of FOXO3A expression was used to perform functional analysis. Reduced expression of FOXO3A was evidenced by immunoblotting and qPCR (Figure 4B). In comparison to controls, FOXO3A knockdown substantially reduced FOXO transcriptional activity upon induction of RASSF1A expression (Figure 4C), demonstrating that at least the majority of RASSF1A-induced FOXO transcriptional activity is mediated by FOXO3A.

To test whether FOXO3A is important for RASSF1A-mediated growth arrest, equal numbers of FOXO3A knockout and negative control RASSF1A conditional cells were cultured in the presence or absence of doxycycline. Approximately two weeks later colonies were fixed and counted at equivalent time points. Knockout cells formed more colonies than negative control cells in the presence of RASSF1A (Figure 4D), indicating that FOXO3A is required for RASSF1A-mediated growth arrest. To determine whether FOXO3A is also important for RASSF1A-mediated induction of senescence, equal numbers of FOXO3A knockout and control RASSF1A conditional cells were again cultured in the presence or absence of doxycycline. After 6 days the cells were fixed and senescent cells were quantified. In the presence of RASSF1A, the FOXO3A knockout cells exhibited a lower percentage of senescent cells compared to the control cells (Figure 4E), indicating that FOXO3A is also important for RASSF1A-mediated growth arrest. 

To investigate whether FOXO3A is also important for RASSF1A-mediated inhibition of ERα and FOXM1 expression, FOXO3A knockout and negative control RASSF1A conditional cells were cultured in the presence or absence of doxycycline. After 48 h the cells were harvested and lysates were analyzed by immunoblotting. Partial knockdown of FOXO3A in RASSF1A conditional MCF7 cells resulted in increased levels of both ERα and FOXM1 relative to controls upon induction of RASSF1A expression (Figure 4E), suggesting that FOXO3A knockdown abrogates RASSF1A-mediated suppression of ERα and FOXM1 expression. Consistently, while ectopic expression of FOXO3A did not change the expression of ERα and FOXM1 in the absence of RASSF1A, in the presence of RASSF1A ectopic expression of FOXO3A, augmented the reduction of ERα and FOXM1 expression (Figure 4F). Thus, FOXM1 and FOXO3A mutually antagonize each other in a manner that is regulated by RASSF1A. 

### 2.5. RASSF1A Increases FOXO3A Transcriptional Activity through Suppression of AKT-Mediated Inhibitory Phosphorylation and Increased Expression of FOXO3A

**Figure 5:**
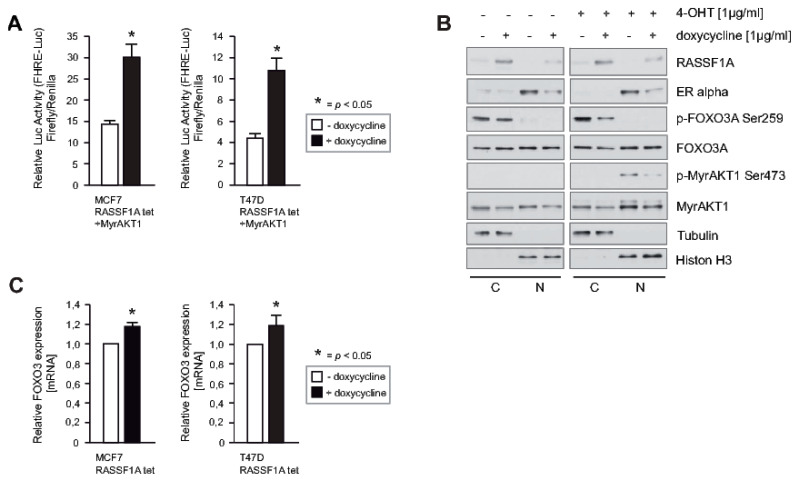
RASSF1A increases FOXO3A transcriptional activity through the suppression of AKT-mediated inhibitory phosphorylation and increased expression of FOXO3A. (**A**) Conditional RASSF1A MCF7 (left panel) and conditional RASSF1A T47D cells (right panel) [3] were co-transfected with a eukaryotic MyrAKT1 expression construct [35] and the FHRE Luc plasmid [6]. 24 h after transfection cells were cultured in the presence or absence of 1 μg/mL doxycycline for 48 h to induce RASSF1A expression. Subsequently, luciferase expression was analyzed. Mean values ± s.d. of four independent experiments are presented. *p*-values < 0.05 are indicated by asterisks. (**B**) Conditional RASSF1A MCF7 cells transduced to express MyrAKT1ERtam or empty vector were cultured in the presence or absence of 1 μg/mL doxycycline for 48 h as indicated. To investigate the temporal influence of RASSF1A upon phosphorylation of MyrAKT1 and subsequent phosphorylation of FOXO3A, conditional RASSF1A cells were cultured in the absence or presence of 1 μg/mL 4-Hydroxytamoxifen (4-OHT) for activation of MyrAKT1 [36] as indicated. Cells were prepared 40 min after the administration of 4-OHT. Subsequent immunoblotting of the cytoplasmic and nuclear extracts were probed with the indicated antibodies. (**C**) Conditional RASSF1A MCF7 and T47D cells were cultured in the presence or absence of 1 μg/mL doxycycline for 48 h. Afterward, cells were harvested for mRNA preparation. Transcript levels of FOXO3A were analyzed by quantitative PCR. Mean values ± s.d. of four independent experiments are presented. *p*-values < 0.05 are indicated by asterisks.

AKT phosphorylates S253 and S315 of the FOXO3A protein, leading to proteasomal degradation of FOXO3A [9]. However, in the presence of myristylated AKT1 (MyrAKT1) [35] that is constitutively activated, we still found that RASSF1A increased FOXO transcriptional activity (Figure 5A). Since RASSF1A inhibits AKT activity through inhibiting phosphorylation of AKT S473 [3], we speculated that RASSF1A may increase FOXO3A transcriptional activity by suppressing AKT-mediated phosphorylation of the FOXO3A protein. To determine whether this is the case, MCF7 cells conditional for RASSF1A expression were stably transduced with a retroviral construct that directs inducible expression of MyrAKT1 (MyrAKT1ERtam) [36]. These cells were cultured in the presence or absence of doxycycline. To activate the conditional MyrAKT1, 4-Hydroxytamoxifen (4-OHT) was added. After 20 min 4-OHT induction, cells were harvested, and nuclear and cytoplasmic extracts were prepared. Phosphorylation of AKT S473 and FOXO3A S253 was monitored by immunoblotting. Consistent with our previous findings, RASSF1A caused decreased phosphorylation of MyrAKT1 on S473. RASSF1A also decreased phosphorylation of FOXO3A S253 (Figure 5B). Furthermore, when MCF7 and T47D cells conditional for RASSF1A were cultured in the presence or absence of doxycycline, increased transcription of endogenous FOXO3A was observed upon RASSF1A induction (Figure 5C). Collectively these findings suggest that RASSF1A increases FOXO3A transcriptional activity through suppression of AKT-mediated inhibitory phosphorylation of the FOXO3A protein, and through increasing transcription of FOXO3A.

### 2.6. RASSF1A and FOXO3A Cause Decreased ERα and ERβ Activity

**Figure 6:**
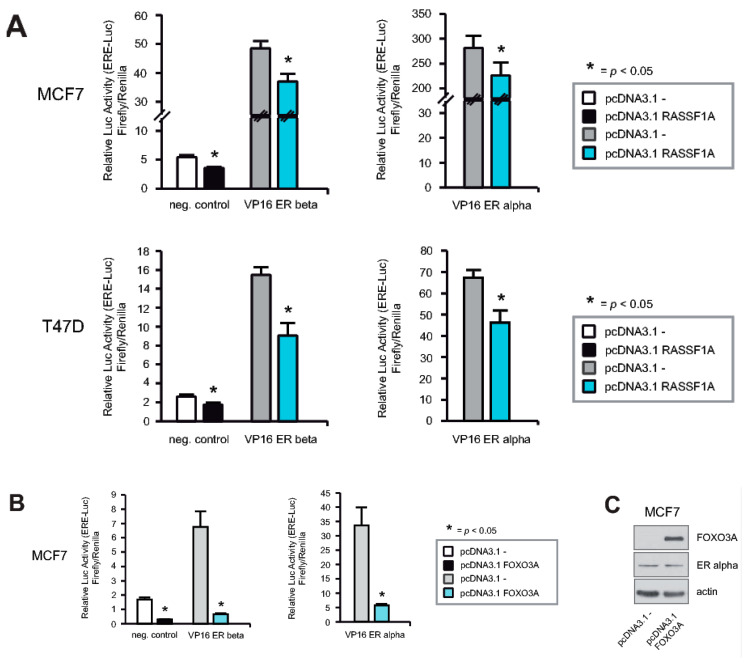
RASSF1A and FOXO3A cause decreased ERα and ERβ activity. (**A**) Parental MCF7 cells (upper panel) and parental T47D cells (lower panel) were co-transfected with a RASSF1A expression construct or empty vector together with either ERα [37] or ERβ [37] and an ER luciferase reporter plasmid (3× ERE TATA luc) [38]. 48 h after transfection luciferase expression was measured. Mean values ± s.d. of five independent experiments are presented. *p*-values < 0.05 are indicated by asterisks. (**B**) Parental MCF7 cells were co-transfected with a FOXO3A expression construct [6] or empty vector in combination with either ERα [37] or ERβ [37] and the ERE driving luciferase reporter plasmid (3× ERE TATA luc) [38]. 48 h after transfection luciferase expression was measured. Mean values ± s.d. of five independent experiments are presented. *p*-values < 0.05 are indicated by asterisks. (**C**) Parental MCF7 cells were transfected with a FOXO3A expression construct [6] or an empty vector. 48 h after transfection cells were harvested and lysates were analyzed by immunoblotting using the indicated antibodies.

As both RASSF1A and FOXO3A inhibit expression of ERα target genes [3,21], we hypothesized that RASSF1A may affect the transcriptional activity of ERα through increasing FOXO3A transcriptional activity. If so, then RASSF1A expression should inhibit both ERα and ERβ, as FOXO3A suppresses both ERα and ERβ activity [21]. As shown in Figure 6A, co-transfection of RASSF1A together with either ERα or ERβ expression constructs [37,38] into parental MCF7 and T47D cells resulted in inhibition of the transcriptional activity of both ERα and ERβ, as evidenced by measuring transcriptional activation of a luciferase reporter construct (3× ERE TATA luc) [38] that is driven by tandem estrogen response elements (EREs). Co-transfection of FOXO3A [6] together with either ERα or ERβ also inhibited the transcriptional activity of both ERs (Figure 6B). Ectopic expression of FOXO3A in MCF7 cells did not change ERα protein levels (Figure 6C), consistent with previous reports showing that FOXO3A inhibits ER function without affecting their expression [21] and our own observation (Figure 4F). Based on the results shown in Figure 4A,E,F, we conclude that RASSF1A inhibits ERα activity at least in part through modulation of FOXO3A activity.

### 2.7. Neither IKKβ nor AKT1 Can Override the RASSF1A-Mediated Increase in FOXO3A Activity 

**Figure 7:**
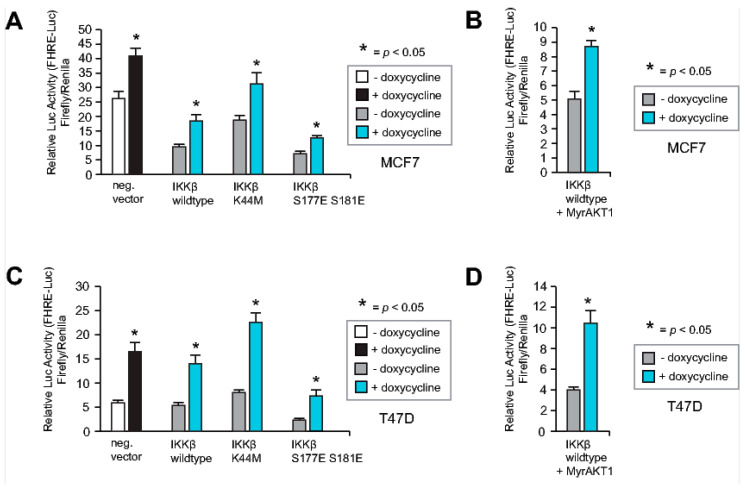
Neither IKKβ nor AKT1 can override the RASSF1A-mediated increase in FOXO3A activity. (**A**) Conditional RASSF1A MCF7 [3] and (**C**) conditional RASSF1A T47D cells [3] were co-transfected with the FOXO3A reporter plasmid FHRE-Luc [6] and either IKKβ wildtype or IKKβ mutant constructs [39]. Transfected cells were cultured in the presence or absence of 1 μg/mL doxycycline and luciferase expression was measured at equal time points approximately 48 h after RASSF1A induction. Mean values ± s.d. of five independent experiments are presented. *P*-values < 0.05 are indicated by asterisks. (**B**) Conditional RASSF1A MCF7 [3] and (**D**) conditional RASSF1A T47D cells [3] were co-transfected with the FOXO3A reporter plasmid FHRE-Luc [6] and IKKβ wildtype [39] in combination with a constitutive active MyrAKT1 expression construct [35]. Increased activity of FOXO3A in conditional MCF7 and T47D cells was still observed after RASSF1A induction, even in the presence of both constitutive active MyrAKT1 [35] and wildtype IKKβ [39].

FOXO3A activity and cellular localization can be regulated by an AKT-independent mechanism, in which IkB kinase (IKKβ) phosphorylates FOXO3A, leading to ubiquitin-dependent proteasomal degradation [11]. We, therefore, investigated whether RASSF1A increases FOXO3A transcriptional activity even in the presence of IKKβ. To this end, we co-transfected the MCF7 and T47D cells conditional for RASSF1A expression with the FHRE-Luc reporter plasmid [6], and either IKKβ wildtype or IKKβ mutant constructs [39]. Transfected cells were cultured in the presence or absence of doxycycline and harvested 48 h later. FOXO transcriptional activity in the cells was measured using luciferase assays. This revealed increased luciferase expression upon RASSF1A induction in the presence of all IKKβ constructs (Figure 7A,B). Co-transfection revealed that RASSF1A still increased FOXO transcriptional activity in conditional MCF7 and T47D cells even in the presence of both MyrAkt1 [35] and IKKβ [39] (Figure 7C,D). These findings suggest that RASSF1A activates FOXO3A through the inhibition of several signaling cascades that block the FOXO3A function. RASSF1A may additionally increase FOXO3A transcriptional activity through the activation of proteins that post-transcriptionally modify and thereby activate FOXO3A. 

### 2.8. RASSF1A Inhibits YAP1, and Knockdown of YAP1 Suppresses Akt1 Activity, Inhibits Expression of ERα and FOXM1, and Increases Levels of FOXO3A

RASSF1A inhibits activation of AKT1 [3,23] (Figure 5B). The mechanism through which RASSF1A inhibits AKT1 remains to be elucidated. RASSF1A modifies the functional consequences of YAP1 [26], a protein that activates AKT and Skp2, leading to increased degradation of FOXO1/3 [27]. We, therefore, investigated whether RASSF1A regulates AKT1, FOXO3A, ERα, and FOXM1 through modification of YAP1 activity.

**Figure 8:**
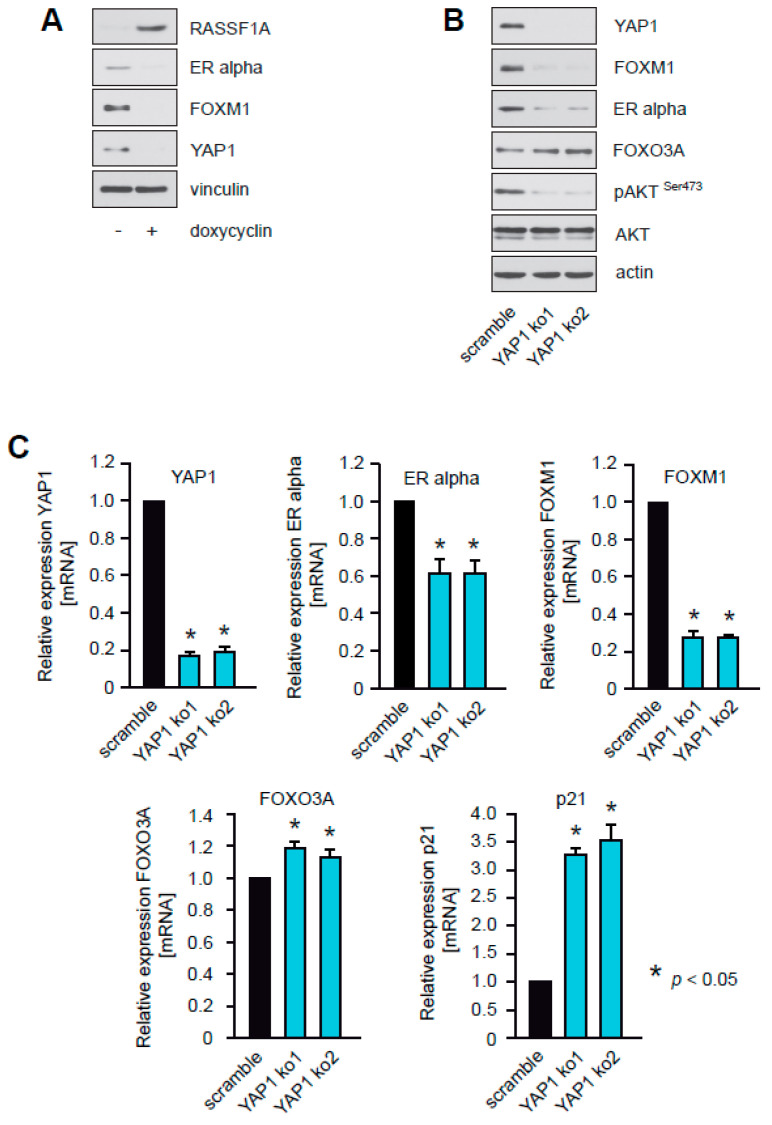

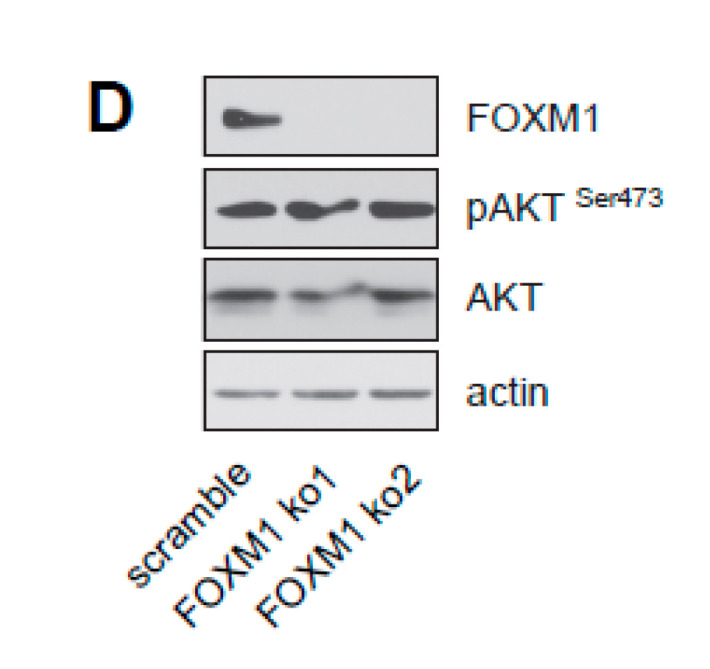
RASSF1A inhibits YAP1, and knockdown of YAP1 suppresses AKT1 activity, inhibits expression of ERα and FOXM1, and increases levels of FOXO3A. (**A**) Induction of RASSF1A expression in conditional RASSF1A MCF7 cells was achieved by culturing cells in the presence of 1 μg/mL doxycycline. Cell extracts from induced and non-induced conditional RASSF1A cells were prepared 48 h after doxycycline administration and were analyzed by immunoblotting using the indicated antibodies. Actin served as a loading control (left panel). (**B**) Stable knockdown of YAP1 was performed in parental MCF7 cells using shRNAs (YAP1k.o.1 and YAP1k.o.2). Cell extracts were prepared 48 h after lentiviral transduction. Western blots of lysates from the two independent YAP1 shRNAs (YAP1k.o.1 and YAP1k.o.2) in MCF7 cells were probed with the indicated antibodies. Actin served as a loading control (upper left panel). Densitometric quantification of the western blot bands scramble: 100; YAP1ko1: 134; YAP1ko2: 177; detailed information about western blots are given at Figure S7. (**C**) Knockdown of FOXM1 in MCF7 cells using the shRNAs FOXM1k.o.1 and FOXM1k.o.2 was performed and mRNA was prepared 48 h after lentiviral transduction. Transcript levels of the indicated genes were analyzed by quantitative PCR. Mean values ± s.d. of three independent experiments are presented. *P*-value < 0.05 are indicated by asterisks. (**D**) Stable knockdown of FOXM1 was performed in parental MCF7 cells using shRNAs (FOXM1k.o.1 and FOXM1k.o.2). Cell extracts were prepared 48 h after lentiviral transduction. Western blots of lysates from the two independent FOXM1 shRNAs (FOXM1k.o.1 and FOXM1k.o.2) in MCF7 cells were probed with the indicated antibodies. Actin served as a loading control.

To verify that RASSF1A affects YAP1, RASSF1A conditional cells were cultured in the presence or absence of doxycycline. Changes in the amounts of YAP1 upon RASSF1A expression were monitored by immunoblotting. As expected, the induction of RASSF1A expression caused decreased expression of YAP1 protein (Figure 8A).

To determine whether loss of YAP1 expression functionally phenocopies RASSF1A expression, we used stable knockdown of YAP1 in parental MCF7 cells. Reduced expression of YAP1 was verified by immunoblotting (Figure 8B) and qPCR (Figure 8C). Similar to induction of RASSF1A expression, knockdown of YAP1 caused reduced expression of ERα and FOXM1, decreased levels of pAKT S473 and increased expression of p21^Cip1/Waf1^ and slightly increased expression of FOXO3A (Figure 8B,C). In contrast to knockdown of YAP1, knockdown of FOXM1 did not inhibit AKT, nor reduce levels of pAKT S473 (Figure 8D) suggesting that RASSF1A-mediated inhibition of YAP1 is the initial step that leads to suppression of FOXM1 and ERα expression, inhibition of AKT activity, and activation of FOXO3A. Taken together these observations are consistent with the notion that suppression of YAP1 expression by RASSF1A plays a pivotal role in RASSF1A-induced cell cycle arrest and senescence, and explain mechanistically how the loss of RASSF1A contributes to ERα+ breast cancer initiation and progression.

## 3. Discussion

**Figure 9:**
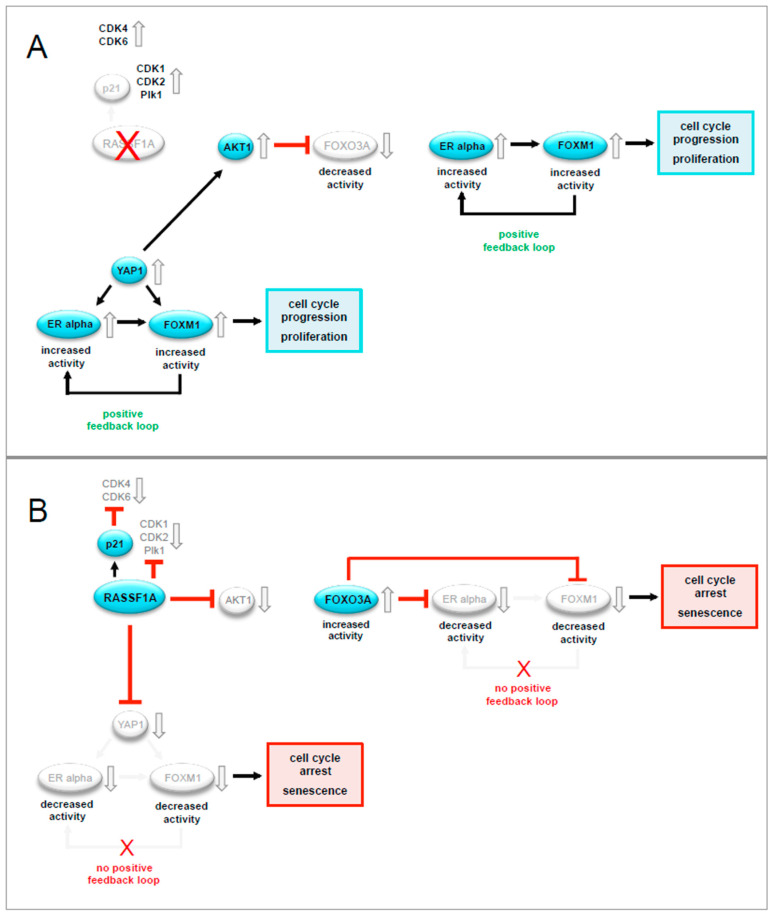
Schematic model of a possible molecular network between RASSF1A, the Hippo pathway effector YAP1, AKT, FOXO3A, FOXM1, and ERα based on the findings in this paper and on the published literature. (**A**) In the absence of RASSF1A, the Hippo pathway effector YAP1 is active and affects the expression and activity of ERα and FOXM1, directly and indirectly. First, YAP1 causes expression of FOXM1 and the ERα leading to increased levels of both proteins. Second, YAP1 also causes increased activity of AKT1 [27]. AKT1 phosphorylates FOXO3A and subsequently curtail its activity. FOXO3A can counteract ERα activity [21]. Under the influence of E2, ERα binds to the FOXM1 promoter and induces FOXM1 expression. Vice versa, FOXM1 can bind to the ERα promoter, maintaining, or increasing ERα expression in a positive feedback loop between both proteins. This feedback loop is sustained in the presence of E2. As FOXM1 is a key regulator of cell cycle progression, deregulated FOXM1 expression leads to uncontrolled proliferation and resistance against senescence. (**B**) RASSF1A interferes with the network outlined in **(A)** by inhibiting YAP1 and AKT1 signaling, thereby increasing the activity of FOXO3A. FOXO3A can inhibit ERα and FOXM1 by two different mechanisms: (i) FOXO3A can directly bind to the ERα and thereby inhibit its activity. This causes the suppression of ERα target gene expression [21]. In the presence of E2, ERα binds to the FOXM1 promoter and induces FOXM1 expression [19]. Thus, the RASSF1A-mediated increase in FOXO3A activity can suppress FOXM1 expression through inhibition of ERα activity. (ii) FOXO3A and FOXM1 negatively regulate each other, as FOXO3A is a functional antagonist of FOXM1 [22] and FOXM1 negatively regulates FOXO3A [5,22]. Increased levels and activity of FOXO3A in the presence of RASSF1A, therefore, lead to the replacement of FOXM1 on the FHRE of the FOXM1 promoter with activated FOXO3A, which results in sustained inhibition of FOXM1 expression [22]. In addition to these FOXO3A-dependent mechanisms, RASSF1A suppresses expression of Cdk1, 2, and Plk1 and increases expression of the Cdk4 and 6 inhibitor p21^Cip1/Waf1^. As Plk1 and Cdk1, 2, 4, and 6 phosphorylate and thereby activate FOXM1 [28,29,30,31,32]. RASSF1A might also inhibit FOXM1 activity by suppressing the expression of these kinases. Taken together, these mechanisms determine that in the context of events that transform mammary epithelial cells, RASSF1A acts as an inhibitor of ERα driven breast cancer cell growth through a complex, hierarchical organized network that involves first suppression of the Hippo effector YAP1 and subsequent inhibition of AKT1, increased FOXO3A activity as well as the blockade of FOXM1 and ERα expression, thereby inducing growth arrest and senescence. Based on these findings, we conclude that loss of RASSF1A is an important initial step towards ERα+ breast cancer initiation and progression.

FOXM1 and FOXO3A are transcriptional regulators that play a central role during embryonic development and adult tissue homeostasis. Deregulation of these proteins has a direct impact on cancer initiation, progression, and drug resistance [5]. Control of these proteins is achieved through cell-specific expression but is also fine-tuned by many post-translational modifications and through interaction with specific co-factors. Besides being conventional transcription factors, they can also function as transcriptional regulators by interacting with other transcription factors and epigenetic effectors [5]. Here we show that the tumor suppressor RASSF1A exerts many of its effects through suppression of FOXM1 and increasing FOXO3A activity. Our findings show that RASSF1A regulates these FOX family members at a number of levels through a complex network of molecular interactions that includes counter-regulation of FOXM1 and FOXO3A by each other, further proteins such as AKT, and components of the Hippo-pathway such as YAP1 (see Figure 9).

Our data are consistent with published observations that FOXM1 and FOXO3A have opposite roles in cancer. Thus, while FOXM1 functions as an oncogene, FOXO3A behaves like a tumor suppressor in most contexts [5]. Our results, therefore, suggest that RASSF1A acts as a guardian that keeps FOXM1 and FOXO3A activity and function in balance. Thus, the loss or inhibition of RASSF1A that is observed in many types of cancer [40] would be predicted to disturb the homeostatic regulation of FOXM1 and FOXO3A, leading to oncogenic stimulation through deregulated FOXM1 activity, which explains why the loss of RASSF1A can contribute to cancer initiation and progression. 

Our data suggest that RASSF1A can suppress FOXM1 activity at several levels. We show that RASSF1A down-regulates FOXM1 transcription, and suggest that RASSF1A-induced FOXO3A is a major mediator of these effects, as partial knockdown of FOXO3A abrogated RASSF1A-mediated suppression of FOXM1 expression (Figure 8A). We also show that RASSF1A suppresses the activity of AKT1 that can phosphorylate and inhibit the activity of FOXO3A, indicating that RASSF1A is an activator of FOXO3A. Activated FOXO3A is a functional antagonist of FOXM1, as it replaces FOXM1 on the FHRE of the FOXM1 promoter, leading to sustained inhibition of FOXM1 expression [22]. In addition, RASSF1A might also suppress FOXM1 transcription by inhibiting c-Myc expression, as RASSF1A inhibits E2-mediated expression of c-Myc [3] and FOXM1 is predicted to be a transcriptionally regulated by c-Myc [41].

Our results also suggest that RASSF1A might up-regulate inhibitory RNAs that target FOXM1 transcripts because RASSF1A decreased levels of FOXM1 transcripts even when FOXM1 was ectopically expressed under the control of promoters that do not contain FHRE or ERα binding elements. We note with interest that the miRNAs miR-370 and miR-34a suppress the expression of FOXM1 [42,43]. Future research will focus on whether these or other inhibitory RNAs are up-regulated by RASSF1A. 

In addition to transcriptional regulation, our results further suggest that RASSF1A may modulate FOXM1 activity through post-translational modification. We found that RASSF1A decreased expression of CDK1 and 2 and Plk1, which phosphorylate and thereby activate FOXM1 [5,28,29,30,31,32]. Furthermore, RASSF1A up-regulated p21^Cip1/Waf1^ expression, which is an inhibitor of CDK4 and 6 [33]. Cdk4 and 6 stabilize and activate FOXM1, thereby maintaining the expression of G1/S phase genes and protecting cells from senescence [32]. 

RASSF1A is frequently inactivated in breast carcinomas and is associated with estrogen receptor status [3,4]. We have previously shown that RASSF1A suppresses ERα and reduces sensitivity to E2, leading to growth arrest and senescence [3]. FOXM1 can physiologically regulate ERα in breast carcinoma cells [19] through activating transcription of the ERα promoter, thereby increasing ERα expression [20]. Although FOXM1 and FOXO3A have been suggested to cooperate in the regulation of ERα gene transcription [20], FOXO3A has been reported to inhibit ERα activity and suppresses the outgrowth of ERα+ breast cancer [21]. This is consistent with our findings that RASSF1A counteracts FOXM1 and upregulates FOXO3A, which would block FOXM1-dependent ERα activity, suggesting that the effects of RASSF1A on ERα expression may be mediated at least in part by inhibition of FOXM1. On the other hand, ERα promotes FOXM1 gene transcription in the presence of estrogens through direct binding of ERα to an estrogen-response element site within the FOXM1 promoter [19], consistent with our own observations (Figure 1C). Thus, an alternative mechanism may be that RASSF1A-dependent suppression of ERα is responsible for decreased FOXM1 expression.

Knockdown of FOXM1 increased FOXO3A expression (Figure 2B), suggesting that FOXM1 can counteract the upregulation of FOXO3A expression by RASSF1A. A number of observations suggest that FOXM1 can also counteract RASSF1A through epigenetic mechanisms. The most prominent mechanism by which RASSF1A expression is lost in breast and many other types of cancer is an epigenetic modification of the RASSF1A promoter sequence [41]. Interestingly, FOXM1 itself was shown to promote DNA methylation through increasing expression of the DNA methyltransferase DNMT3B via the SWI/SNF2-like helicase HELLS, thereby inhibiting the expression of tumor suppressor genes [44,45]. It is therefore highly significant that DNMTB3 is recruited to the RASSF1A promoter, leading to hypermethylation and silencing of RASSF1A expression [46].

This study is consistent with the notion that FOXM1 and FOXO3A participate in a mutual counterregulatory network that determines ERα expression and activity (see Figure 9). RASSF1A maintains the balance between FOXM1 and FOXO3A under normal physiological conditions. Loss of RASSF1A during tumorigenesis results in increased oncogenic FOXM1 activity largely due to altered FOXO3A function resulting in increased ERα activity.

Our data suggest that activation of FOXO3A by RASSF1A is mediated by RASSF1A-dependent suppression of AKT1 activity. They also suggest that RASSF1A counteracts AKT1 activity and thereby activates FOXO3A through inhibition of YAP1. Consistent with this notion, RASSF1A expression in MCF7 cells caused reduced amounts of YAP1 (Figure 8A). Furthermore, knockdown of YAP1 in MCF7 cells phenocopied the consequences of enforced RASSF1A expression, including AKT1 inhibition, modestly increased FOXO3A levels as well as suppression of ERα and FOXM1 expression (Figure 8C). RASSF1A activates the Hippo pathway through activating the Hippo kinases Mst1, Lat2 [47], Mst2, and subsequent activation of Lats1 [48]. As YAP1 is a target of the Hippo pathway, RASSF1A might suppress the growth of ERα+ breast cancer cells through activation of the Hippo kinases, leading to inhibition of YAP1. In addition, we and others have observed that RASSF1A causes increased phosphorylation of p38 (Supplementary information 1A) [49]. As p38 can phosphorylate FOXO3A thereby fostering nuclear translocation and transcriptional activation of FOXO3A, we hypothesize that RASSF1A might increase transcriptional activation of FOXO3A and thereby repress FOXM1 expression through p38. We also observed that RASSF1A decreases Plk1, another protein that inhibits FOXO3A activity [50]. Thus, RASSF1A might additionally activate FOXO3A through inhibition of Plk1 (Figure 1D and Supplementary information 1B).

Taken together our observations suggest that RASSF1A acts as an inhibitor of ERα driven breast cancer cell growth through a complex, hierarchically organized network that involves first suppression of the Hippo effector YAP1 and subsequent inhibition of AKT1, increased FOXO3A activity as well as the blockade of FOXM1 and ERα expression, thereby inducing growth arrest and senescence. Based on these findings, we conclude that the loss of RASSF1A is an important initial step towards ERα+ breast cancer initiation and progression.

## 4. Materials and Methods

### 4.1. Plasmids and Reagents

Details about the antibodies, shRNA, primer sequences, plasmids, and reagents used in this study can be found in the Supplementary Methods File.

### 4.2. Cell Culture and Viral Transduction 

MCF7, T47D, HEK293T, and Phoenix-GP were purchased from ATCC. Unless otherwise indicated, the cell lines were maintained in RPMI supplemented with 10% fetal bovine serum (Takara Clontech, Heidelberg, Germany), 1% L-glutamine, and 1% penicillin/streptomycin. The packaging cell lines Phoenix-GP and HEK293T were used for the generation of retro-or lentiviruses following standard calcium phosphate protocols. Cells were selected with puromycin or neomycin. For all experiments, pooled transduced selected cell clones were used. Transduction efficiencies were monitored by flow cytometric detection of EGFP expression. 

### 4.3. Other Methods

Western blot analysis, FACS analysis, and clonogenic assays were performed as previously described [3,23]. Quantitative RT-PCR was performed with Sybr Green (Invitrogen, Karlsruhe, Germany) according to the manufacturer’s instructions. Detailed information about all western blotscan be found at Figures S1–S7.

### 4.4. Luciferase Assay Using the Dual Glow System (Promega) 

The transient transfection of the FHRE luc reporter plasmid [6] was used for the analysis of FOXO transcriptional activity. For analysis of ERα or β transcriptional activity, transient transfection of the ER luc reporter plasmid (3× ERE TATA Luc) [38] together with either the ERα or ERβ expression constructs [37,38] was employed (for more details see Supplementary Materials). Firefly luciferase expression was normalized to Renilla luciferase expression according to the manufacturer’s instructions (Promega, Heidelberg, Germany). Transfection was performed with Fugene 6 (Promega, Heidelberg, Germany) according to the manufacturer’s instructions.

### 4.5. Cell Cycle Analysis

Cell cycle analysis was performed through direct DNA staining with propidium iodide (PI) (Carl Roth GmbH, Karlsruhe, Germany). Cells were resuspended in hypotonic fluorochrome solution (50 μg/mL PI in 0.1% sodium citrate plus 0.1% Triton X-100 (Sigma Aldrich, Taufkirchen, Germany), then placed at 4 °C in the dark for 1 h before flow cytometry analysis.

### 4.6. Statistical Analysis

Differences between experimental groups were assessed using the Student’s *t*-test (Statistical Analysis System, Release 9.3). *p* values < 0.05 were considered significant.

## 5. Conclusions

The results of this study suggest that RASSF1A suppresses ERα-driven breast carcinogenesis through a hierarchical mechanism, in which RASSF1A represses YAP1 and subsequent inhibits AKT1 activity, resulting in increased FOXO3A activity as well as the blockade of FOXM1 and ERα expression (see Figure 9). RASSF1A-mediated inhibition of YAP1 led to inhibition of FOXM1 through inhibition of FOXM1 and ERα gene expression, as well as through inhibition of AKT1, which resulted in the activation of FOXO3A and subsequent antagonism of FOXM1. Taken together, this study has delineated the mechanistic complexity through which loss of RASSF1A serves an important initial step towards ERα+ breast cancer initiation.

## Supplementary Materials

The following are available online at https://www.mdpi.com/2072-6694/12/9/2689/s1, Figure S1: RASSF1A causes decreased expression of FOXM1 and ERα, and induces senescence, Figure S2: Knockdown of FOXM1 inhibits expression of ERα and induces cell cycle arrest and senescence, phenocopying the effects of RASSF1A, Figure S3: Ectopic expression of FOXM1 cannot rescue RASSF1A-mediated ERα suppression and cell cycle arrest, Figure S4: FOXO3A is required for RASSF1A-mediated growth arrest and senescence, Figure S5: RASSF1A increases FOXO3A transcriptional activity through the suppression of AKT-mediated inhibitory phosphorylation and increased expression of FOXO3A, Figure S6: RASSF1A and FOXO3A cause decreased ERα and ERβ activity, Figure S7: RASSF1A inhibits YAP1, and knockdown of YAP1 suppresses AKT1 activity, inhibits expression of ERα and FOXM1, and increases levels of FOXO3A.
