# Supplementary material for: RASSF1A Suppresses Estrogen-Dependent Breast Cancer Cell Growth through Inhibition of the Yes-Associated Protein 1 (YAP1), Inhibition of the Forkhead Box Protein M1 (FOXM1), and Activation of Forkhead Box Transcription Factor 3A (FOXO3A)
Source: Cancers (Basel). 2020 Sep 21;12(9):2689. doi: 10.3390/cancers12092689 (PMC7566002; doi:10.3390/cancers12092689)
Supplement: Supplementary file 1 [file cancers-12-02689-s001.pdf]

Supplementary Materials

RASSF1A Suppresses Estrogen-Dependent Breast Cancer Cell Growth through Inhibition of the Yes-Associated Protein 1 (YAP1), Inhibition of the Forkhead Box Protein M1 (FOXM1), and Activation of Forkhead Box Transcription Factor 3A (FOXO3A)

Sven Roßwag, Gitta Thiede, Jonathan P. Sleeman and Sonja Thaler

Figure S1: RASSF1A causes decreased expression of FOXM1 and ERα, and induces senescence

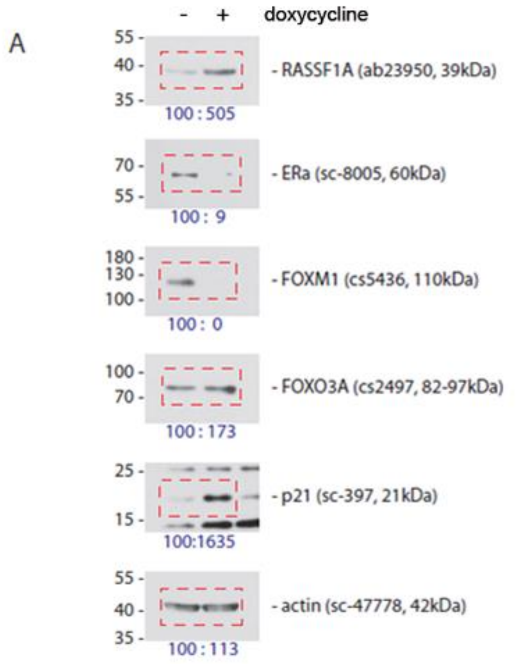

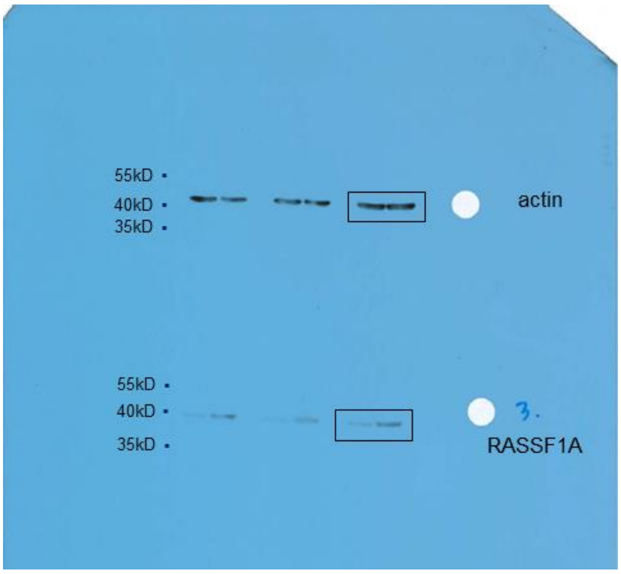

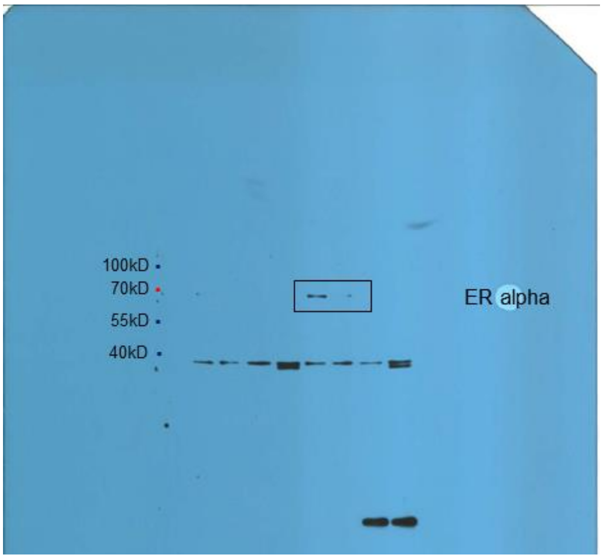
100kD
70kD
55kD
40kD
ER alpha

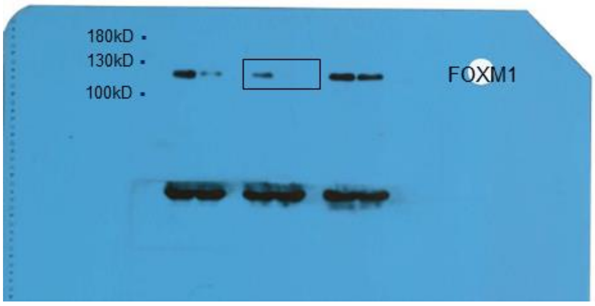
180kD
130kD
100kD
FOXM1

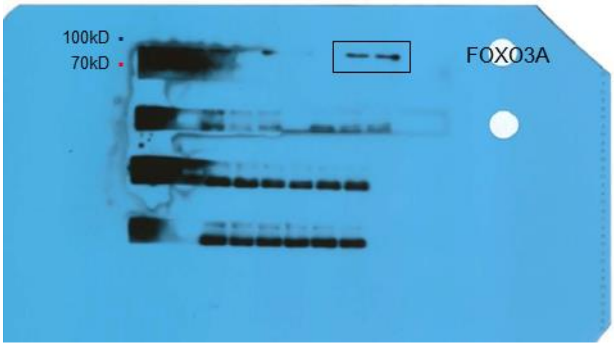
100kD
70kD
FOXO3A

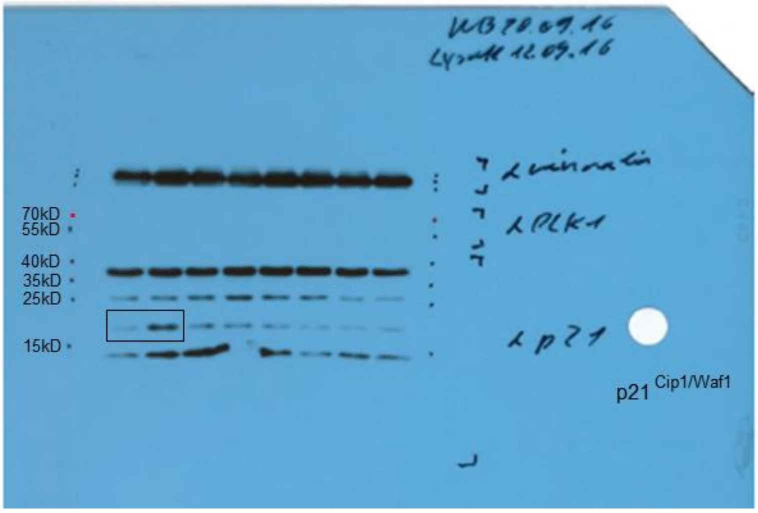
Lysate 12.09.16
70kD
55kD
40kD
35kD
25kD
15kD
PLK1
p21
p21 Cip1/Waf1

Figure S1: RASSF1A causes decreased expression of FOXM1 and ERα, and induces senescence

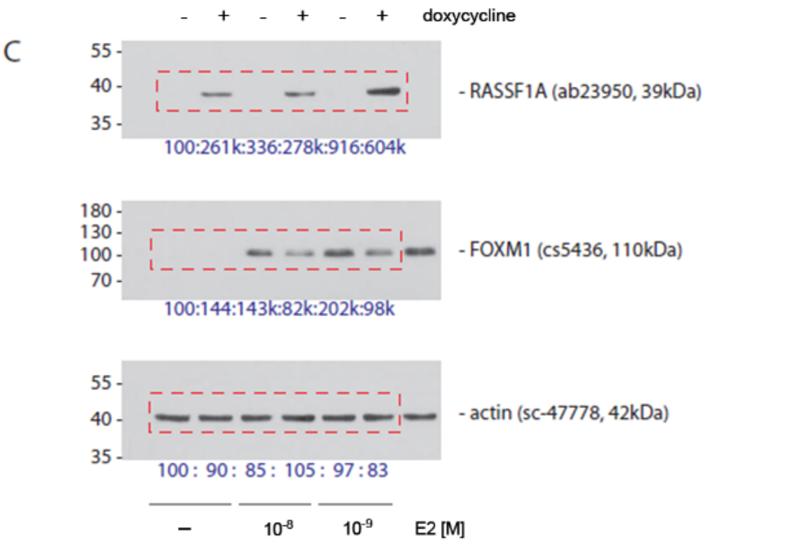

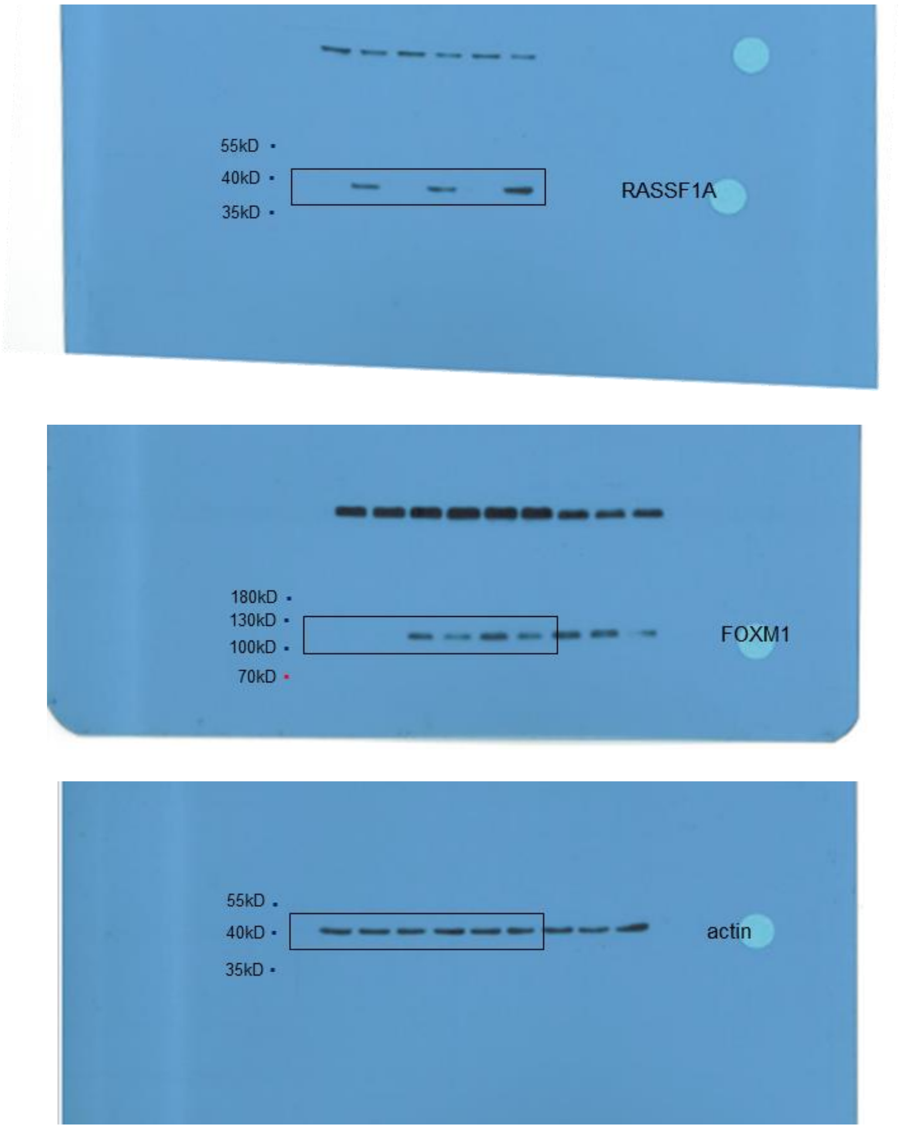

Figure S1. RASSF1A causes decreased expression of FOXM1 and ERα, and induces senescence.

A

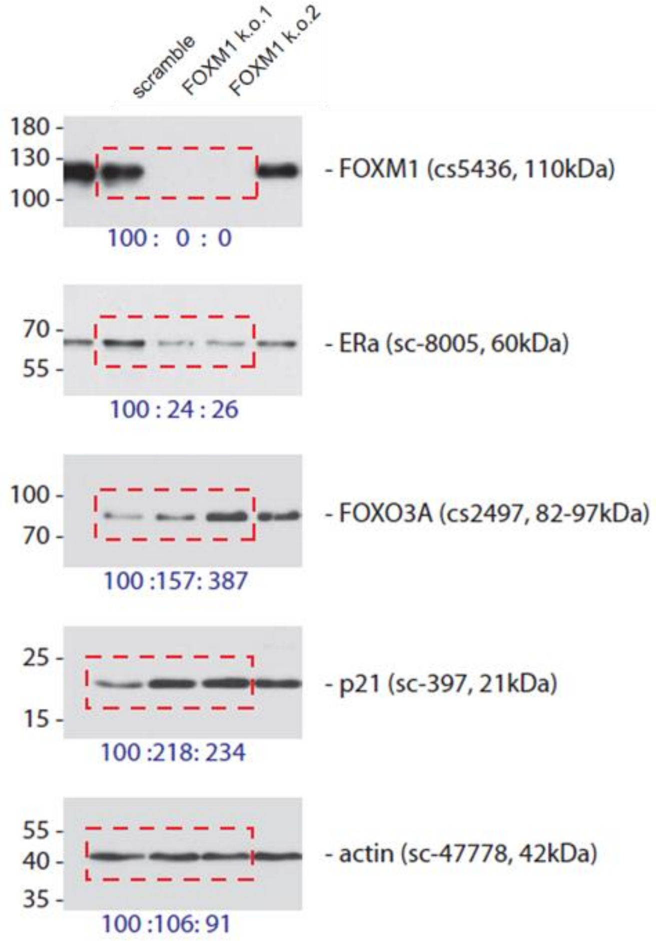
scramble
FOXM1 k.o.1
FOXM1 k.o.2
180
130
100
- FOXM1 (cs5436, 110kDa)
100 : 0 : 0
70
55
- ERa (sc-8005, 60kDa)
100 : 24 : 26
100
70
- FOXO3A (cs2497, 82-97kDa)
100 :157: 387
25
15
- p21 (sc-397, 21kDa)
100 :218: 234
55
40
35
- actin (sc-47778, 42kDa)
100 :106: 91

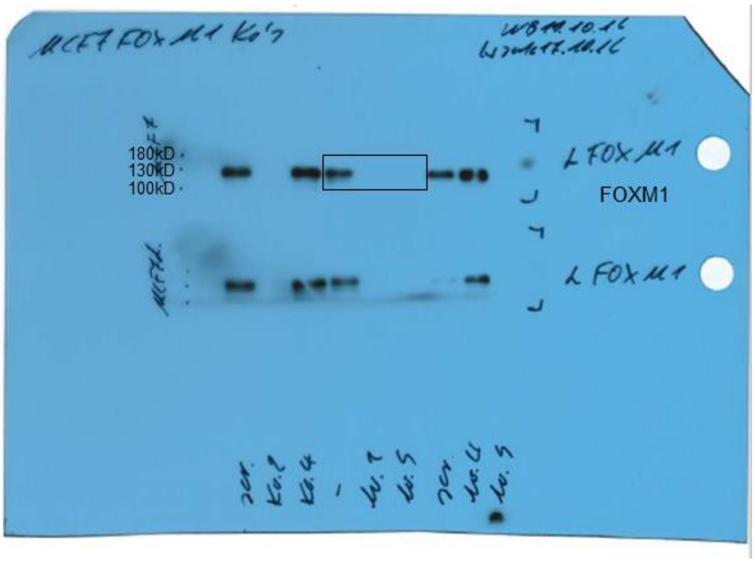
180kD
130kD
100kD
FOXM1

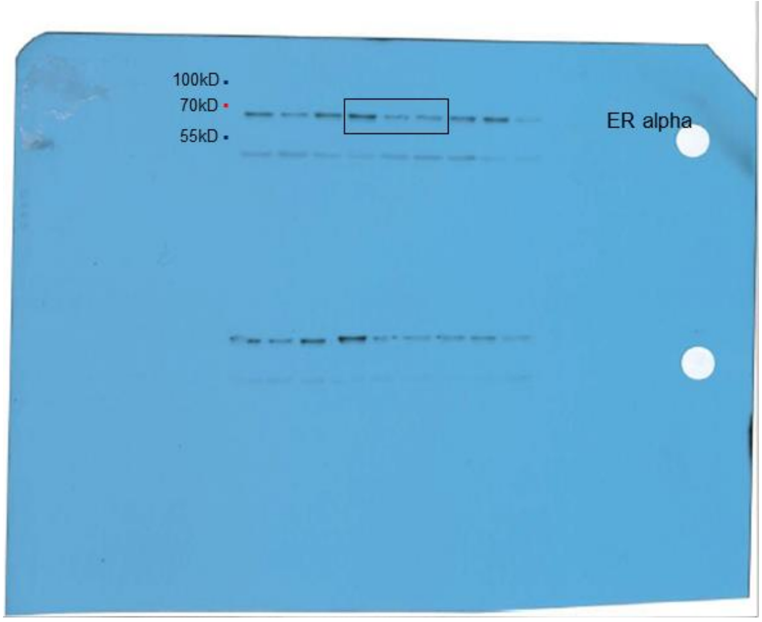
100kD
70kD
55kD
ER alpha

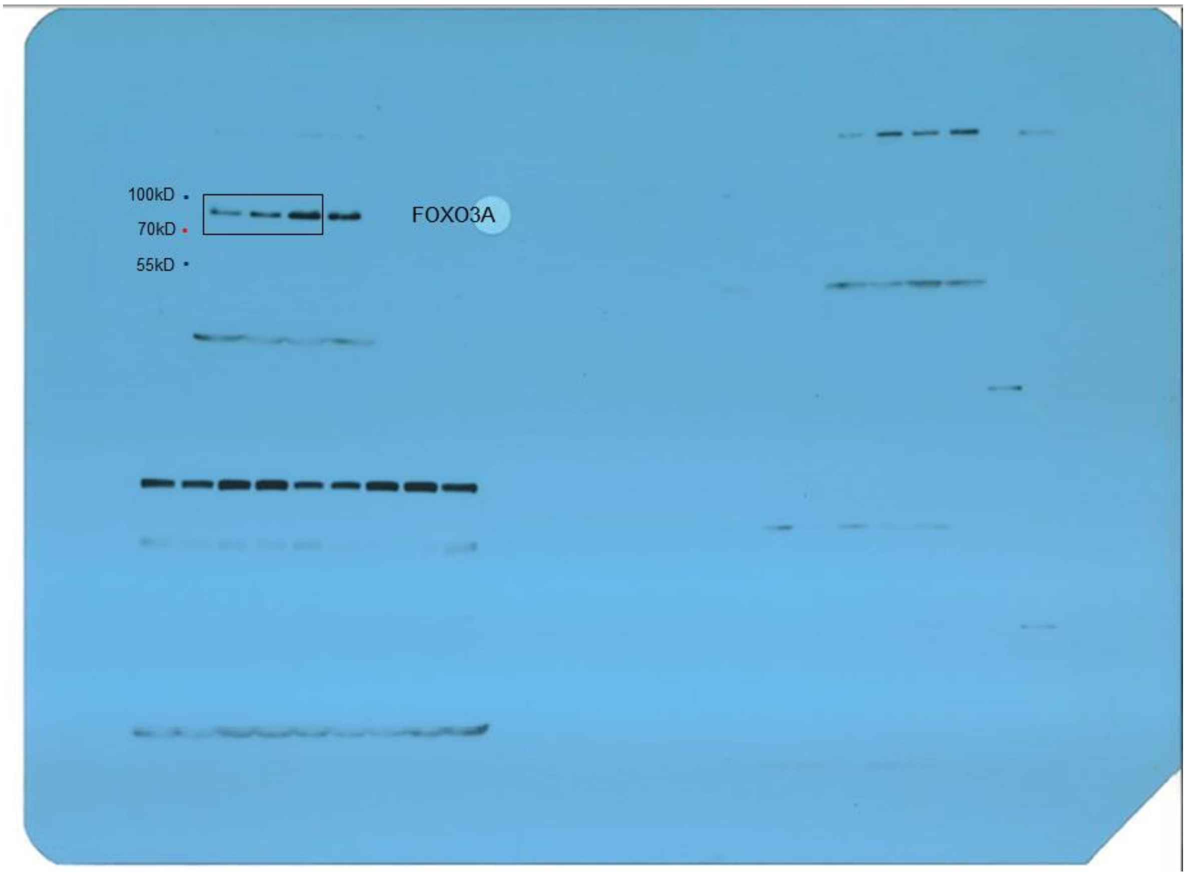
100kD
70kD
55kD
FOXO3A

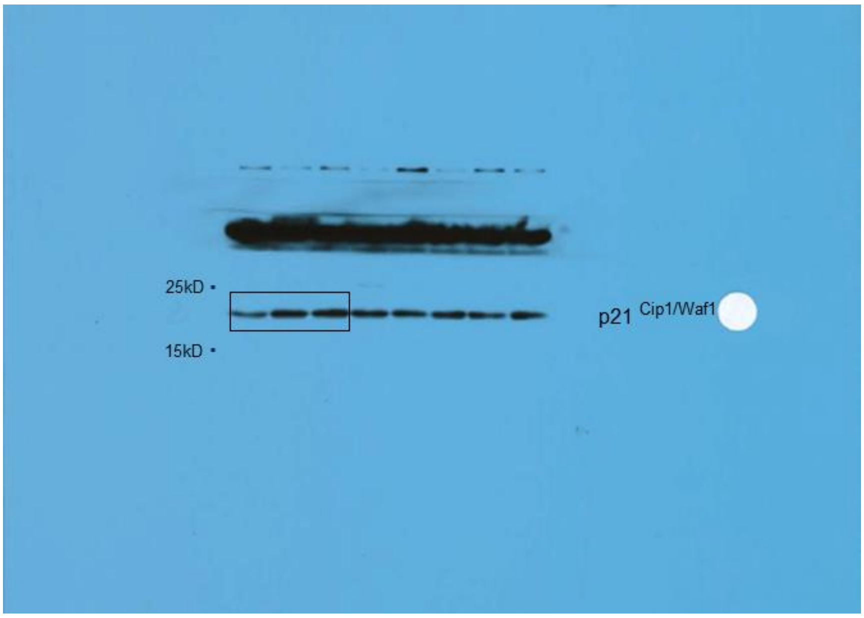

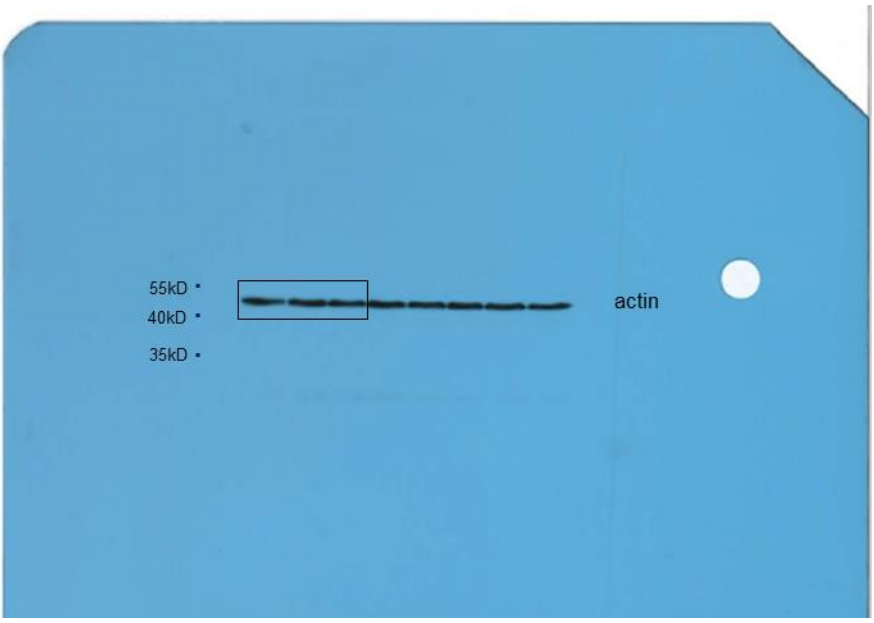

Figure S2. Knockdown of FOXM1 inhibits expression of ERα, and induces cell cycle arrest and senescence, phenocopying the effects of RASSF1A.

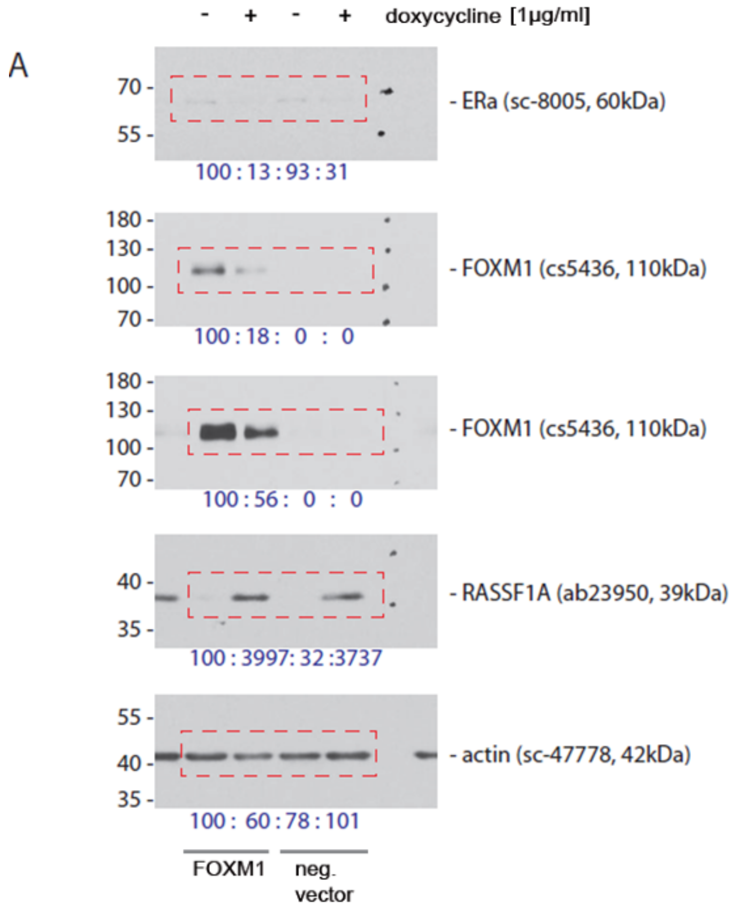

- + - + doxycycline [1µg/ml]
A
70 -
55 -
- ERa (sc-8005, 60kDa)
100 : 13 : 93 : 31
180 -
130 -
100 -
70 -
- FOXM1 (cs5436, 110kDa)
100 : 18 : 0 : 0
180 -
130 -
100 -
70 -
- FOXM1 (cs5436, 110kDa)
100 : 56 : 0 : 0
40 -
35 -
- RASSF1A (ab23950, 39kDa)
100 : 3997: 32 :3737
55 -
40 -
35 -
- actin (sc-47778, 42kDa)
100 : 60 : 78 : 101
FOXM1
neg. vector

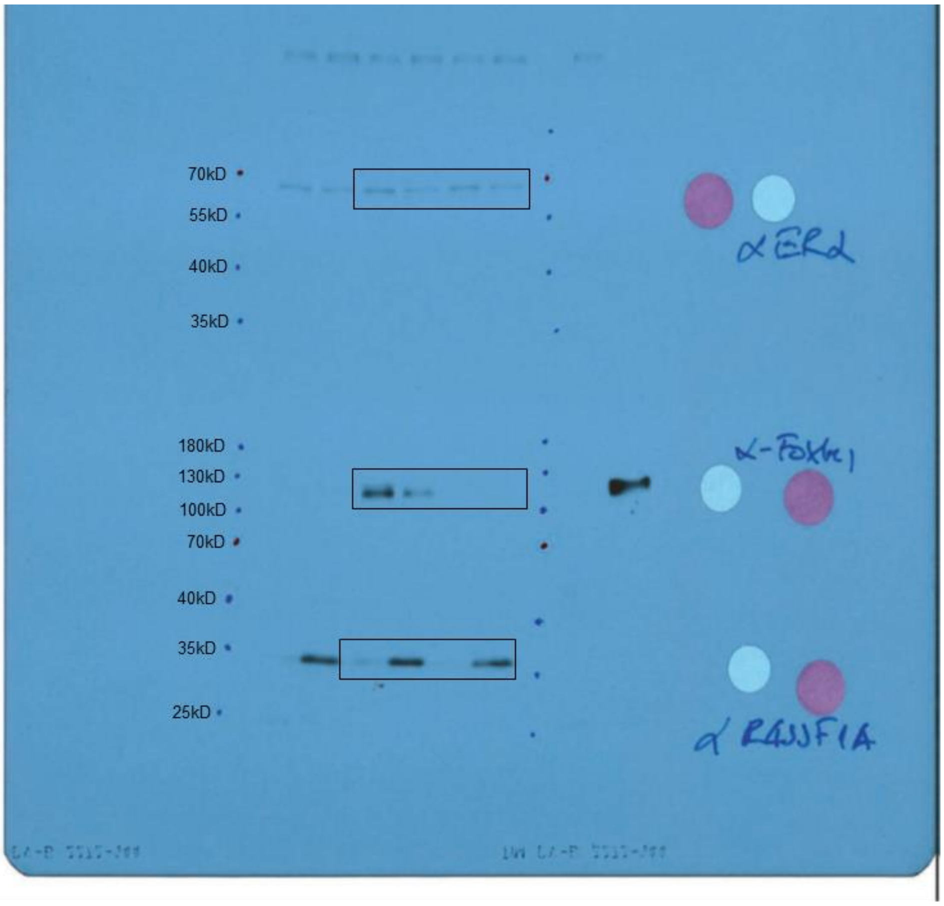

70kD
55kD
40kD
35kD
180kD
130kD
100kD
70kD
40kD
35kD
25kD

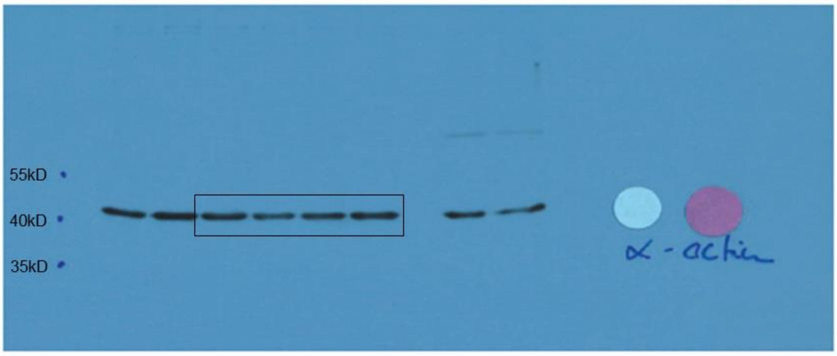
55kD
40kD
35kD
α - actin

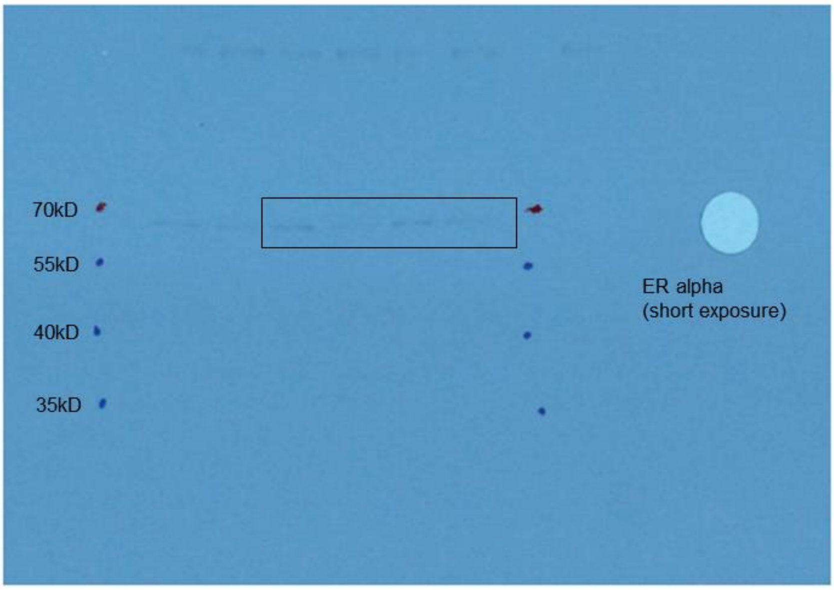
70kD
55kD
40kD
35kD
ER alpha
(short exposure)

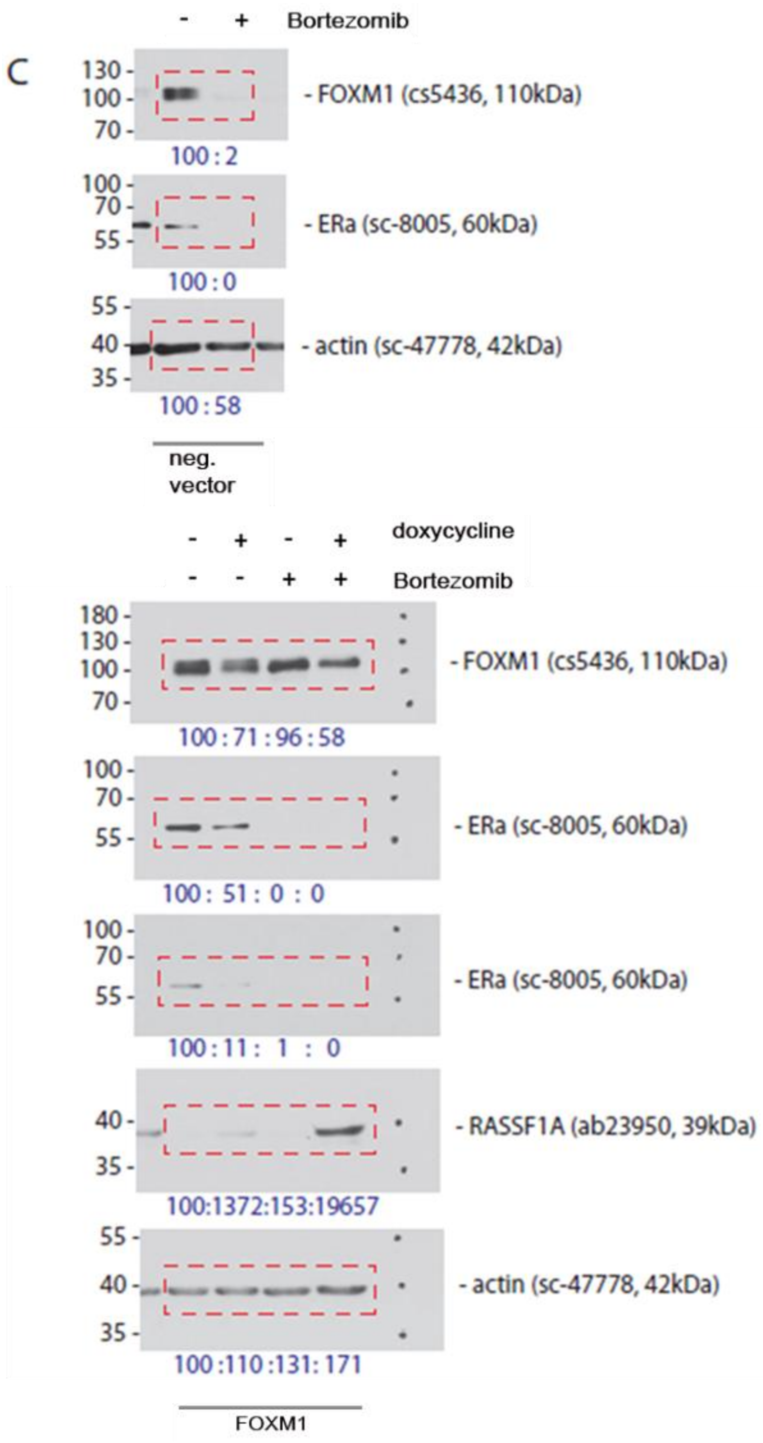
C
- + Bortezomib
130
100
70
- FOXM1 (cs5436, 110kDa)
100 : 2
100
70
55
- ERa (sc-8005, 60kDa)
100 : 0
55
40
35
- actin (sc-47778, 42kDa)
100 : 58
neg. vector
- + - + doxycycline
- - + + Bortezomib
180
130
100
70
- FOXM1 (cs5436, 110kDa)
100 : 71 : 96 : 58
100
70
55
- ERa (sc-8005, 60kDa)
100 : 51 : 0 : 0
100
70
55
- ERa (sc-8005, 60kDa)
100 : 11 : 1 : 0
40
35
- RASSF1A (ab23950, 39kDa)
100:1372:153:19657
55
40
35
- actin (sc-47778, 42kDa)
100 :110 :131: 171
FOXM1

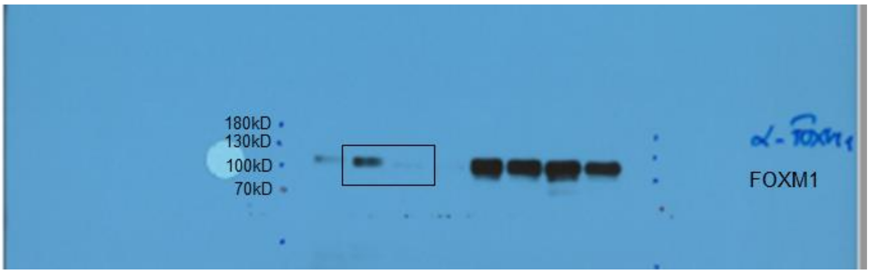
180kD
130kD
100kD
70kD
FOXM1

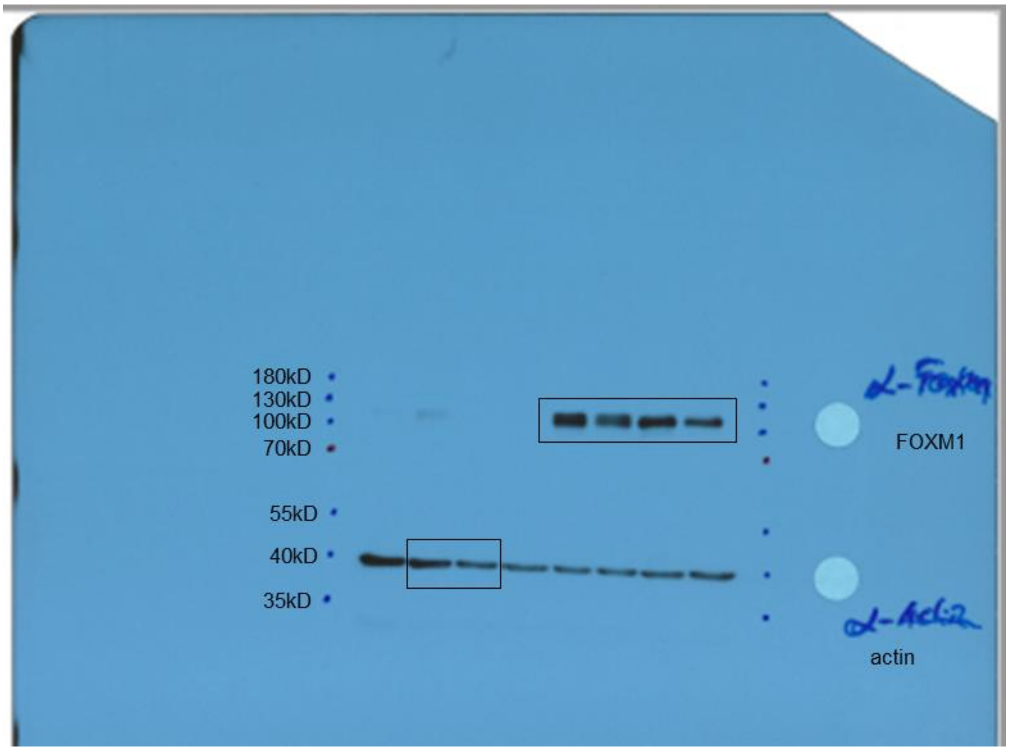
180kD
130kD
100kD
70kD
55kD
40kD
35kD
FOXM1
actin

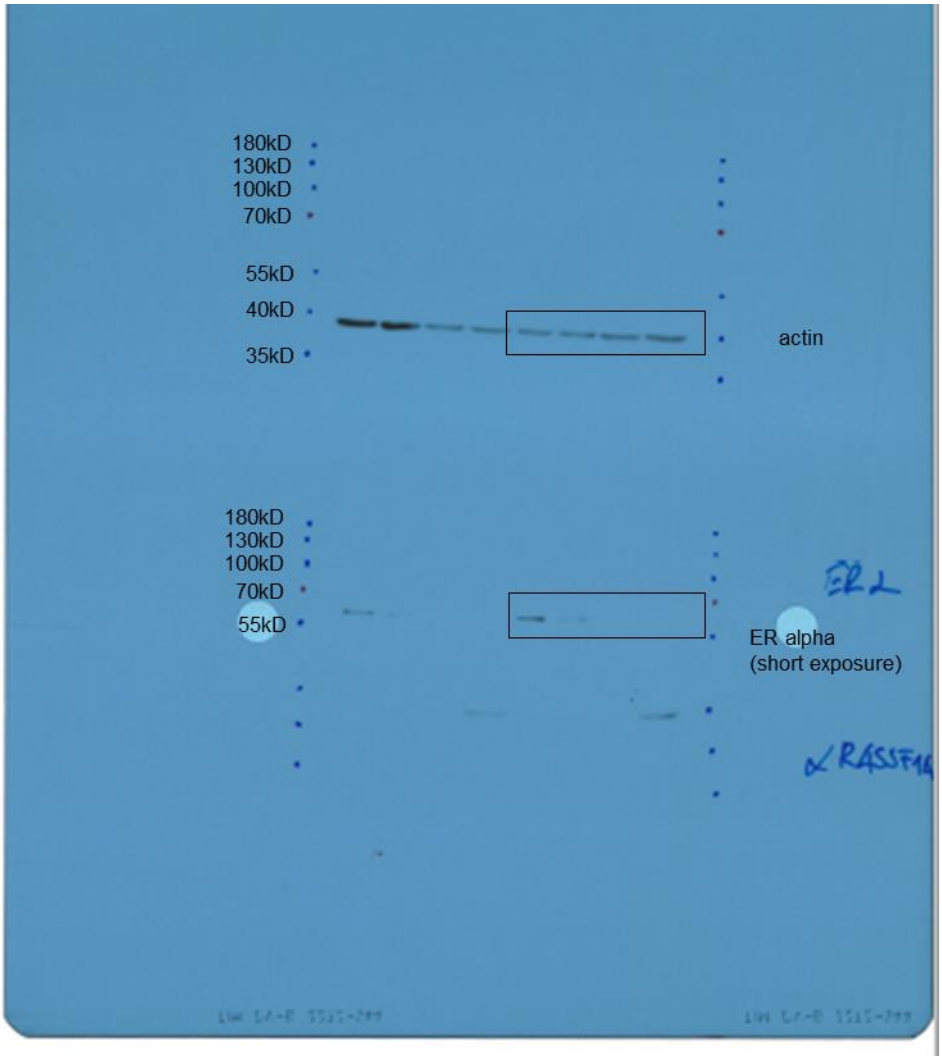
180kD
130kD
100kD
70kD
55kD
40kD
35kD
actin
180kD
130kD
100kD
70kD
55kD
ER alpha
(short exposure)

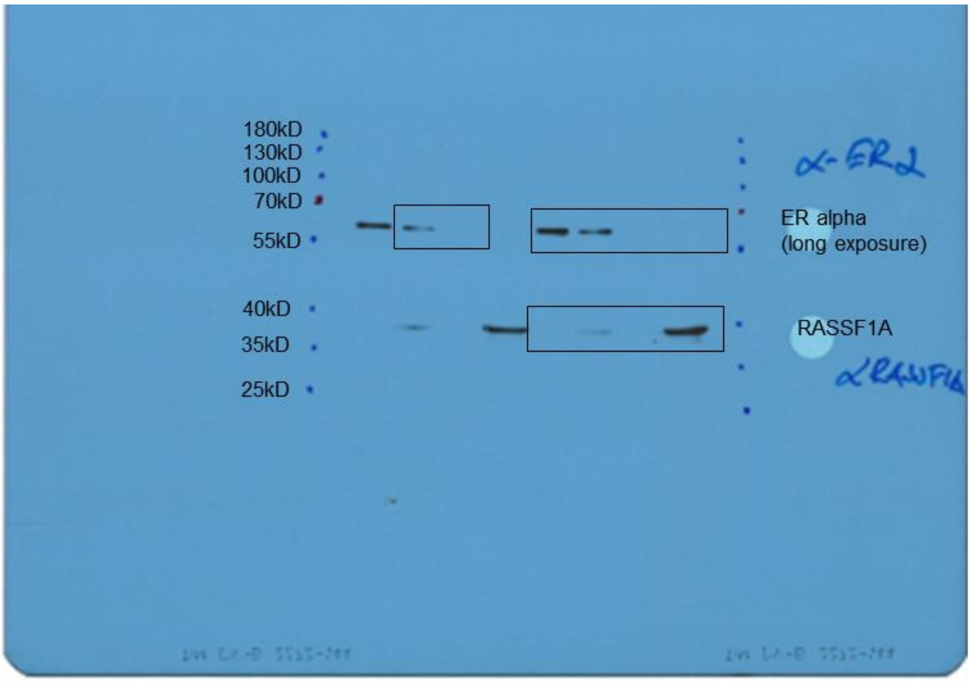

Figure S3. Ectopic overexpression of FOXM1 cannot rescue cells from RASSF1A-mediated ERα suppression and cell cycle arrest.

B

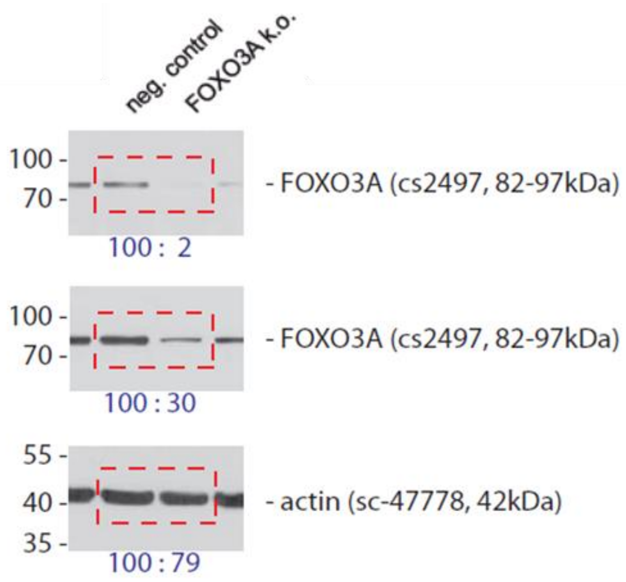
neg. control
FOXO3A k.o.
100 -
70 -
- FOXO3A (cs2497, 82-97kDa)
100 : 2
100 -
70 -
- FOXO3A (cs2497, 82-97kDa)
100 : 30
55 -
40 -
35 -
- actin (sc-47778, 42kDa)
100 : 79

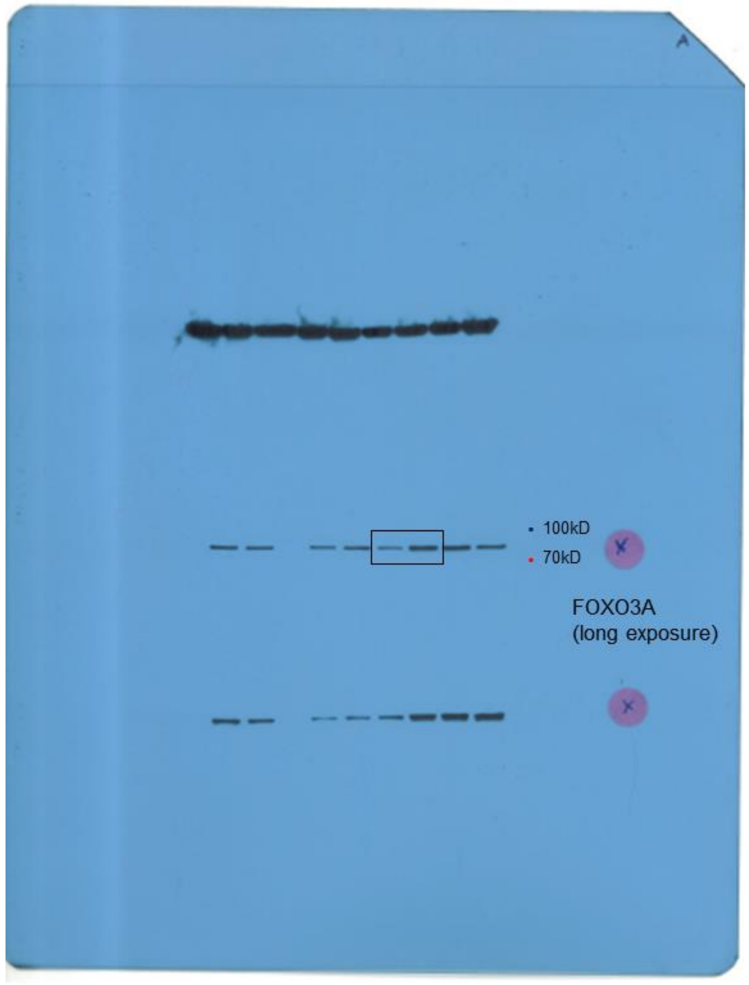
100kD
70kD
FOXO3A
(long exposure)

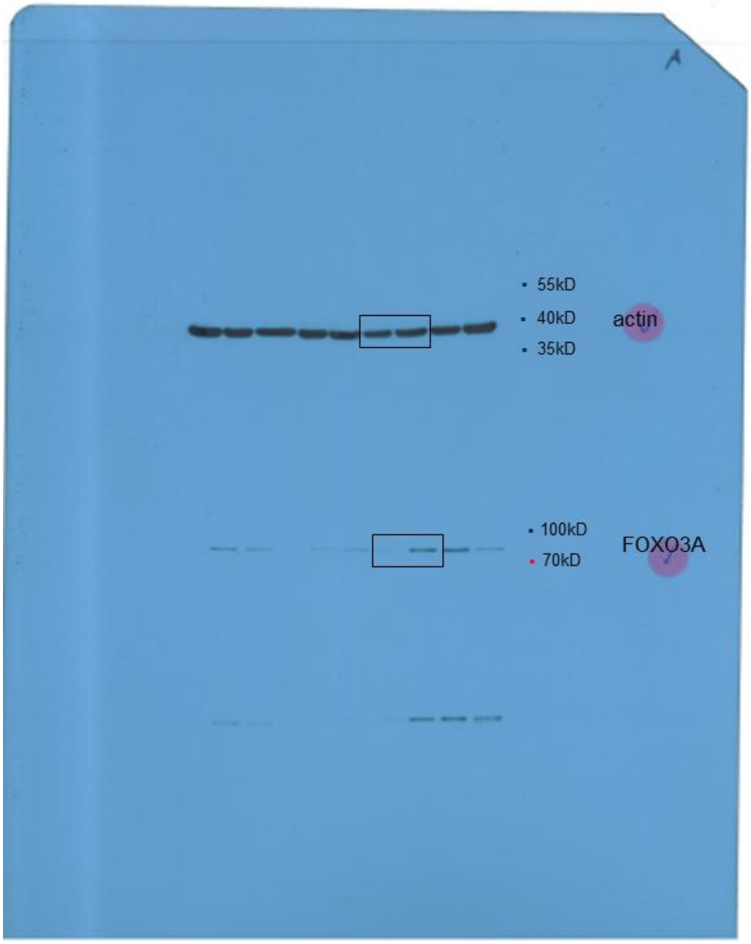
55kD
40kD
35kD
actin
100kD
70kD
FOXO3A

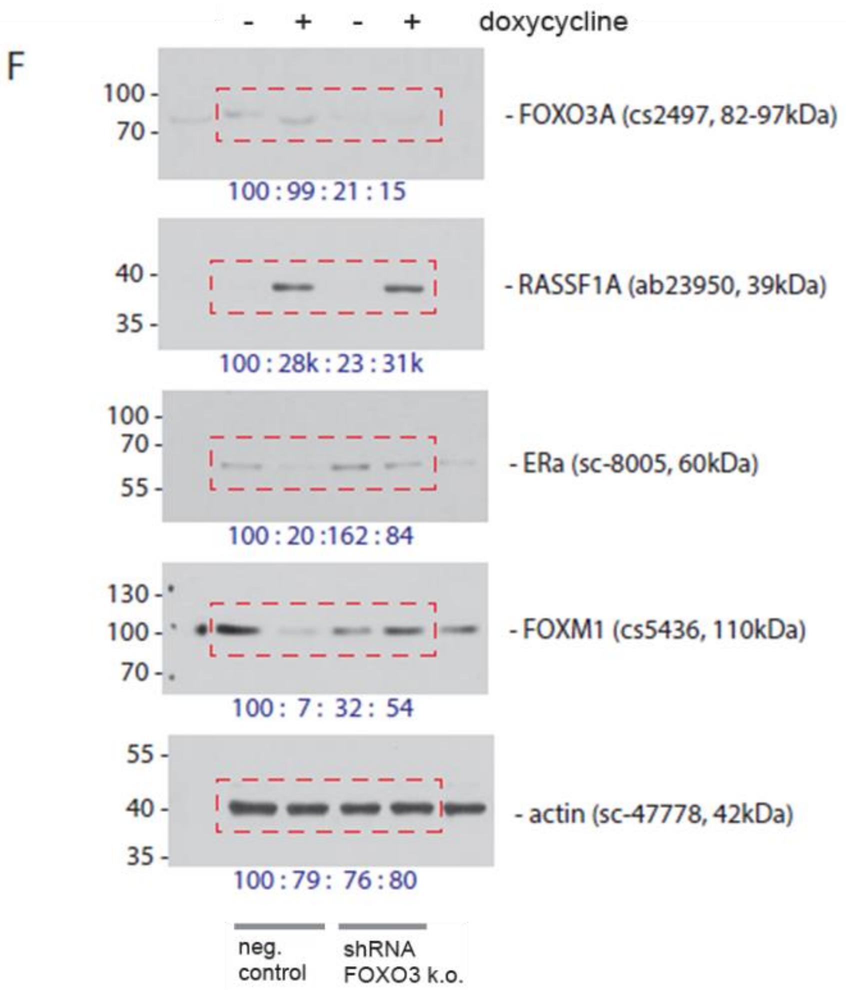

F
- + - + doxycycline
100 -
70 -
- FOXO3A (cs2497, 82-97kDa)
100 : 99 : 21 : 15
40 -
35 -
- RASSF1A (ab23950, 39kDa)
100 : 28k : 23 : 31k
100 -
70 -
55 -
- ERa (sc-8005, 60kDa)
100 : 20 :162 : 84
130 -
100 -
70 -
- FOXM1 (cs5436, 110kDa)
100 : 7 : 32 : 54
55 -
40 -
35 -
- actin (sc-47778, 42kDa)
100 : 79 : 76 : 80
neg. control
shRNA FOXO3 k.o.

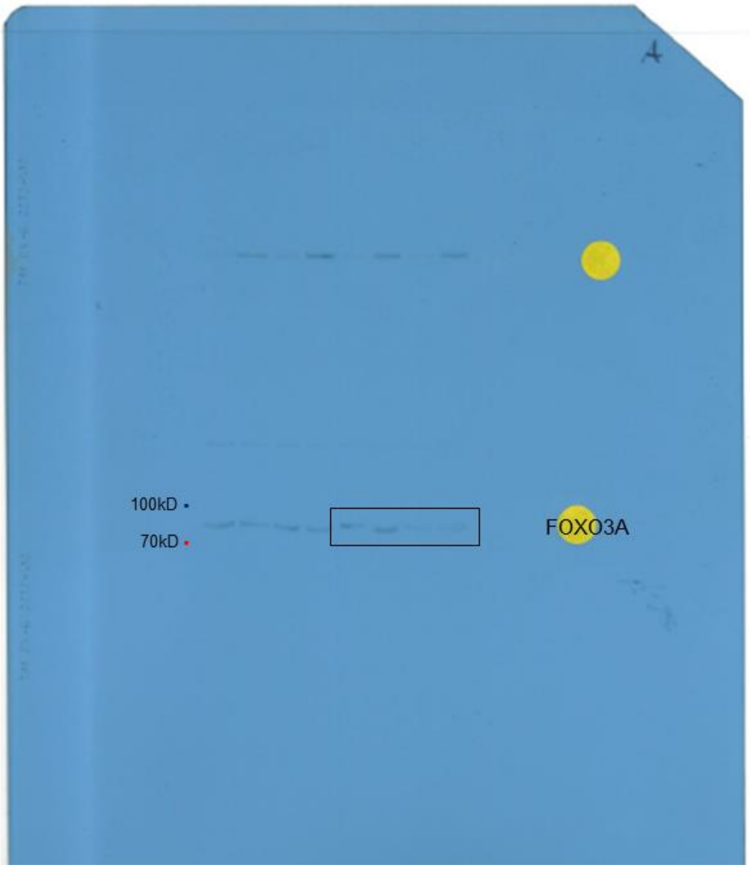

100kD
70kD
FOXO3A

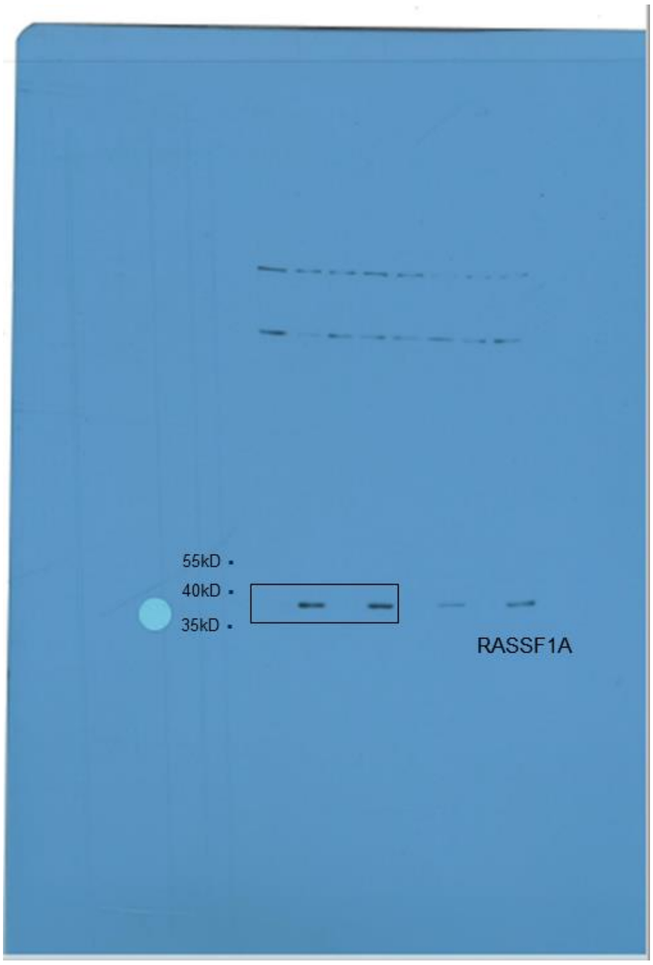
55kD
40kD
35kD
RASSF1A

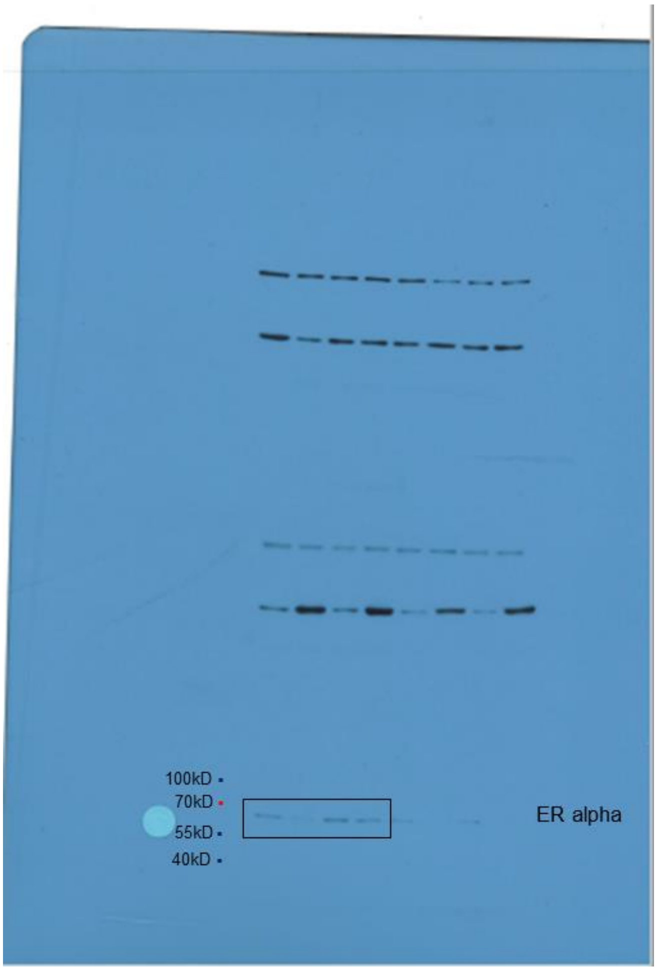
100kD
70kD
55kD
40kD
ER alpha

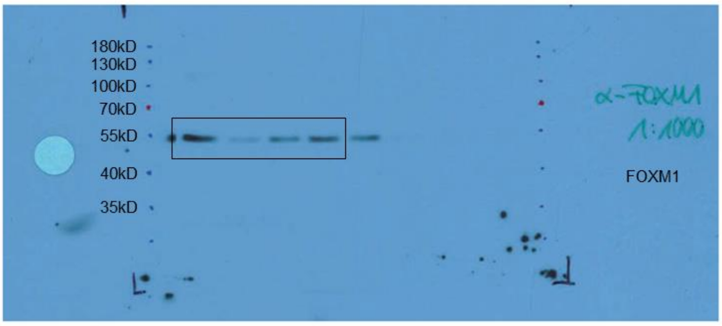
180kD
130kD
100kD
70kD
55kD
40kD
35kD
α-FOXM1
1:1000
FOXM1

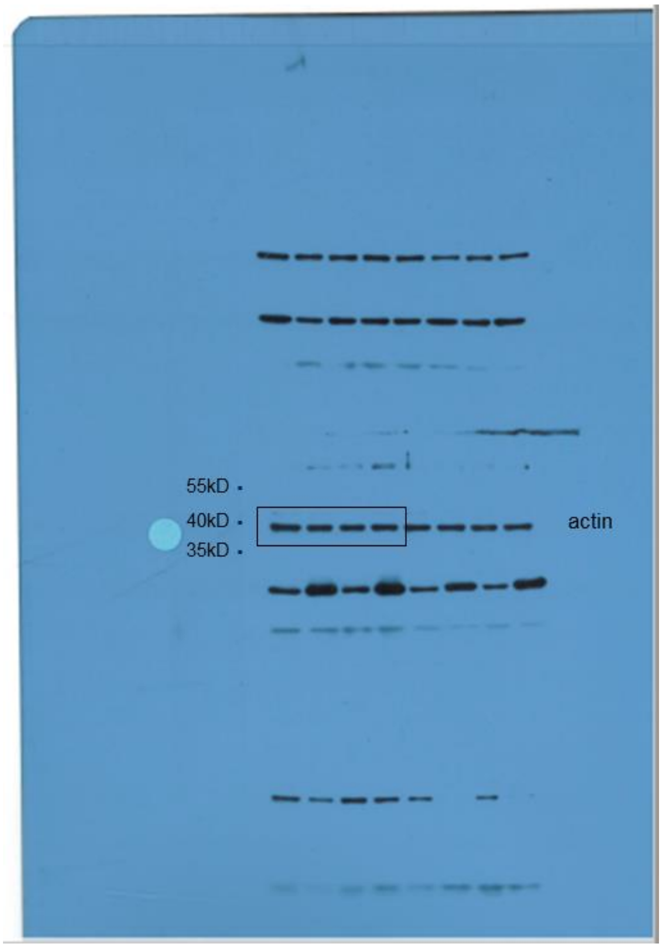
55kD
40kD
35kD
actin

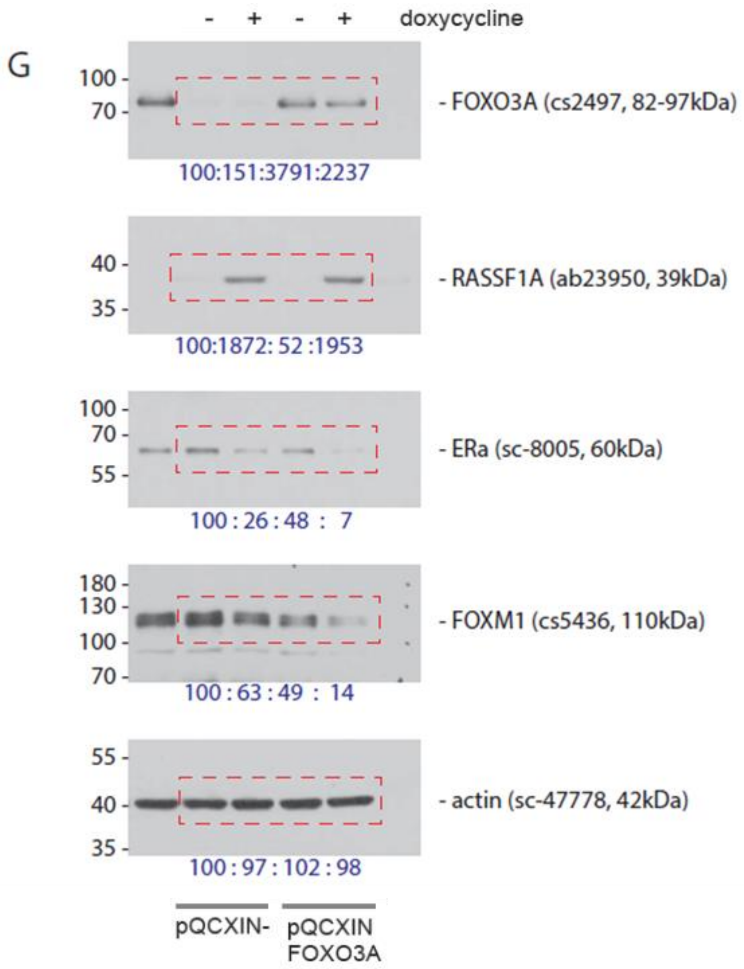
- + - + doxycycline
G
100
70
- FOXO3A (cs2497, 82-97kDa)
100:151:3791:2237
40
35
- RASSF1A (ab23950, 39kDa)
100:1872: 52 :1953
100
70
55
- ERa (sc-8005, 60kDa)
100 : 26 : 48 : 7
180
130
100
70
- FOXM1 (cs5436, 110kDa)
100 : 63 : 49 : 14
55
40
35
- actin (sc-47778, 42kDa)
100 : 97 : 102 : 98
pQCXIN-
pQCXIN FOXO3A

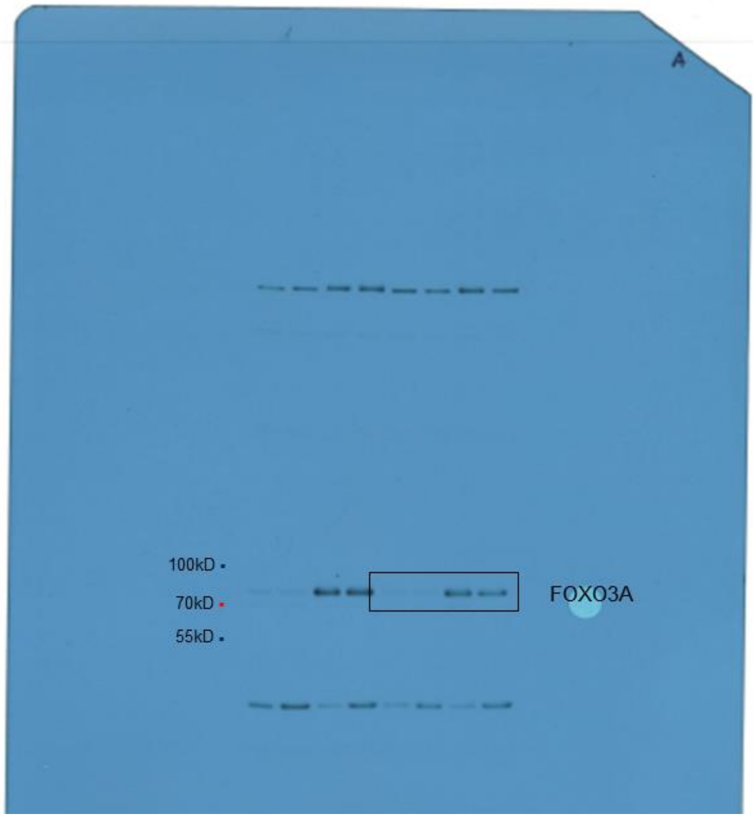
100kD
70kD
55kD
FOXO3A

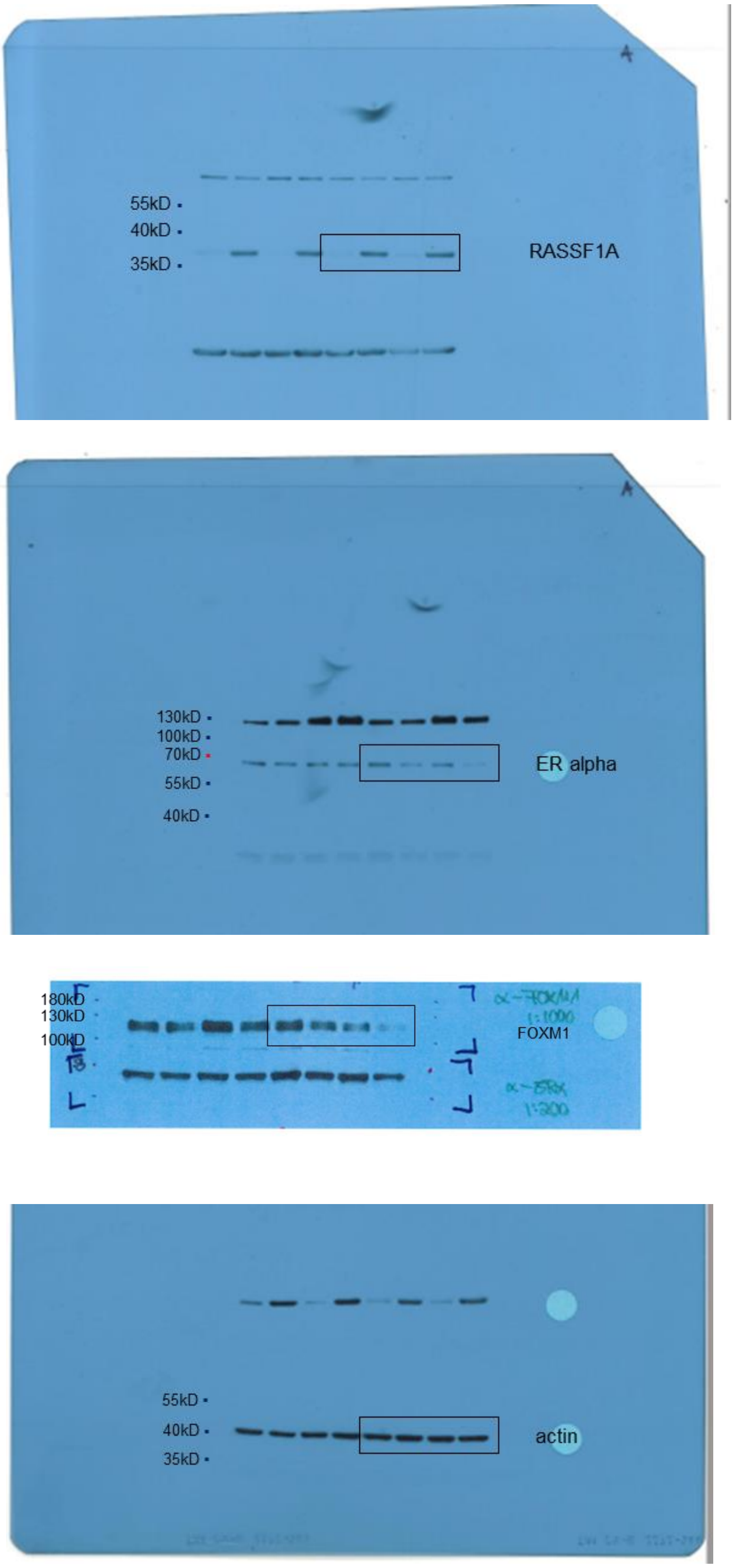

Figure S4. FOXO3A is required for RASSF1A-mediated growth arrest.

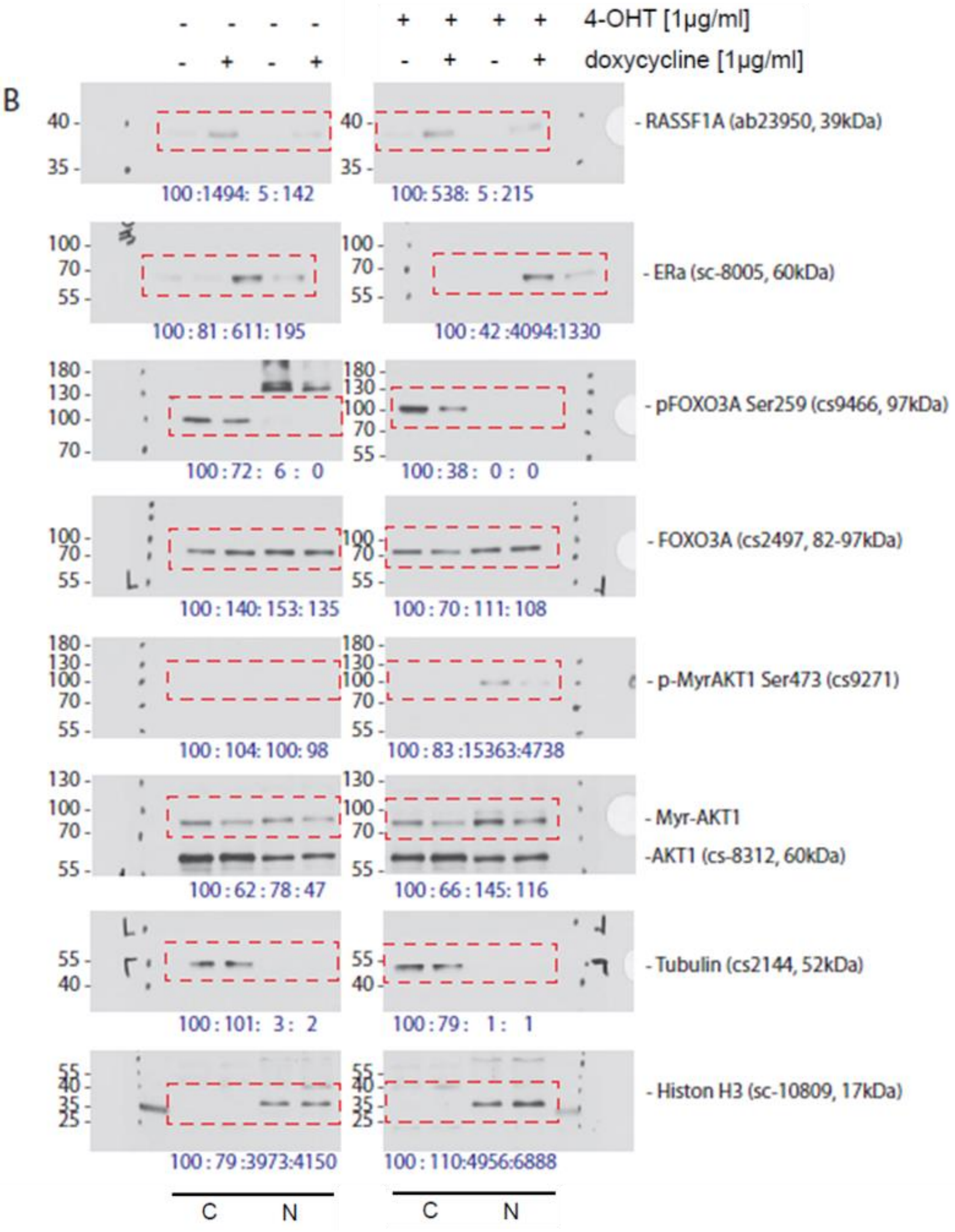

4-OHT [1µg/ml]
doxycycline [1µg/ml]
B
- RASSF1A (ab23950, 39kDa)
100 :1494: 5 : 142
100: 538: 5 : 215
- ERa (sc-8005, 60kDa)
100 : 81 : 611: 195
100 : 42 :4094:1330
- pFOXO3A Ser259 (cs9466, 97kDa)
100 : 72 : 6 : 0
100 : 38 : 0 : 0
- FOXO3A (cs2497, 82-97kDa)
100 : 140: 153: 135
100 : 70 : 111: 108
- p-MyrAKT1 Ser473 (cs9271)
100 : 104: 100: 98
100 : 83 :15363:4738
- Myr-AKT1
-AKT1 (cs-8312, 60kDa)
100 : 62 : 78 : 47
100 : 66 : 145: 116
- Tubulin (cs2144, 52kDa)
100 : 101: 3 : 2
100 : 79 : 1 : 1
- Histon H3 (sc-10809, 17kDa)
100 : 79 :3973:4150
100 : 110:4956:6888
C
N
C
N

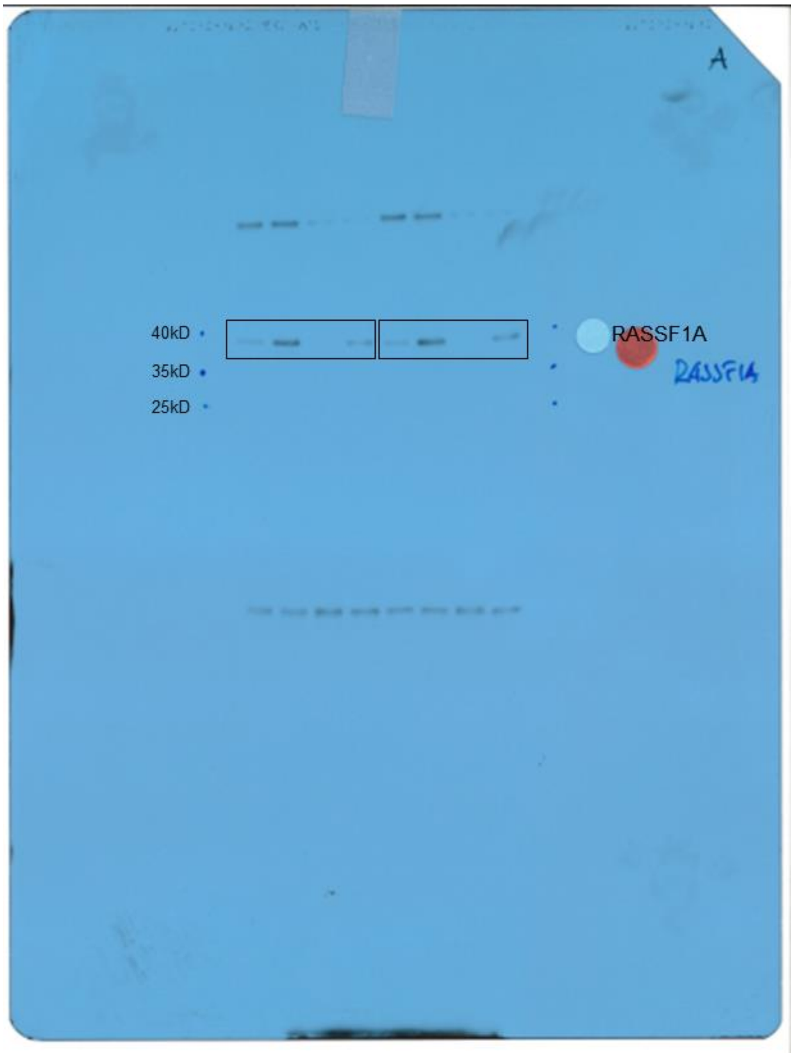
A
40kD
35kD
25kD
RASSF1A
RASSF1A

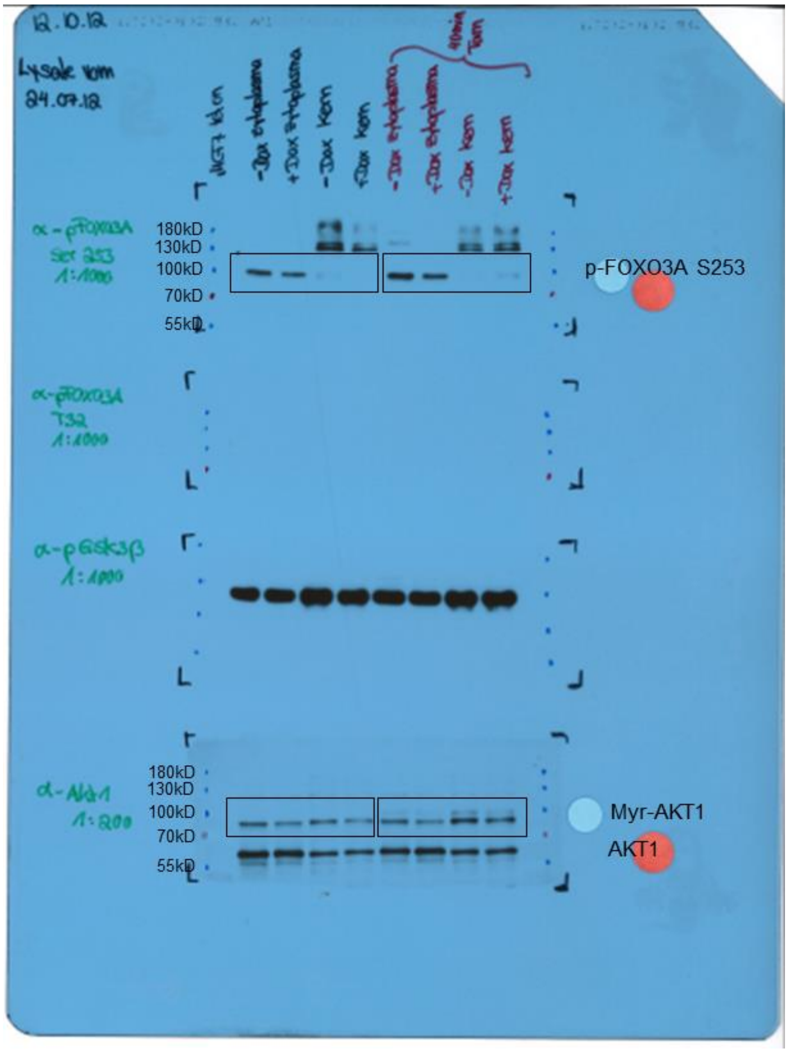

180kD
130kD
100kD
70kD
55kD
p-FOXO3A S253
180kD
130kD
100kD
70kD
55kD
Myr-AKT1
AKT1

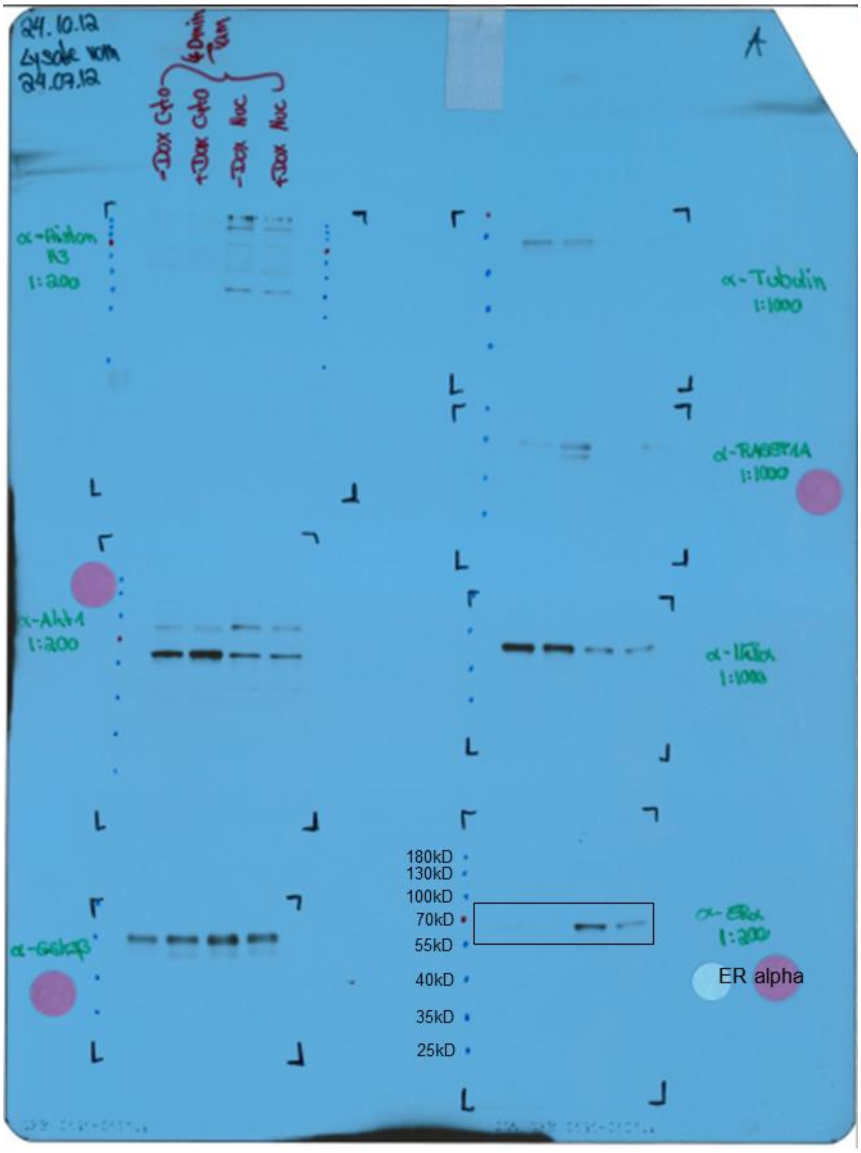
24.10.12
α-Tubulin
1:1000
180kD
130kD
100kD
70kD
55kD
40kD
35kD
25kD
ER alpha

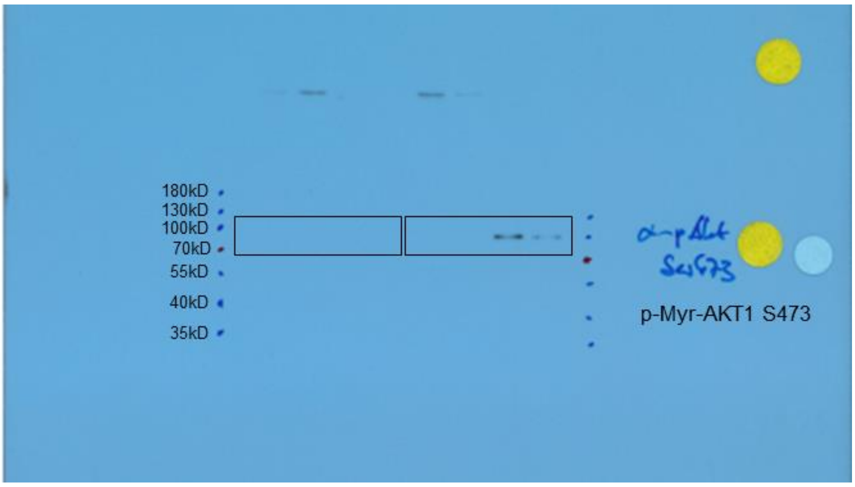
180kD
130kD
100kD
70kD
55kD
40kD
35kD
p-Myr-AKT1 S473

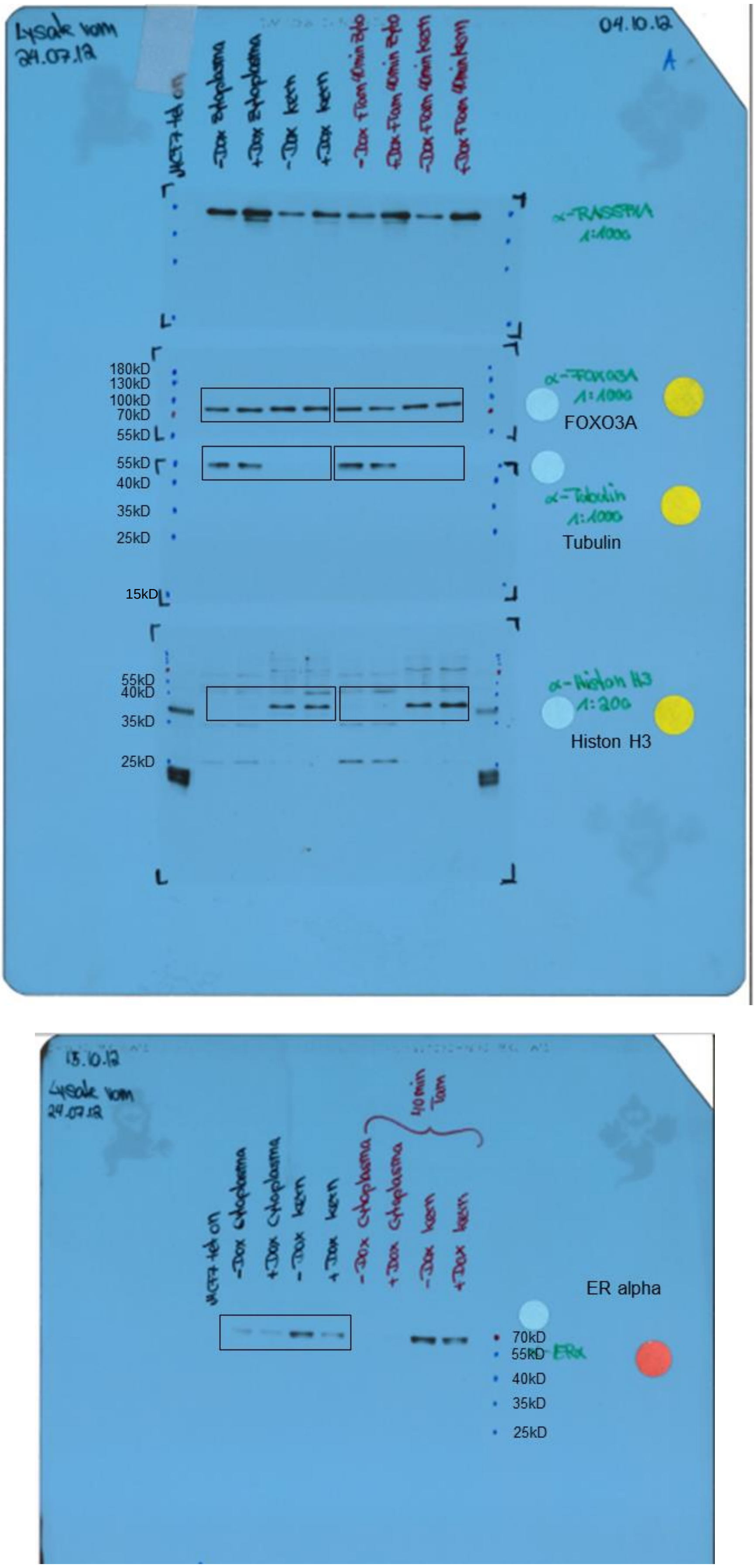

Figure S5. RASSF1A increases FOXO3A transcriptional activity through suppression of AKT-mediated inhibitory phosphorylation and through increased expression of FOXO3A.

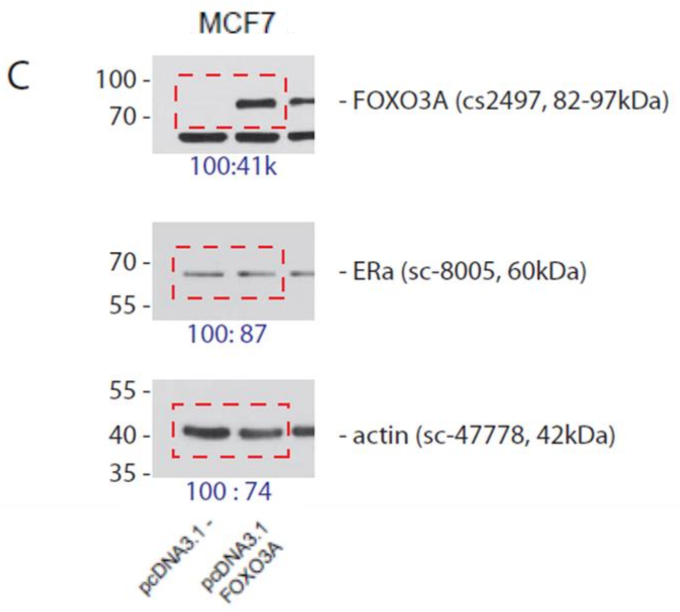
MCF7
C
100 -
70 -
- FOXO3A (cs2497, 82-97kDa)
100:41k
70 -
55 -
- ERa (sc-8005, 60kDa)
100: 87
55 -
40 -
35 -
- actin (sc-47778, 42kDa)
100 : 74
pcDNA3.1 -
pcDNA3.1 FOXO3A

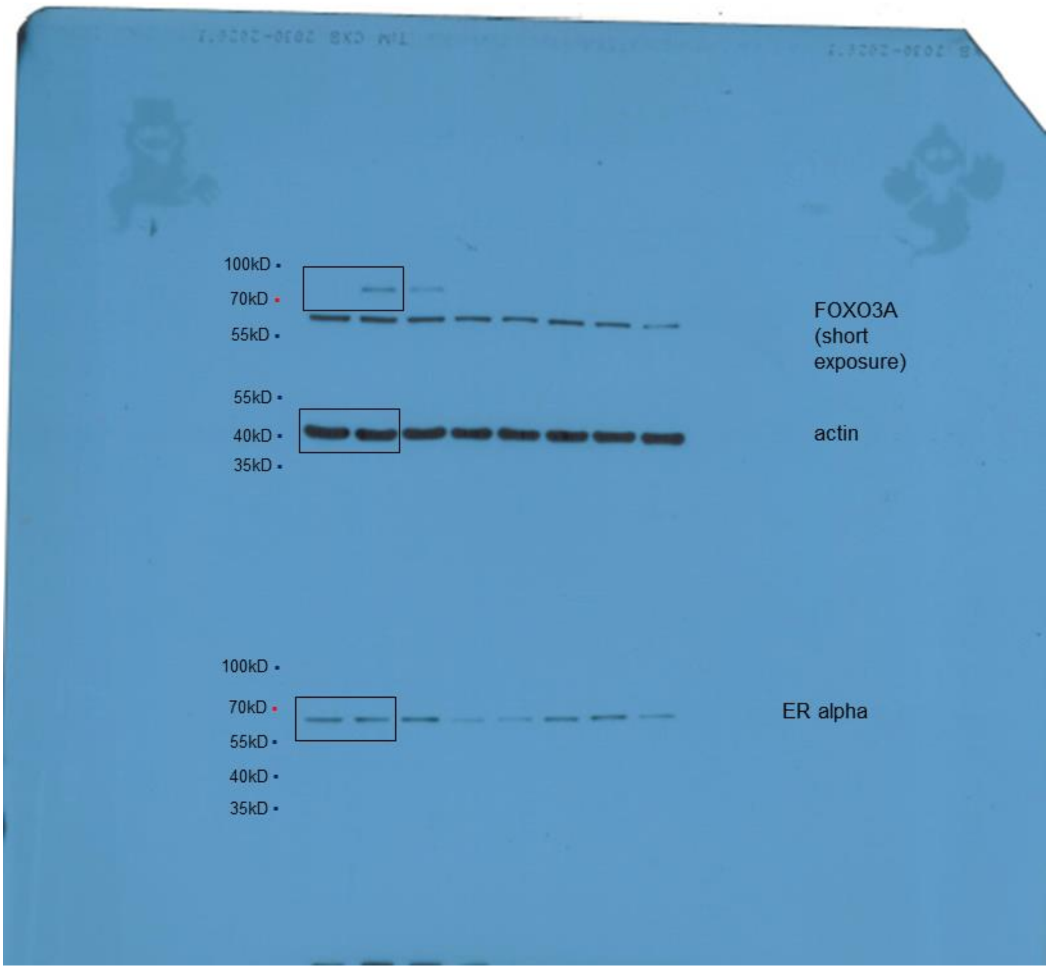
100kD
70kD
55kD
FOXO3A (short exposure)
55kD
40kD
35kD
actin
100kD
70kD
55kD
40kD
35kD
ER alpha

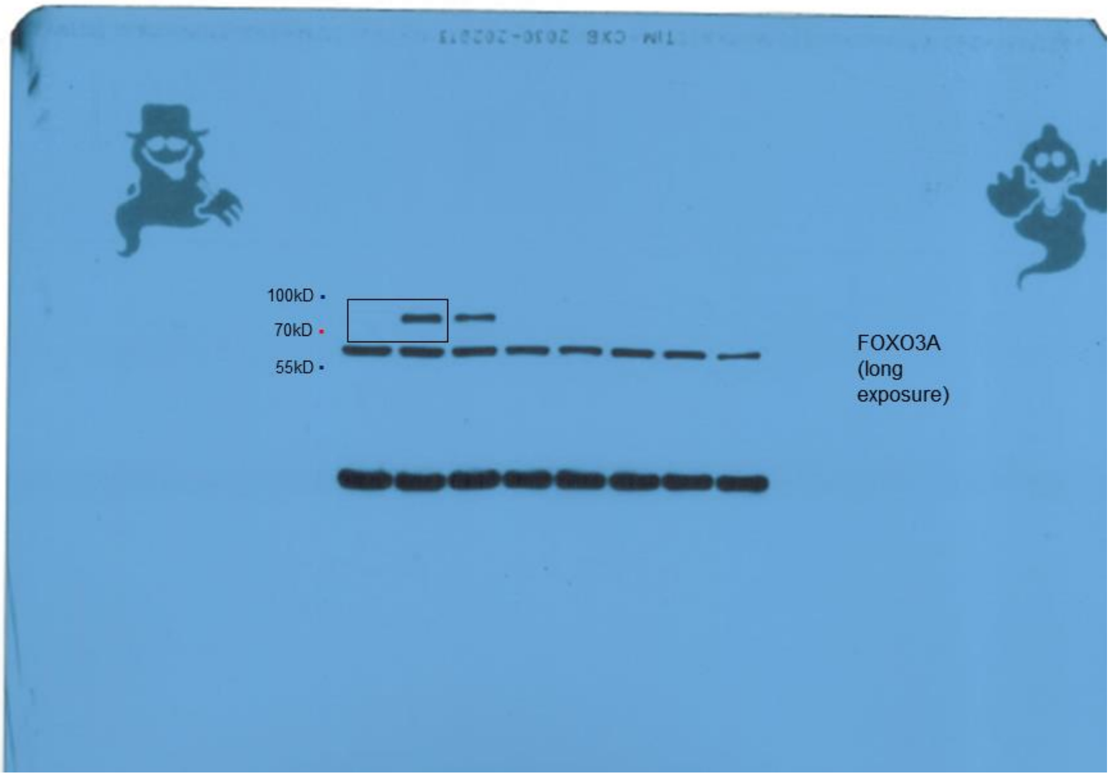

Figure S6. RASSF1A and FOXO3A cause decreased ERα and ERβ activity.

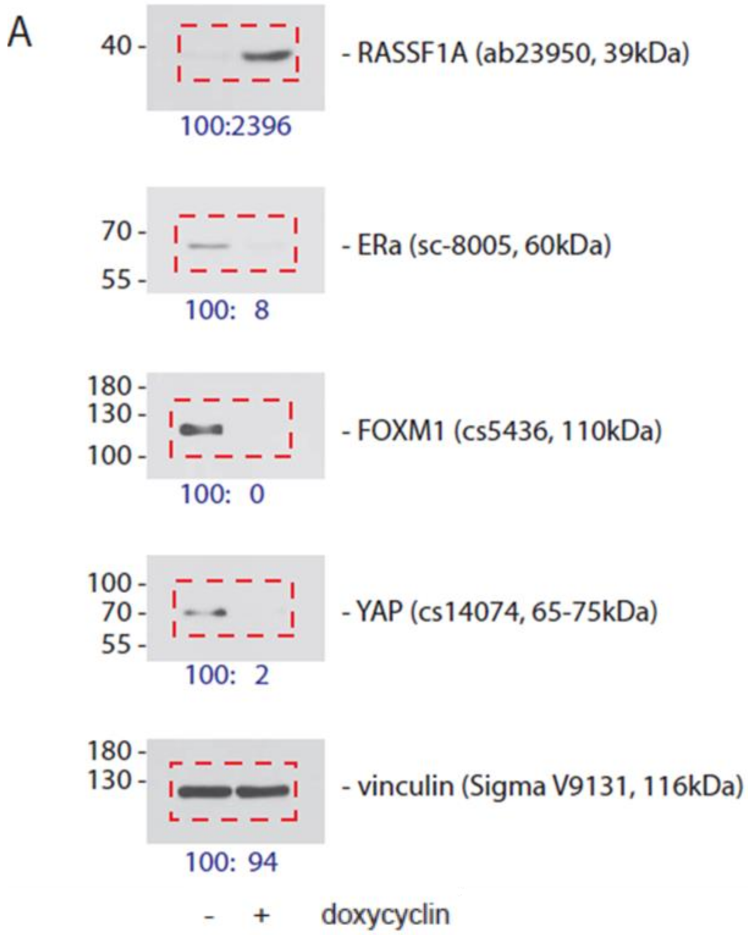
A
40 -
- RASSF1A (ab23950, 39kDa)
100:2396
70 -
55 -
- ERa (sc-8005, 60kDa)
100: 8
180 -
130 -
100 -
- FOXM1 (cs5436, 110kDa)
100: 0
100 -
70 -
55 -
- YAP (cs14074, 65-75kDa)
100: 2
180 -
130 -
- vinculin (Sigma V9131, 116kDa)
100: 94
- + doxycyclin

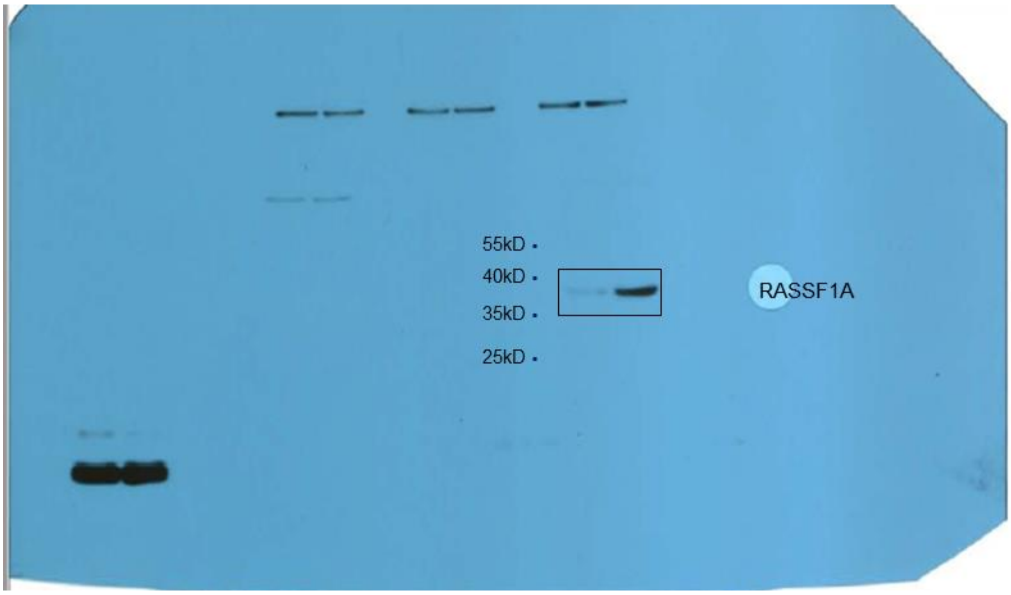
55kD
40kD
35kD
25kD
RASSF1A

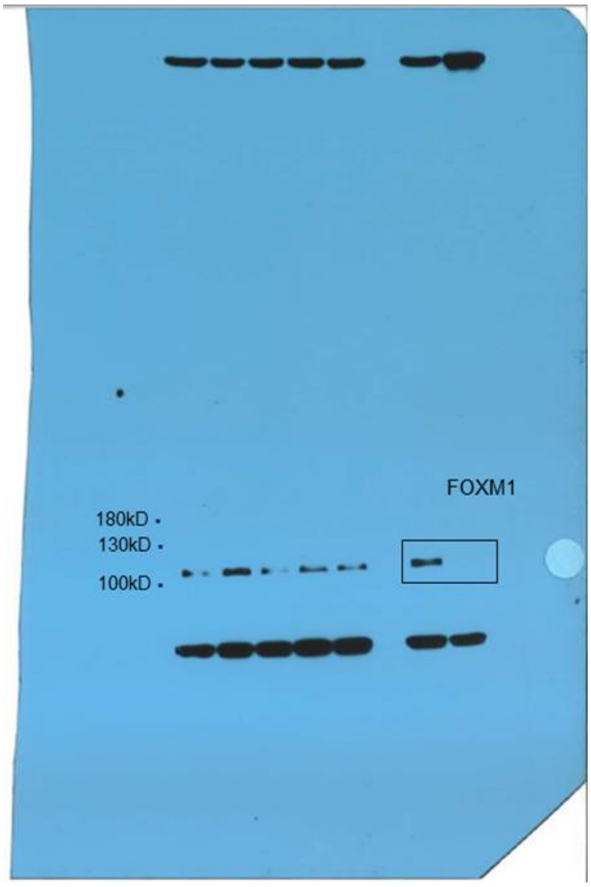
FOXM1
180kD
130kD
100kD

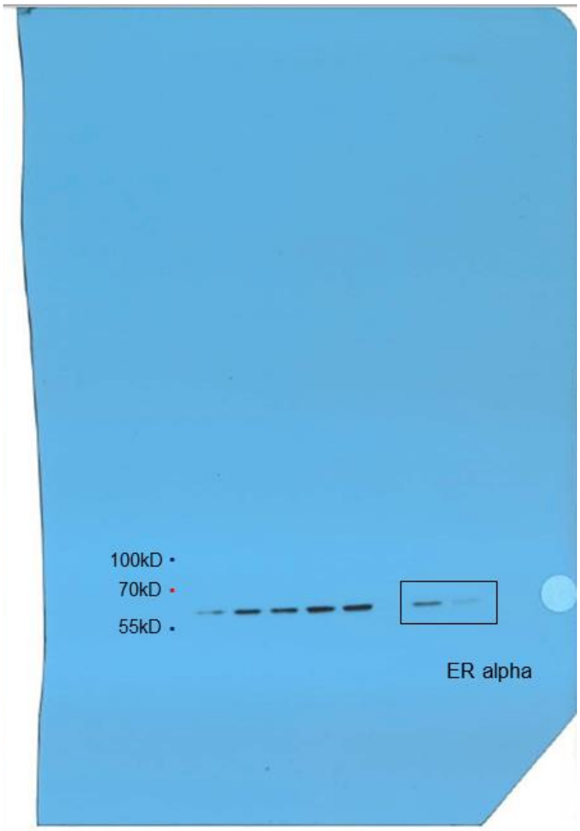
100kD
70kD
55kD
ER alpha

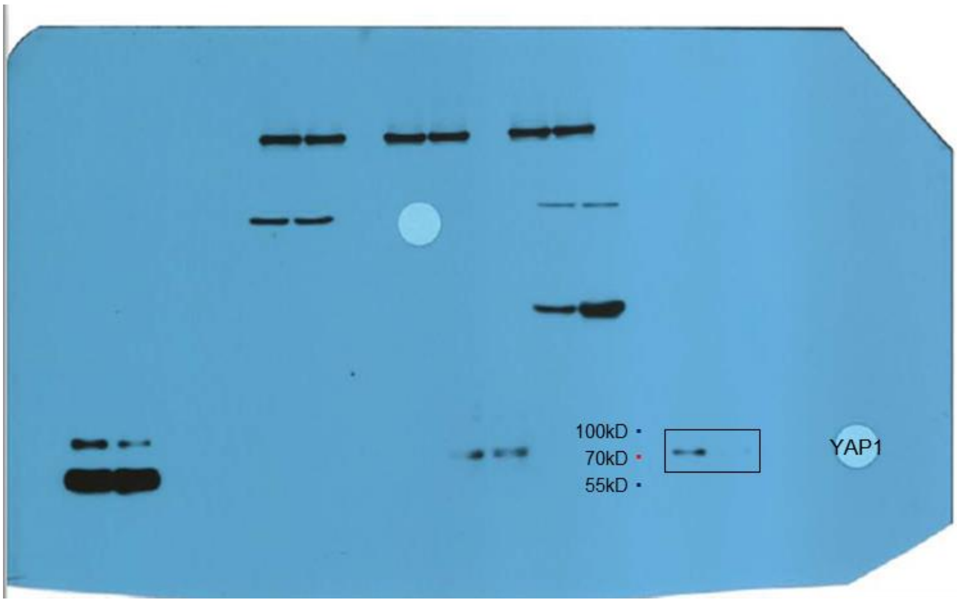
100kD
70kD
55kD
YAP1

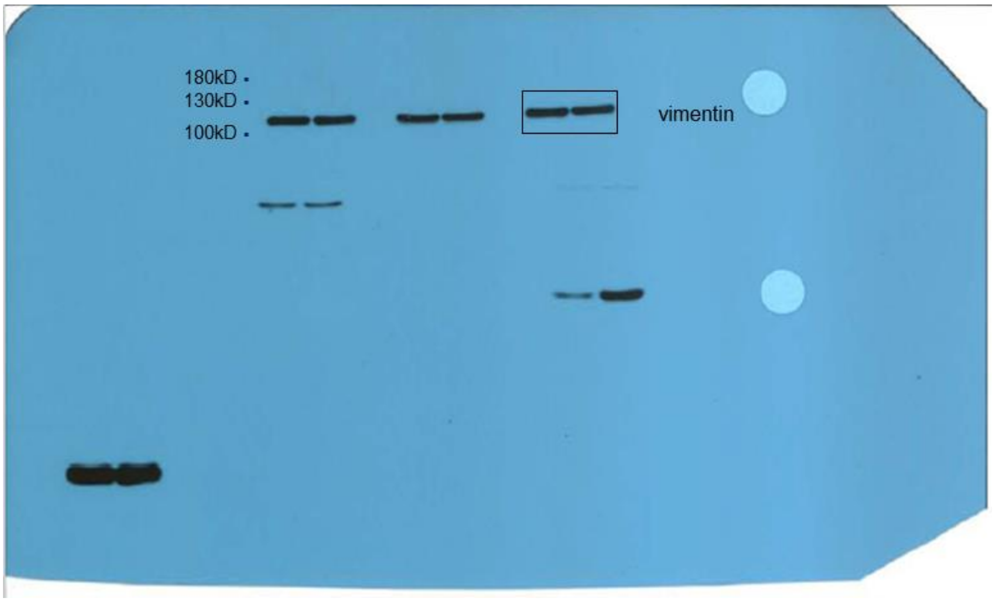
180kD
130kD
100kD
vimentin

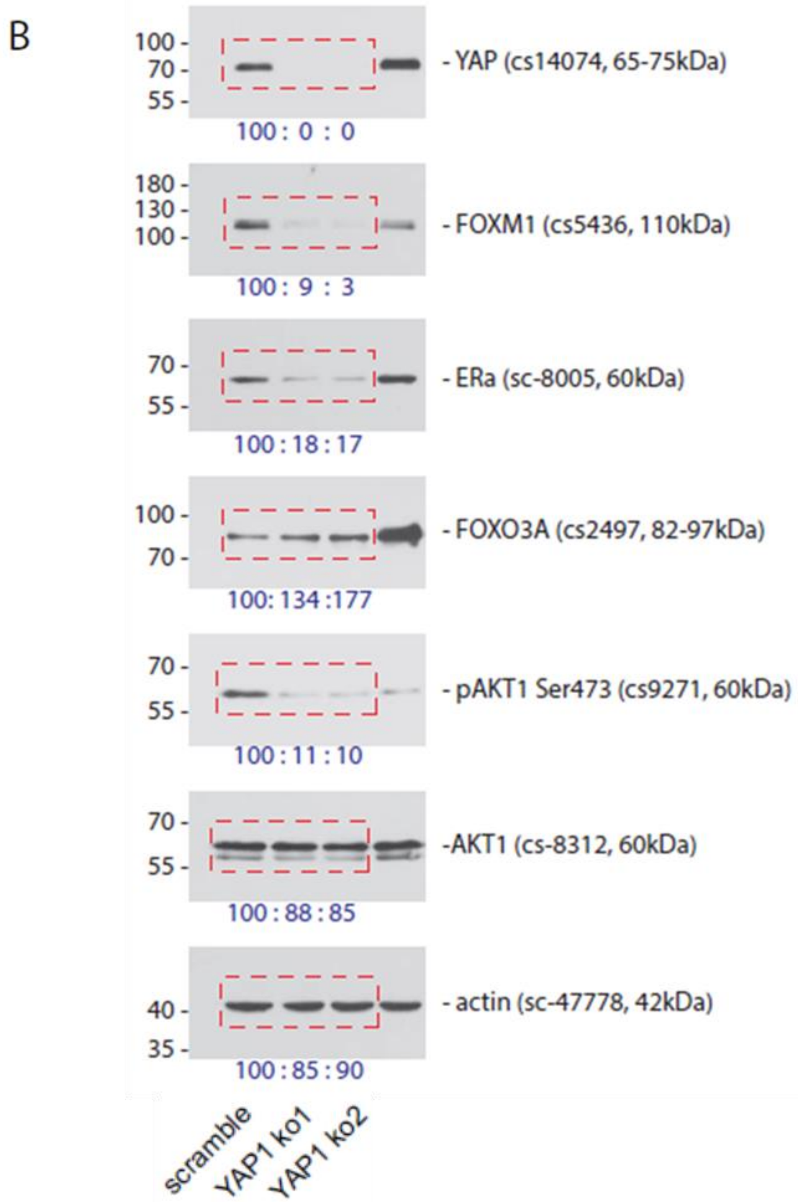
B
100 -
70 -
55 -
- YAP (cs14074, 65-75kDa)
100 : 0 : 0
180 -
130 -
100 -
- FOXM1 (cs5436, 110kDa)
100 : 9 : 3
70 -
55 -
- ERa (sc-8005, 60kDa)
100 : 18 : 17
100 -
70 -
- FOXO3A (cs2497, 82-97kDa)
100: 134 :177
70 -
55 -
- pAKT1 Ser473 (cs9271, 60kDa)
100 : 11 : 10
70 -
55 -
-AKT1 (cs-8312, 60kDa)
100 : 88 : 85
40 -
35 -
- actin (sc-47778, 42kDa)
100 : 85 : 90
scramble
YAP1 ko1
YAP1 ko2

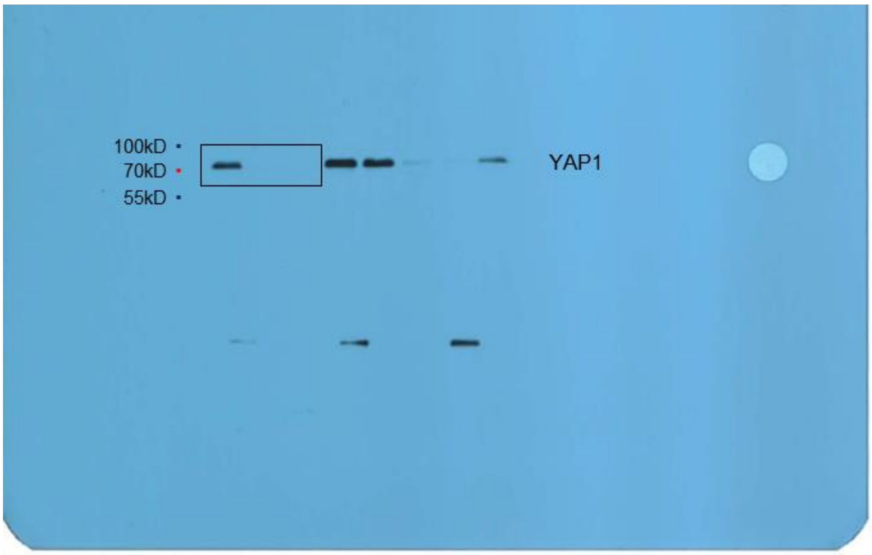
100kD
70kD
55kD
YAP1

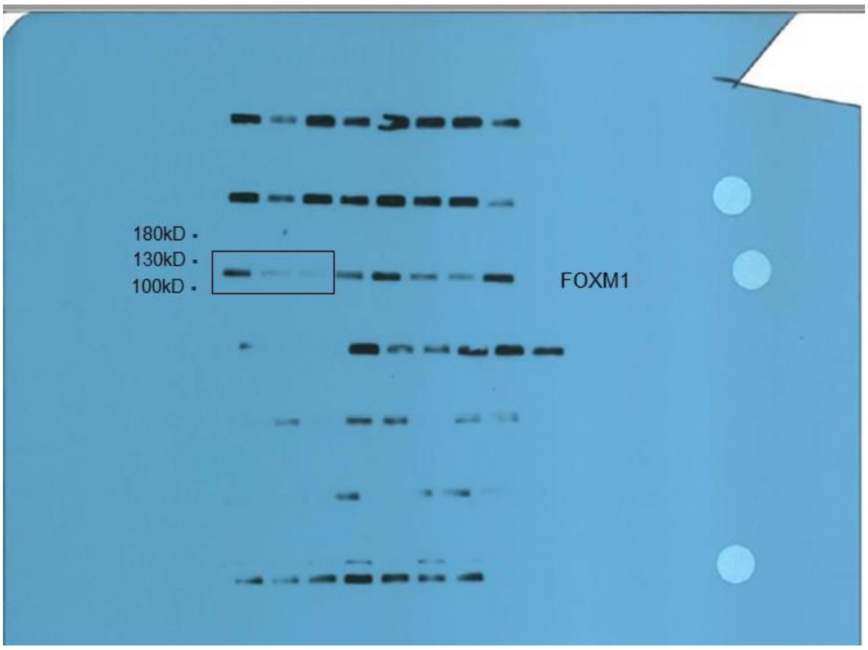
180kD
130kD
100kD
FOXM1

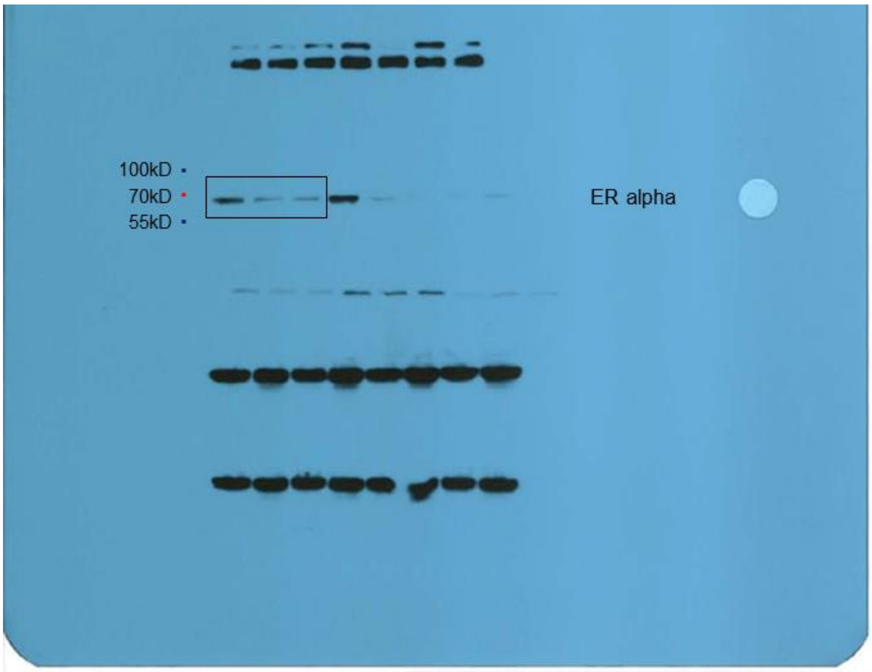
100kD
70kD
55kD
ER alpha

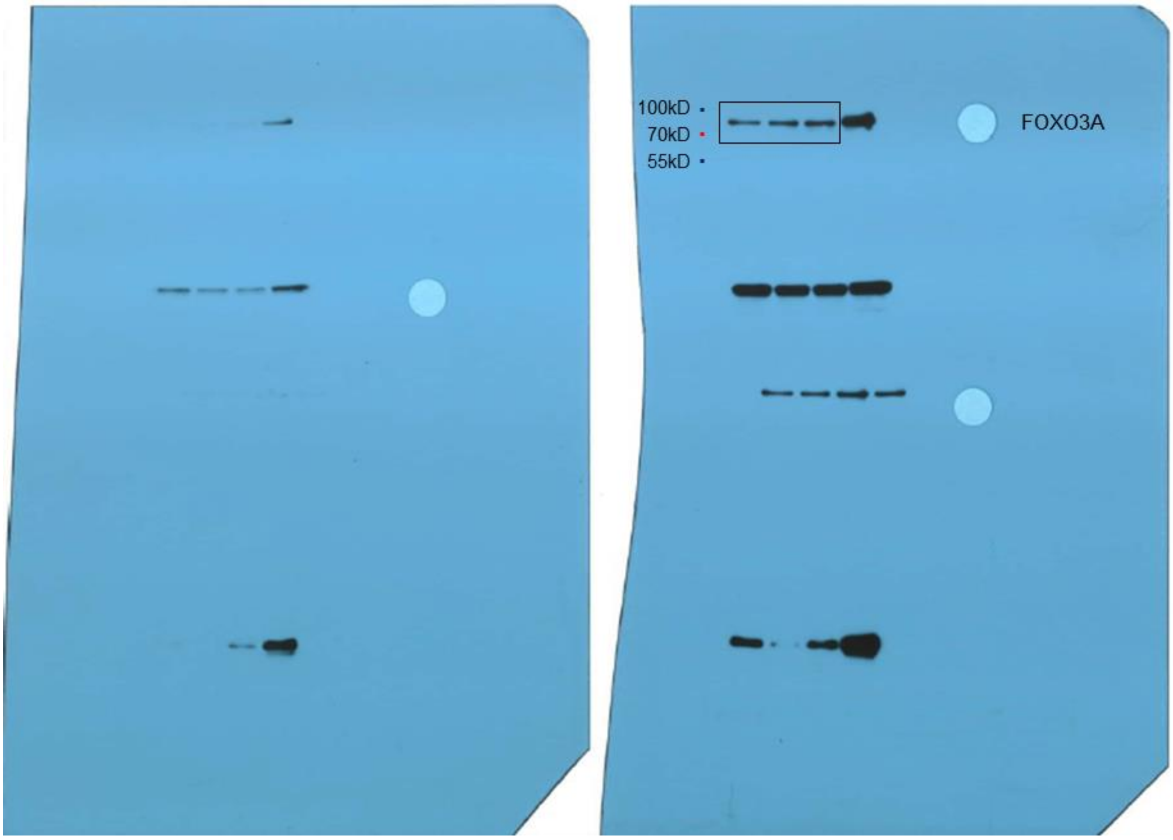
100kD
70kD
55kD
FOXO3A

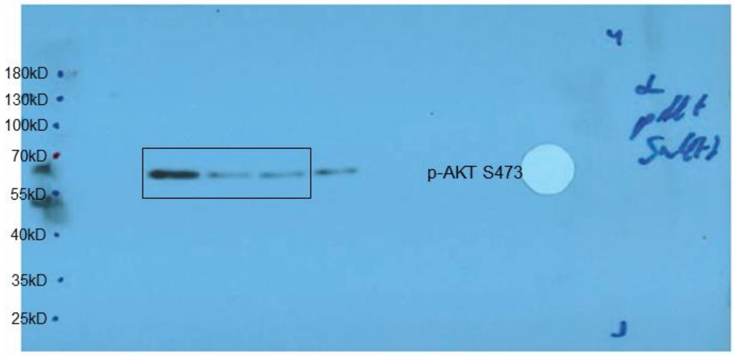
180kD
130kD
100kD
70kD
55kD
40kD
35kD
25kD
p-AKT S473

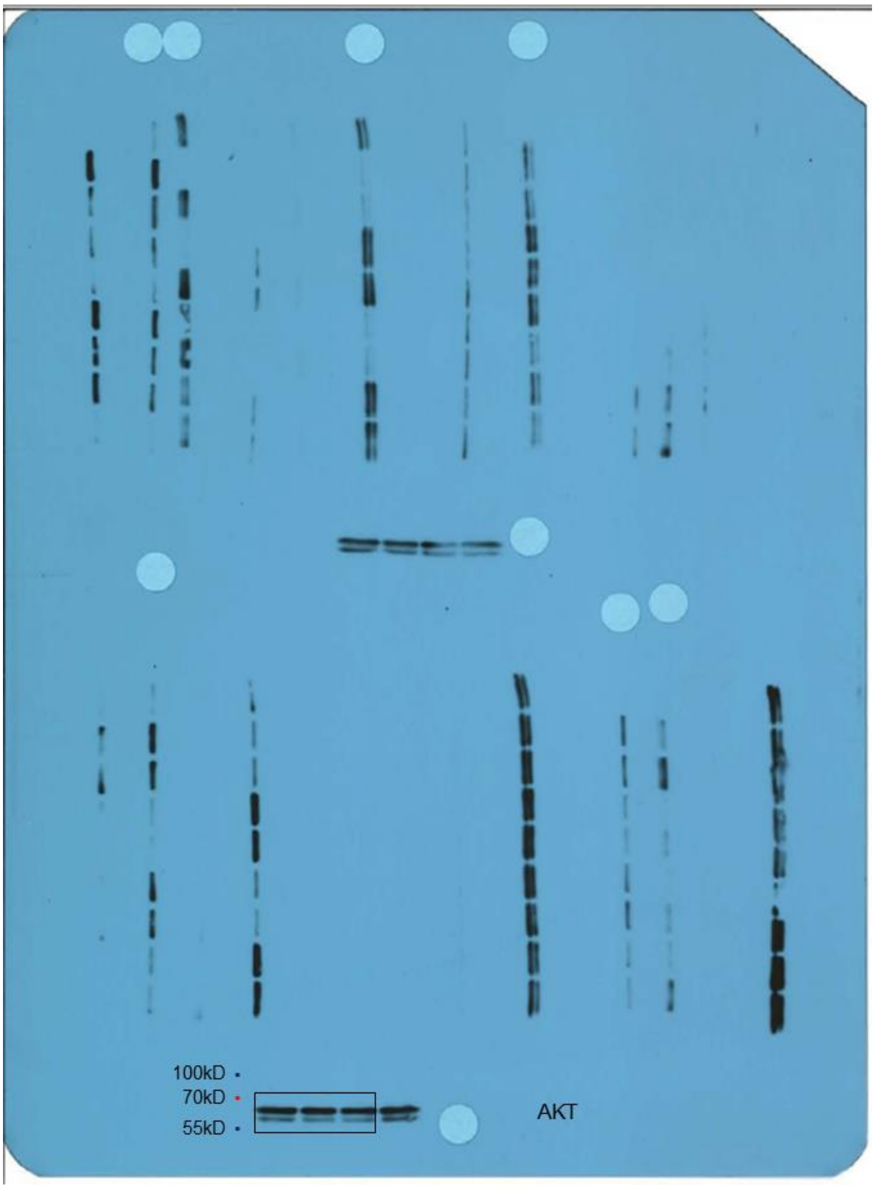
100kD
70kD
55kD
AKT

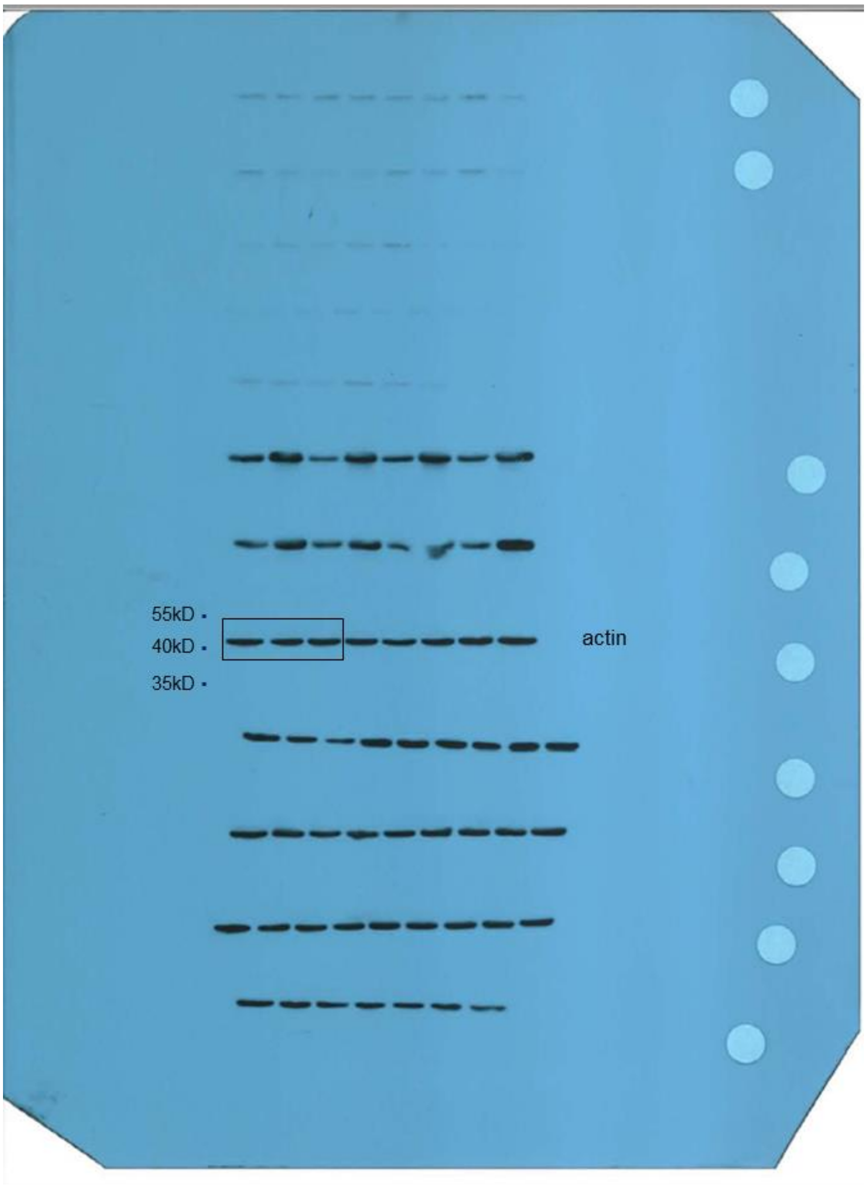
55kD
40kD
35kD
actin

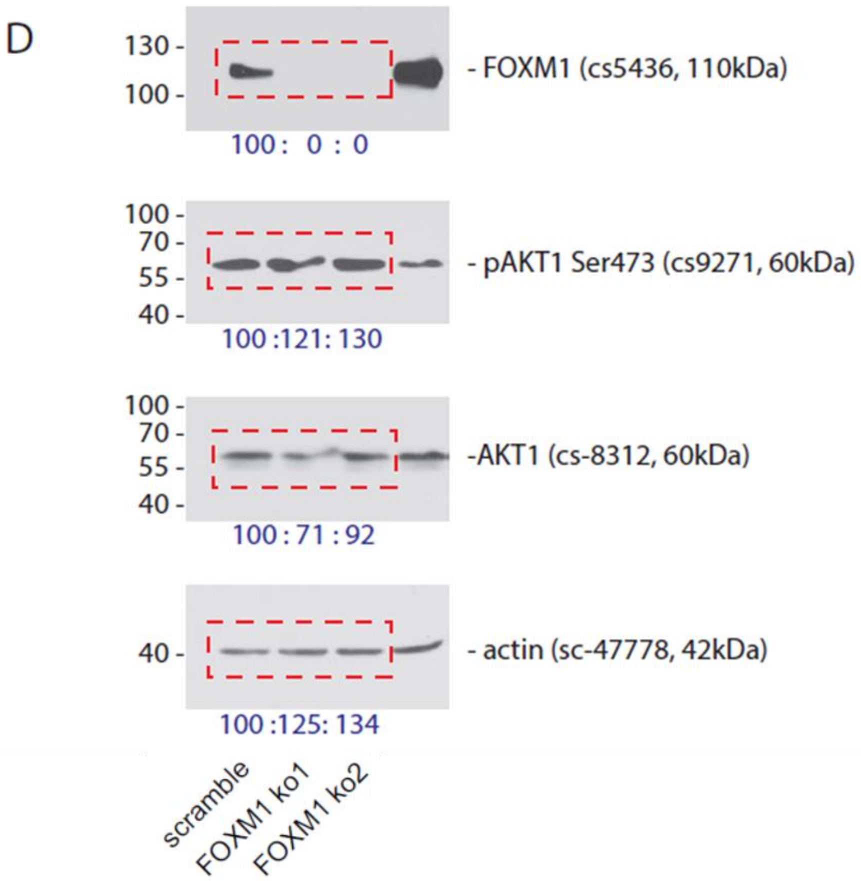
D
130 -
100 -
- FOXM1 (cs5436, 110kDa)
100 : 0 : 0
100 -
70 -
55 -
40 -
- pAKT1 Ser473 (cs9271, 60kDa)
100 :121: 130
100 -
70 -
55 -
40 -
-AKT1 (cs-8312, 60kDa)
100 : 71 : 92
40 -
- actin (sc-47778, 42kDa)
100 :125: 134
scramble
FOXM1 ko1
FOXM1 ko2

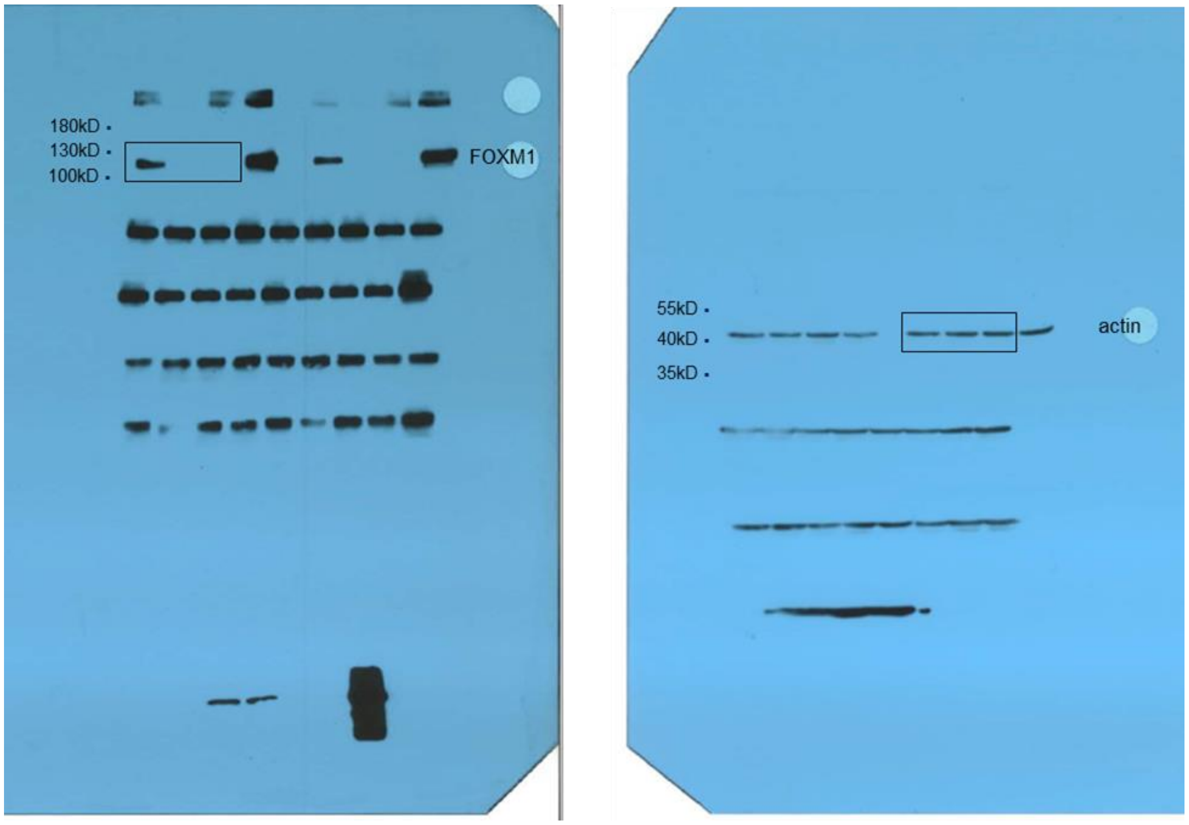
180kD
130kD
100kD
FOXM1
55kD
40kD
35kD
actin

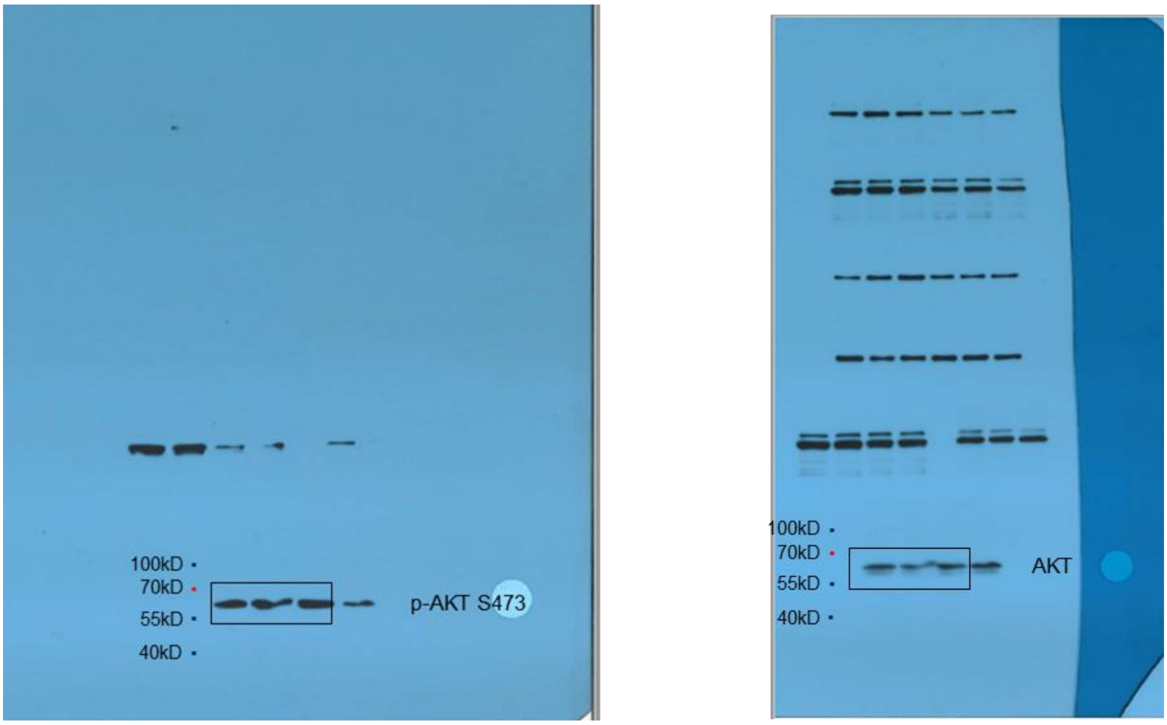

Figure S7. RASSF1A inhibits YAP1 and knockdown of YAP1 suppresses AKT1 activity, inhibits expression of ERα and FOXM1, and increases levels of FOXO3A.

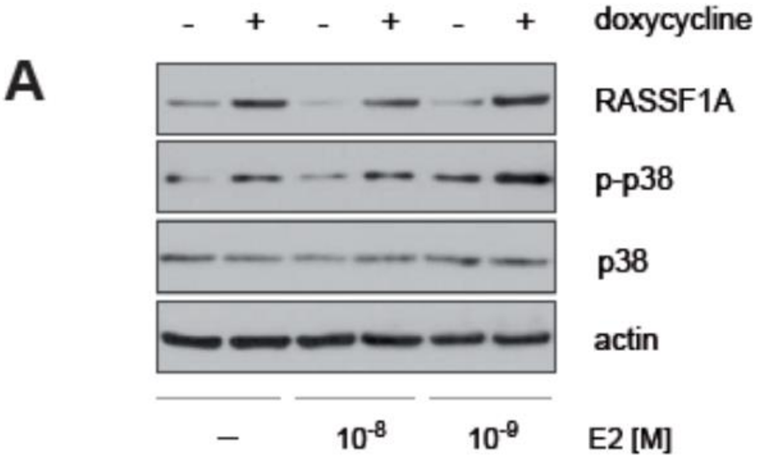
A
- + - + - + doxycycline
RASSF1A
p-p38
p38
actin
— 10-8 10-9 E2 [M]

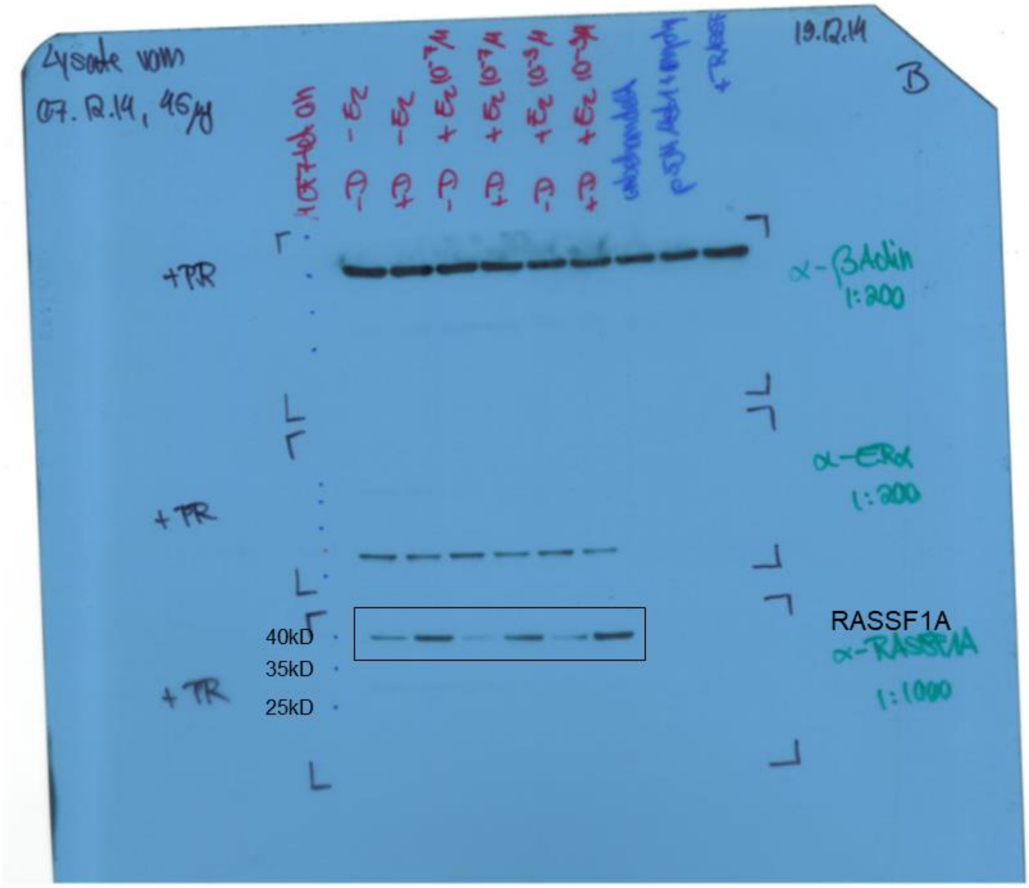
Lysate vom
07.12.14, 45µg
19.12.14
B
+PR
α-βActin
1:200
+PR
α-ERα
1:200
RASSF1A
40kD
35kD
25kD
+PR
α-RASSF1A
1:1000

Supplementary information 1A.

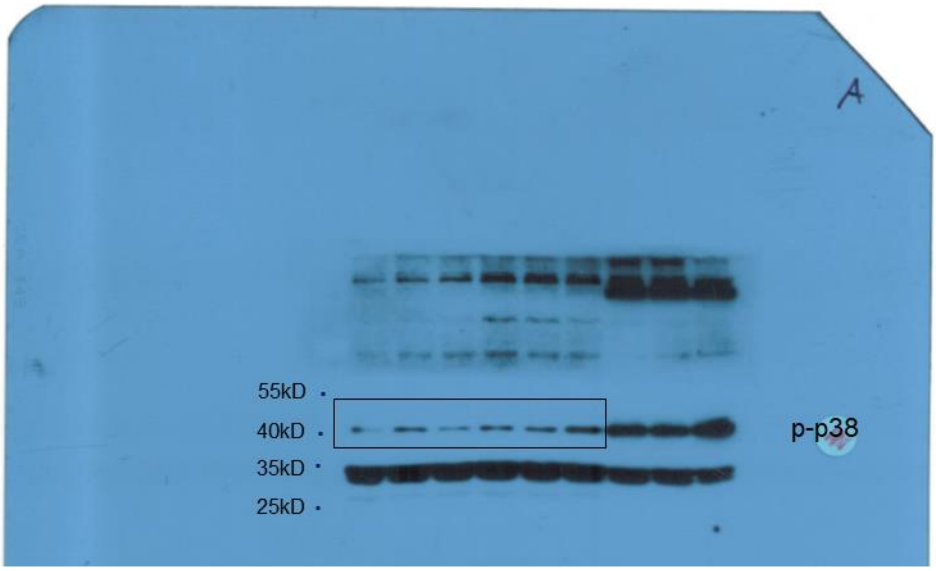

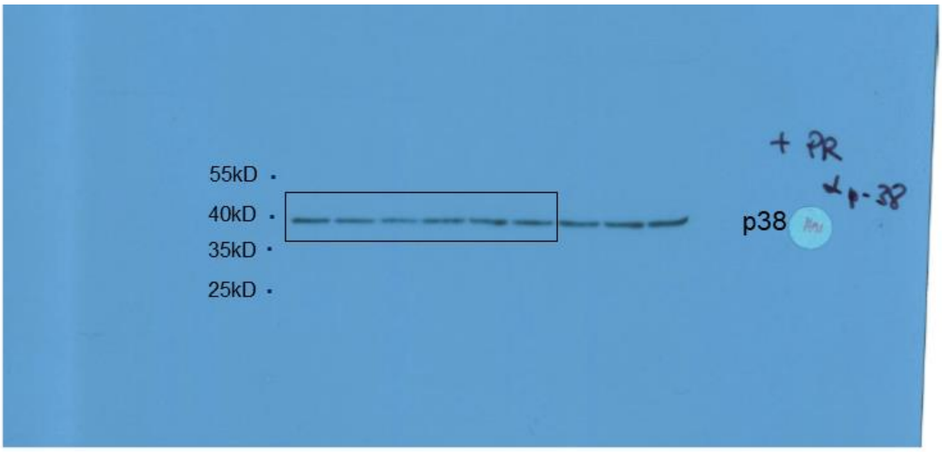

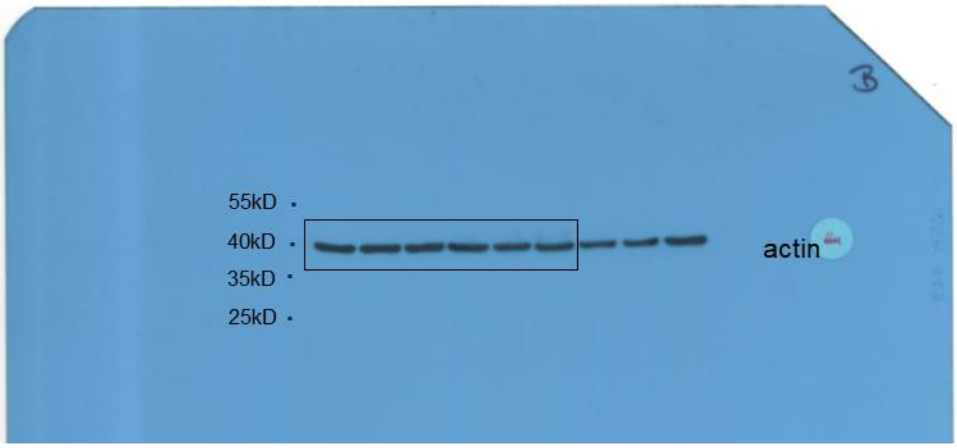

Supplementary information 1A.

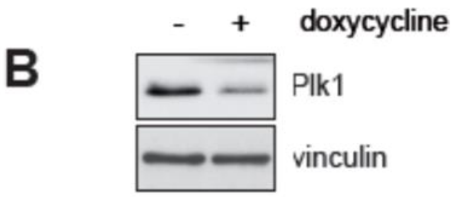

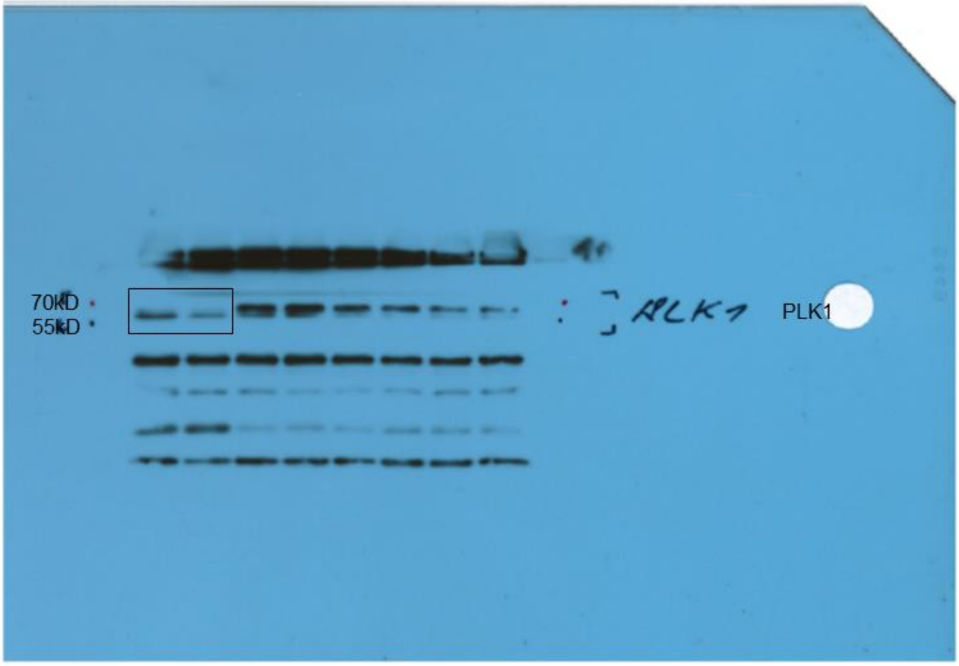

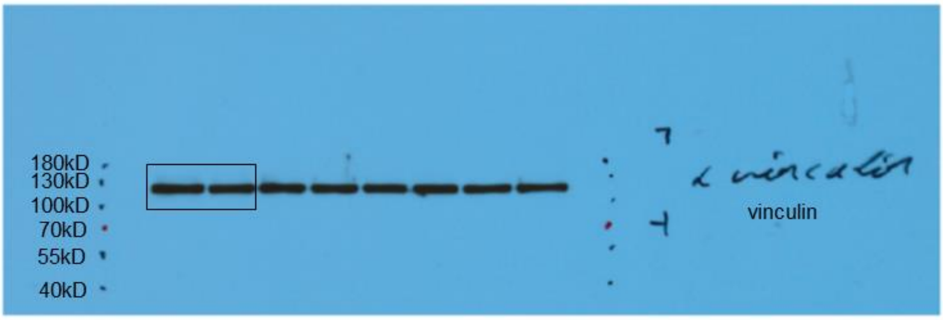

Supplementary information 1B.

Antibodies

RASSF1A Abcam [3F3] (ab23950), dilution: 1:1000
FOXM1 Cell Signaling # 5438, dilution: 1:500
Estrogen receptor alpha Santa Cruz (D-12) sc-8005, dilution: 1:200
β-actin Santa Cruz (C-4) sc-47778, dilution: 1:200
Akt1/2/3 Santa Cruz (H-136) sc-8312, dilution: 1:1000
FOXO3A Cell Signaling # 2497, dilution: 1:1000
Histon H3 (FL-136) sc-10809, dilution: 1:200
α-Tubulin Cell Signaling # 2144, dilution: 1:1000
p-Akt (Ser473) Cell Signaling # 9271, dilution: 1:1000
p-FOXO3A (Ser253) Cell Signaling # 9466, dilution: 1:1000
YAP Cell Signaling # 14074, dilution: 1:1000
p21 Santa Cruz sc-397, dilution: 1:200
vinculin Sigma Aldrich V9131, dilution: 1:1000
p38 Cell Signaling #9212, dilution 1:1000
p-p38 Cell Signaling #9215, dilution 1:1000
PLK1 Cell Signaling #4535, dilution 1:500

shRNAs

shFOXM1 ko-1 pLKO.1 puro Sigma Aldrich TRCN0000015544
5'-ccgggcccaacaggagtctaatcaactcgagttgattagactcctgttgggcttttt-3'
shFOXM1 ko-2 pLKO.1 puro Sigma Aldrich TRCN0000015546
5'-ccgggccaatcgttctctgacagaactcgagttctgtcagagaacgattggcttttt-3'
shFOXO3 ko-1 pLKO.1 puro Sigma Aldrich TRCN0000010335
5'-ccggcatgttcaatgggagcttggactcgagtccaagctcccattgaacatgtttttg-3'
shYAP1 ko-1 pLKO.1 puro Sigma Aldrich TRCN0000300282
5'-ccggcccagttaaatgttcaccaatctcgagattggtgaacatttaactgggtttttg-3'
shYAP1 ko-2 pLKO.1 puro Sigma Aldrich TRCN0000107266
5'-ccgggccaccaagctagataaagaactcgagttctttatctagcttggtggctttttg-3'

Primer

Estrogen Receptor alpha fw	att ggc cag tac caa tga caa ggg
Estrogen Receptor alpha rev	tat caa tgg tgc act ggt tgg tgg
FOXM1 fw	acc tgc agc tag gga tgt gaa tct
FOXM1 rev	aag cca ctg gat gtt gga tag gct
PLK-1 fw	aac gac ttc gtg ttc gtg gtg ttg
PLK-1 rev	ggt act ggc agc caa gca caa ttt
Cdk2 fw	ctg aaa tcc tcc tgg gct gca aat
Cdk2 rev	atc cgg aag agc tgg tca atc tca
Cdk1 fw	tgg cca gaa gtg gaa tct tta c
Cdk1 rev	att cgt ttg gct gga tca tag a
Cdk4 fw	gcc tct ctt ctg tgg aaa ctg
Cdk4 rev	tac atc tcg agg cca gtc at
Cdk6 fw	cct cgg agc agc tga aac at
Cdk6 rev	gaa cag aca gag aaa cca aac taa c
p21 Cip1/Waf1 fw	aag acc atg tgg acc tgt cac tgt
p21 Cip1/Waf1 rev	agg gct tcc tct tgg aga aga tca
RibPO fw	aga caa tgt ggg ctc caa gca gat
RibPO rev	gca tca tgg tgt tct tgc cca tca

Plasmide

pMx-pie [3,23]
pM-FOXM1-pie (this work)
pM-FOXO3A-pie (this work)
pM-MyrAkt1-ERtam-pie (this work)
pQCXIN (Takara, Clontech, Heidelberg, Germany)
pQC-FOXM1-IN (this work)
pQC-FOXO3A-IN (this work)
pcDNA3 RASSF1A (gift from R. Dammann)

FHRE Luc	Addgene Plasmid #1789	[6]
HA-FOXO3a WT	Addgene Plasmid #1787	[6]
Principal Investigator: Michael Greenberg		
pcDNA3 Myr-HA-AKT1	Addgene Plasmid #9008	[31]
Principal Investigator: William Sellers		
VP16-ER alpha	Addgene Plasmid #11351	[33]
VP16-ER beta (long)	Addgene Plasmid #11352	[33]
VP16-ER beta (short)	Addgene Plasmid #11353	[33]
3x ERE TATA luc	Addgene Plasmid #11354	[34]
Principal Investigator: Donald McDonnell		
IKK-2 WT	Addgene Plasmid #11103	[35]
IKK-2 K44M	Addgene Plasmid #11104	[35]
IKK-2 S117E S181E	Addgene Plasmid #11105	[35]
Principal Investigator: Adjana Rao		

© 2020 by the authors. Licensee MDPI, Basel, Switzerland. This article is an open access article distributed under the terms and conditions of the Creative Commons Attribution (CC BY) license (http://creativecommons.org/licenses/by/4.0/).
